# Genetic and Functional Studies Implicate Synaptic Overgrowth and Ring Gland cAMP/PKA Signaling Defects in the *Drosophila melanogaster* Neurofibromatosis-1 Growth Deficiency

**DOI:** 10.1371/journal.pgen.1003958

**Published:** 2013-11-21

**Authors:** James A. Walker, Jean Y. Gouzi, Jennifer B. Long, Sidong Huang, Robert C. Maher, Hongjing Xia, Kheyal Khalil, Arjun Ray, David Van Vactor, René Bernards, André Bernards

**Affiliations:** 1 Massachusetts General Hospital Center for Cancer Research and Harvard Medical School, Charlestown, Massachusetts, United States of America; 2 Center for Human Genetic Research, Massachusetts General Hospital, Boston, Massachusetts, United States of America; 3 Department of Cell Biology, Harvard Medical School, Boston, Massachusetts, United States of America; 4 Division of Molecular Carcinogenesis, The Netherlands Cancer Institute, Amsterdam, The Netherlands; The University of North Carolina at Chapel Hill, United States of America

## Abstract

Neurofibromatosis type 1 (NF1), a genetic disease that affects 1 in 3,000, is caused by loss of a large evolutionary conserved protein that serves as a GTPase Activating Protein (GAP) for Ras. Among *Drosophila melanogaster Nf1* (*dNf1*) null mutant phenotypes, learning/memory deficits and reduced overall growth resemble human NF1 symptoms. These and other *dNf1* defects are relatively insensitive to manipulations that reduce Ras signaling strength but are suppressed by increasing signaling through the 3′-5′ cyclic adenosine monophosphate (cAMP) dependent Protein Kinase A (PKA) pathway, or phenocopied by inhibiting this pathway. However, whether *dNf1* affects cAMP/PKA signaling directly or indirectly remains controversial. To shed light on this issue we screened 486 1^st^ and 2^nd^ chromosome deficiencies that uncover >80% of annotated genes for dominant modifiers of the *dNf1* pupal size defect, identifying responsible genes in crosses with mutant alleles or by tissue-specific RNA interference (RNAi) knockdown. Validating the screen, identified suppressors include the previously implicated *dAlk* tyrosine kinase, its activating ligand *jelly belly* (*jeb*), two other genes involved in Ras/ERK signal transduction and several involved in cAMP/PKA signaling. Novel modifiers that implicate synaptic defects in the *dNf1* growth deficiency include the intersectin-related synaptic scaffold protein Dap160 and the cholecystokinin receptor-related CCKLR-17D1 drosulfakinin receptor. Providing mechanistic clues, we show that *dAlk*, *jeb* and *CCKLR-17D1* are among mutants that also suppress a recently identified *dNf1* neuromuscular junction (NMJ) overgrowth phenotype and that manipulations that increase cAMP/PKA signaling in adipokinetic hormone (AKH)-producing cells at the base of the neuroendocrine ring gland restore the *dNf1* growth deficiency. Finally, supporting our previous contention that ALK might be a therapeutic target in NF1, we report that human *ALK* is expressed in cells that give rise to NF1 tumors and that NF1 regulated ALK/RAS/ERK signaling appears conserved in man.

## Author Summary

Neurofibromatosis type 1 (NF1) is a genetic disease that affects 1 in 3,000 and that is caused by loss of a protein that inactivates Ras oncoproteins. NF1 is a characteristically variable disease that predisposes patients to several symptoms, the most common of which include benign and malignant tumors, reduced growth and learning problems. We and others previously found that fruit fly mutants that lack a highly conserved *dNf1* gene are reduced in size and exhibit impaired learning and memory, and that both defects appear due to abnormal Ras and cyclic-AMP (cAMP) signaling. The former was unremarkable, but how loss of *dNf1* affects cAMP signaling remains poorly understood. Here we report results of a genetic screen for dominant modifiers of the *dNf1* growth defect. This screen and follow-up functional studies support a model in which synaptic defects and reduced cAMP signaling in specific parts of the neuroendocrine ring gland contribute to the *dNf1* growth defect. Beyond these results, we show that human ALK is expressed in cells that give rise to NF1 tumors, and that NF1 regulated ALK/RAS/ERK signaling is evolutionary conserved.

## Introduction

RASopathies, caused by mutations that activate Ras/ERK signaling, are a group of related disorders with features that include facial dysmorphism, skeletal, skin and cardiac defects, cognitive deficits, reduced growth and an increased cancer risk [1]. Neurofibromatosis type 1 (NF1; OMIM 162200), caused by loss of a RasGAP, and Noonan syndrome, caused by mutations that alter Ras/ERK pathway proteins SOS1, KRAS, NRAS, RAF1, BRAF, CBL, PTPN11, or SHOC2, are the most common members of this group, affecting 1 in 3,000, or as many as 1 in 1,000 live births, respectively [2], [3]. The genetics of these disorders provides a strong argument that excess Ras/ERK signaling underlies common RASopathy symptoms, and much effort remains focused on attenuating Ras/ERK signaling as a strategy for therapeutic intervention. However, whether life-long pharmacological inhibition of Ras/ERK signaling is a viable strategy to treat the full range of often non-life-threatening, but nonetheless serious symptoms of these chronic disorders, remains an open question. This motivates our work to better understand the molecular and cellular pathways responsible for NF1 symptom development, in the hope this will identify more specific therapeutic targets.

We have been interested in using *Drosophila melanogaster* as a model to investigate NF1 functions *in vivo*, following our identification of a conserved *dNf1* ortholog predicting a protein that is 60% identical to human neurofibromin over its entire 2802 amino acid length [4]. Like human neurofibromin, the Drosophila protein functions as a GAP for conventional (dRas1) and R-Ras-like (dRas2) GTPases [4], [5]. This functional conservation made it all the more surprising when both initially identified *dNf1* homozygous null mutant phenotypes, a postembryonic growth deficiency and a neuropeptide-elicited NMJ electrophysiological defect, appeared insensitive to genetic manipulations that attenuate Ras signaling strength, but were suppressed by increasing signaling through the cAMP-dependent PKA pathway [4], [6]. The genetic link between *dNf1* and cAMP/PKA led to further studies, which demonstrated that similar to many children with NF1 [7], and *Nf1^+/−^* mice [8], *dNf1^−/−^* flies exhibit specific learning and memory deficits [9]. Biochemical studies with fly brain extracts further revealed that loss of *dNf1* is associated with reduced GTP-γS-stimulated but not basal adenylyl cyclase (AC) activity [9], and with defects in both classical and unconventional AC pathways [10]. Arguing that the cAMP related function of NF1 is evolutionary conserved, GTP-γS-stimulated AC activity and cAMP levels were also reduced in E12.5 *Nf1^−/−^* mouse brain [11], and defects in cAMP generation appear to explain the unique sensitivity to *Nf1* heterozygosity of murine central nervous system neurons [12]. Arguing that NF1 may regulate cAMP signaling at least in part in a cell autonomous manner, reduced cAMP levels and AC activity were also found in *NF1* deficient human astrocytes [13]. Thus, while there is little doubt that aberrant AC signaling is an evolutionary conserved *NF1* phenotype, we and others have reached conflicting conclusions about the underlying mechanism.

Based on Drosophila phenotypic rescue studies with human *NF1* transgenes, others reported that neurofibromin has physically separable functions as a negative regulator of Ras and a positive mediator of AC/PKA signaling. This conclusion followed from findings that NF1-GAP activity was not required to rescue *dNf1* size [10] or learning [14] phenotypes, whereas a transgene encoding a C-terminal part of human neurofibromin that did not include the GAP catalytic domain did suppress both defects. In obvious conflict, in similar experiments with *dNf1* transgenes, we found that neuronal expression of a functional NF1-GAP catalytic segment was necessary and sufficient to suppress the systemic growth defect, and that other protein segments had no effect. Moreover, the *dNf1* growth defect was also suppressed by neuronal expression of the Drosophila p120RasGAP ortholog, and although we extended earlier findings by showing that heterozygous loss of *dRas1* or *dRas2*, or of a comprehensive set of Ras effector proteins did not modify the growth defect, these mutations also did not reduce the elevated phospho-ERK level in the *dNf1* central nervous system (CNS). However, some Ras/ERK pathway double mutants did suppress both defects, leading us to conclude that excess neuronal Ras/ERK signaling is the proximal cause of the non-cell-autonomous *dNf1* growth defect [5]. Further supporting this notion, recent work implicated the neuronal *dAlk* tyrosine kinase receptor and its activating ligand *jelly belly* (*jeb*) as rate-limiting activators of *dNf1* regulated Ras/ERK pathways responsible for both systemic growth and olfactory learning defects [15].

The above evidence underlies our hypothesis that loss of *dNf1* increases neuronal dAlk/Ras/ERK activity, which in turn causes reduced cAMP/PKA signaling, which may or may not be cell-autonomous. Obviously, identifying additional components of *dNf1*-regulated growth controlling pathways followed by functional analysis might help to test this hypothesis. Here we report results of a *dNf1* growth deficiency modifier screen, which identified components of tyrosine kinase/Ras/ERK and neuropeptide/cAMP/PKA pathways in addition to genes involved in synaptic morphogenesis and functioning. Further analysis showed that the requirement for *dNf1* and cAMP/PKA in Drosophila growth regulation involves different tissues, with *dNf1* required broadly in larval neurons, and cAMP/PKA signaling specifically in AKH-producing cells and perhaps in other parts of the neuroendocrine ring gland. These results, and the recent discovery of a novel *dNf1* synaptic overgrowth phenotype [16] that is also suppressed by several genes identified in our screen, set the stage for further work to more precisely define how loss of *dNf1* causes Ras/ERK and other signaling defects, the ultimate consequence of which is reduced systemic growth.

## Results

### Loss of *dNf1* Does Not Phenocopy Starvation or Alter Developmental Timing

**Figure 1:**
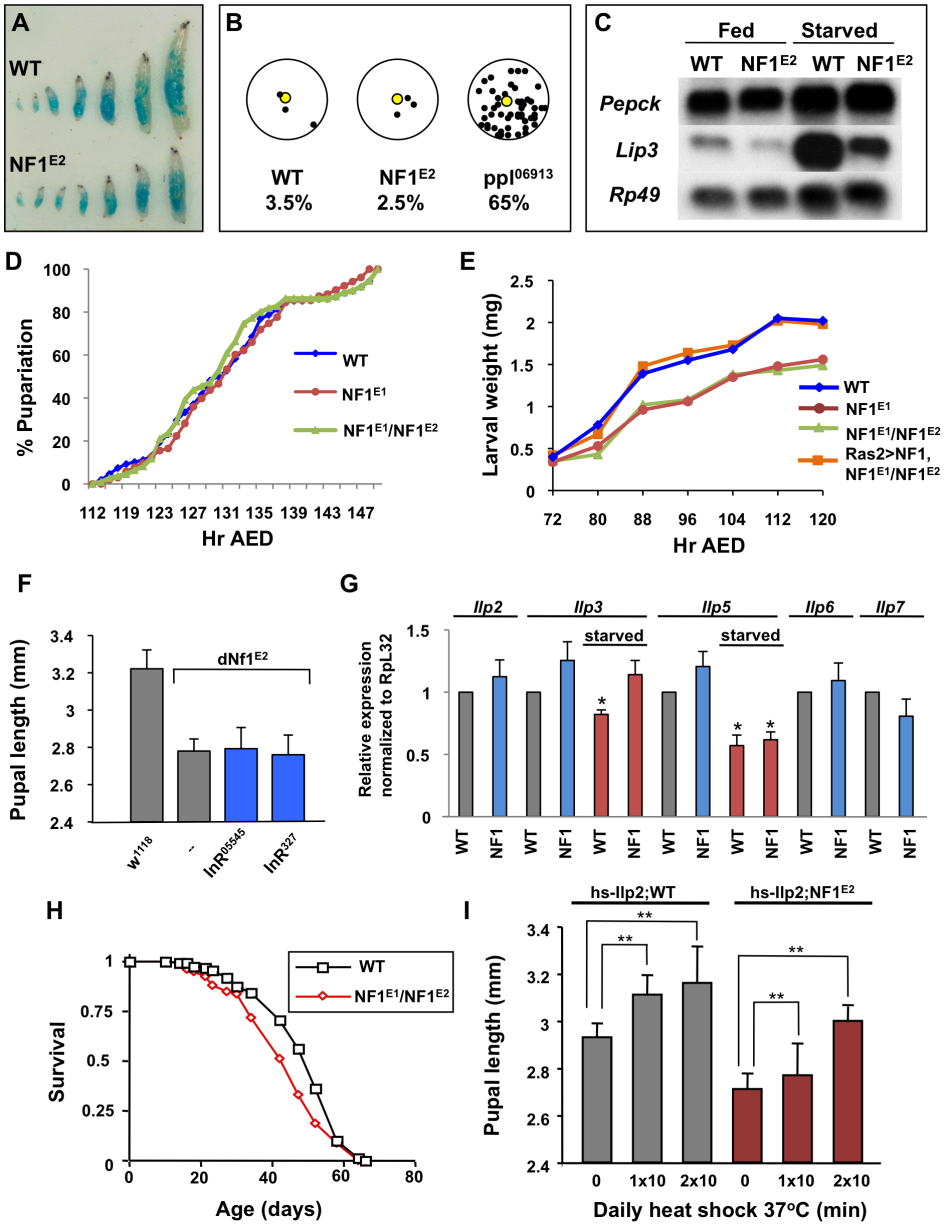
Loss of *dNf1* does not phenocopy starvation or alter developmental timing. (A) Wild-type (*w^1118^*) and *dNf1* larvae ingest similar amounts of food. Larvae at different stages of development were photographed after 25 minutes of feeding on dye-colored yeast paste. (B) As opposed to *ppl* mutants, wild-type and *dNf1* larvae do not wander from a food source (fraction of wandering larvae: WT 3.5% (SD 0.007), *dNf1* 2.5% (SD 0.007) and *ppl* 65% (SD 0.057)). In a similar assay, *dNf1* larvae also showed no abnormality in moving towards a food source (not shown). (C) RNA blot analysis of the starvation-sensitive genes, PEPCK and *Lip3* shows that *dNf1* larvae do not show elevated levels of either mRNA under normal feeding conditions. (D) Wild-type and *dNf1* larvae show no significant differences in developmental timing, as assessed by time of pupariation after egg deposition (AED). (E) The *dNf1* growth rate, as assessed by larval weight, is reduced throughout larval development when compared to wild-type or a *Ras2>UAS-dNf1* control. (F) Two hypomorphic insulin receptor alleles, *InR^05545^* and *InR^327^*, do not modify *dNf1* pupal size. (G) ILP mRNA expression is not obviously reduced in *dNf1* larvae. H) *dNf1* adult flies show no altered longevity compared to wild-type controls. (I) Over-expression of *Ilp2* from a *hs-Ilp2* transgene in *dNf1* larvae results in a similar increase in size as in wild-type flies.

Animals use elaborate hormonal mechanisms to coordinate nutrient availability and feeding with changes in metabolism and overall growth. Since starvation or crowding during the larval phase of the Drosophila life cycle reduces systemic growth [17], we first examined whether the small size of *dNf1* mutants reflected reduced feeding. Arguing against this hypothesis, wild-type and *dNf1* larvae ingested similar amounts of dye-stained food throughout their development (Figure 1A). Unlike a *pumpless* (*ppl*) mutant [18], *dNf1* larvae also showed no tendency to move away from a food source (Figure 1B). Analysis of the expression of the starvation-inducible *Pepck* and *Lip3* genes [18] provided further evidence that loss of *dNf1* does not phenocopy starvation (Figure 1C).

Mechanisms that control Drosophila growth have been the topic of intense study and much has been learned about how an interplay between insulin-like peptide (ILP) controlled growth rate and ecdysone controlled growth duration determines overall growth (see [19] and [20] for reviews). Arguing against an important role for ecdysone or other factors that control the length of the larval growth period, no differences in the expression of canonical ecdysone-regulated genes was found (results not shown) and no difference in developmental timing between wild-type and *dNf1* mutants was detected (Figure 1D and S1). Rather, a reduced growth rate throughout larval development results in an approximately 25% weight reduction of *dNf1* pupae relative to isogenic controls (Figure 1E and S1).

Drosophila ILPs control systemic growth, metabolism, longevity, and female fecundity [21]–[24]. Among the eight Drosophila ILP genes, *Ilp2*, *Ilp3* and *Ilp5* are co-expressed in bilateral clusters of seven insulin-producing neurosecretory cells (IPCs) in the larval brain [21]. Ablation of these cells causes a severe reduction in overall size, which is rescued by inducing the expression of a *hsp70-Ilp2* transgene [22], [23]. However, several results argue against a role for ILPs in the *dNf1* growth defect. Firstly, two hypomorphic insulin receptor alleles, *InR^05545^* and *InR^327^*, did not affect *dNf1* pupal size (Figure 1F). Secondly, qRT-PCR analysis of RNA extracted from wandering wild-type and *dNf1* third instar larvae detected no major differences in the expression of *Ilp1* (not shown), *Ilp2*, *Ilp3*, *Ilp5*, *Ilp6* and *Ilp7* in fed larvae. Among the three IPC expressed ILP genes, the expression of *Ilp3* and *Ilp5* is reduced in response to starvation [21]. Starved wild-type and *dNf1* larvae showed a similar reduction in *Ilp5* expression, whereas *Ilp3* showed a less pronounced response (Figure 1G). Thirdly, while certain insulin receptor or insulin receptor substrate (*chico*) mutants have an up to 85% increased life span [25], [26], the lifespan of *dNf1* mutants and isogenic controls was comparable (Figure 1H). We note that others previously reported a reduced life span for the originally identified *dNf1 p*-element alleles, generated in a different genetic background [27]. Finally, we previously showed that *Ilp2-GAL4* driven *UAS-dNf1* expression in IPCs did not rescue the *dNf1* size defect [5]. Although daily heat shocking of *hsp70-ilp2* carrying larvae increased the size of *dNf1* pupae, indicating that mutants do not lack the ability to respond to insulin, similar induction of this transgene, as previously noted [21], also substantially increased the size of wild-type controls (Figure 1I). Thus, reduced insulin signaling does not provide an obvious explanation for the slower *dNf1* growth rate, prompting us to perform a screen to identify other genes involved in *dNf1*-mediated systemic growth control.

### Screen for Dominant Modifiers of *dNf1* Systemic Growth Phenotype

**Figure 2:**
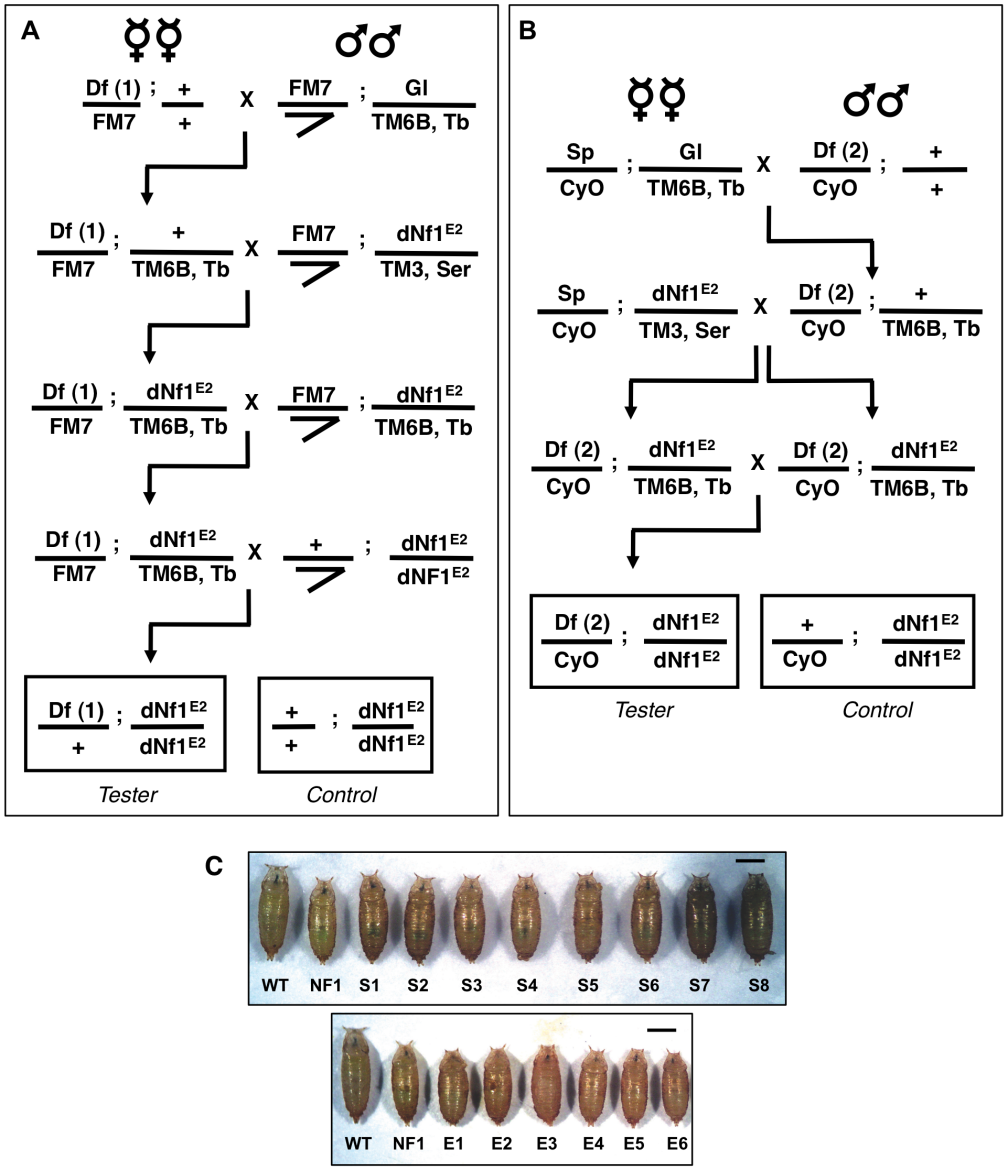
Deficiency screen for dominant modifiers of the *dNf1* growth defect. Isogenic 1^st^ and 2^nd^ chromosomes deficiencies from the Exelixis, DrosDel and Bloomington Stock Center collections were tested for their ability to alter *dNf1* female pupal size. Crossing schemes to generate *Df(1)/+; dNf1^E2^* (A) and *Df(2)/CyO; dNf1^E2^* (B) screening stocks. The *tubby*-marked *TM6B* 3^rd^ chromosome balancer allowed the selection of *dNf1^E2^* homozygotes for measurements. (C) Examples of deficiencies that suppress or enhance the *dNf1* size defect. Scale bar = 1 mm.

**Figure 3:**
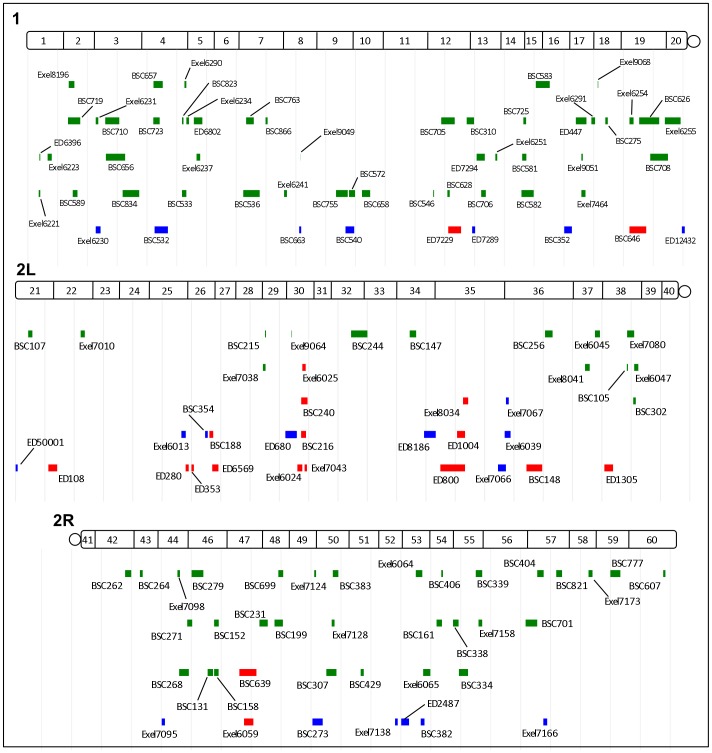
Cytogenetic locations of *dNf1* modifying deficiencies. Locations of modifying deficiencies (drawn to scale) on the 1^st^ and 2^nd^ (2L and 2R) chromosomes. Deficiencies that enhance or suppress are shown in red and green, respectively. Non-specific deficiencies that dominantly affect the size of wild-type pupae are in blue. Many modifying deficiencies uncover overlapping genomic segments.

**Figure 4:**
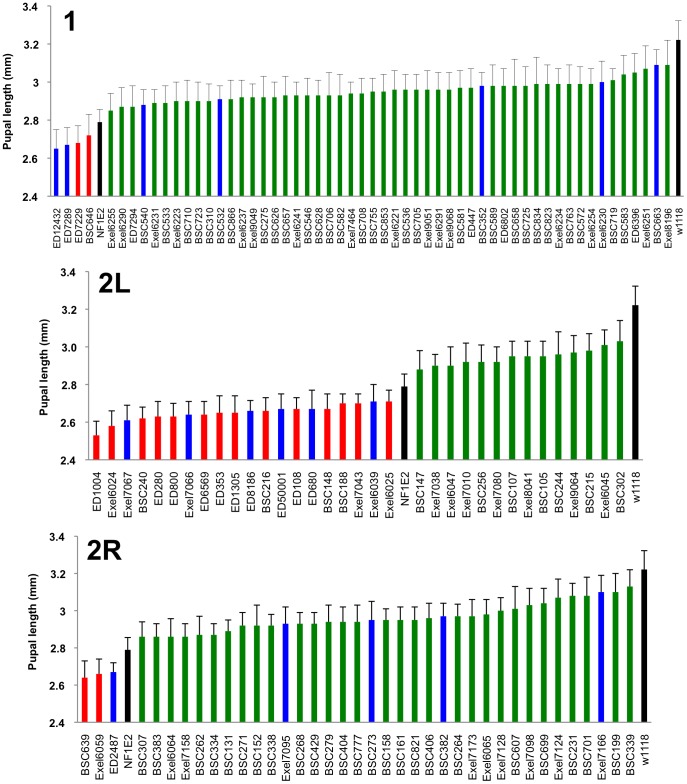
Identified deficiencies increase or decrease pupal size to different extents. Female pupal lengths for the indicated 1, 2L and 2R deficiencies. Control measurements for *dNf1^E2^* and wild-type (*w^1118^*) are in black. Colors for enhancing, suppressing and non-specific deficiencies are as in Figure 2. Pupal lengths are shown in mm, error bars denote standard deviations and are based on measurements described in Table S2. All shown deficiencies modify *dNf1* female pupal size with *p*-values<0.01.

While most *dNf1* defects are poorly suited for use in modifier screens, the postembryonic growth defect is robust and readily quantified during the pupal stage [4]. However, using this phenotype in a screen is complicated by the fact that organismal size is sexually dimorphic (females are larger than males) and affected by population density, feeding, environmental factors and genetic background differences. With these confounding factors in mind, we used the crossing schemes outlined in Figure 2 to test collections of isogenic 1^st^ and 2^nd^ chromosome deficiencies for *dNf1^E2^* pupal size modifier effects or synthetic lethal interactions. For each of 139 1^st^ and 347 2^nd^ chromosome deficiencies from the Exelixis [28], DrosDel [29] or Bloomington Stock Center (BSC) collections, we generated *Df(1)/+; Nf1^E2^/Nf1^E2^* (Figure 2A) or *Df(2)/+; Nf1^E2^/Nf1^E2^* (Figure 2B) stocks, respectively. Notably, our work identified only few synthetic lethal interactions, and in all cases tested the synthetic lethality has been specific to the chromosome carrying the *Nf1^E2^* allele, and not observed when the same deficiency was tested in *Nf1^E2^/Nf1^E1^* null trans-heterozygotes [5]. To guard against size differences caused by inadvertent differences in population density or environmental conditions, each deficiency was scored at least twice using an initial rough caliper measurement of pupae attached to the side of culture vials. For each candidate modifying deficiency thus identified, microscopy combined with image analysis was used to determine the precise head-to-tail length of at least 40 pupae, which were then allowed to individually eclose in order to establish their sex. Several controls were next performed to eliminate non-specific modifiers or artifactual results. First, for all suppressors the continued presence of the *Nf1^E^* ^2^ nonsense mutation was confirmed by a PCR assay (Figure S2). Secondly, as a critical specificity control, all modifying deficiencies were analyzed in a wild-type background to eliminate those that affect pupal size irrespective of *dNf1* genotype. Further analysis of some of these non-specific modifiers demonstrated that loss of *Act57B* dominantly increases pupal size, whereas heterozygous loss of the glutamate transporter *Eaat1* has the opposite effect. Thirdly, because pupal size is a function of larval growth rate and duration, modifying deficiencies were monitored for obvious changes in developmental timing. Table 1 shows the number of screened chromosome 1, 2L and 2R deficiencies, the fraction of genes uncovered and the number of *dNf1* and wild-type pupal size modifying deficiencies and loci identified. Figure 2C shows the magnitude of the pupal size modification of typical enhancers and suppressors. The number of modifying deficiencies exceeds the number of identified loci, because many modifying deficiencies uncover overlapping genomic segments (Figure 3). Not unexpectedly, individual modifying deficiencies increase or decrease *dNf1* pupal size to different extents (Figure 4).

**Table 1:** Deficiency screen summary.

Chromosome	Number screened	% genes uncovered	*dNf1* Modifiers		Non-specific modifiers		*dNf1* modifying loci	
			SUP	ENH	SUP	ENH	SUP	ENH
1	139	82.1	48	2	5	2	30	2
2L	182	87.7	14	15	1	7	11	10
2R	165	86.9	31	2	4	1	22	1

Indicated are the number of chromosome 1, 2L and 2R deficiencies screened, the fraction of genes uncovered (based on the FB2013_03 FlyBase release), the number of *dNf1* modifying deficiencies and loci identified, and the number of non-specific modifiers.

Some large non-modifying deficiencies identified in our screen completely overlapped with smaller modifying ones. In such cases, stocks were re-ordered and reanalyzed. If these tests replicated the original results, genetic complementation analysis or PCR amplification using transposon and flanking sequence-specific primers was used to confirm the mapping of the deficiencies in question. This procedure identified several mismapped or mislabeled deficiencies, most of which have since been withdrawn by stock centers. Any suspect or recessive modifying deficiency, or any deficiency that uncovers genes with non-specific size phenotypes, such as *Minute* loci [30], [31], were eliminated from further analysis. Table S1 lists these deficiencies and the reason for their exclusion.

During work to identify genes responsible for observed effects, we prioritized genes uncovered by suppressing deficiencies over those uncovered by enhancers. We also prioritized modifying loci uncovered by more than one deficiency, strong modifiers over weak ones, and genes uncovered by smaller deficiencies over those uncovered by larger ones, reasoning that effects of smaller deficiencies are more likely due to the loss of single genes. Validating the screen, suppressing *Df(2R)Exel7144* uncovers *dAlk* and partially overlapping suppressing *Df(2R)BSC199* and *Df(2R)BSC699* each uncover the gene for its activating ligand, *jeb*, both previously identified as dominant suppressors of *dNf1* size, learning, and neuronal ERK over-activation phenotypes [15]. Other uncovered candidate modifiers, such as PKA catalytic and regulatory subunit genes, were tested in crosses with loss-of-function alleles and/or by tissue-specific knockdown using at least two independent UAS-RNAi transgenes, most of which were obtained from the Vienna Drosophila Stock Center (VDRC) [32]. For deficiencies that lacked obvious candidate modifiers, we used the UAS-RNAi approach to more broadly screen uncovered genes. Figure S3 shows examples of modifiers identified by this latter approach. Although the nutrient sensing fat body and other tissues outside of the CNS play important roles in Drosophila growth control [33], [34], candidate modifiers have only been tested by RNAi knockdown in neurons or glial cells. We focused on these cell types, because neuronal *UAS-dNf1* expression sufficed to suppress the growth phenotype [5].

The *dNf1* pupal size modifiers identified to date can be classified into three non-exclusive categories, the first of which consists of the previously implicated *dAlk/jeb* receptor/ligand pair and two not previously implicated other genes involved in Ras-mediated signal transduction. Another expected category includes genes involved in cAMP/PKA signaling, including the previously reported *dnc* cAMP phosphodiesterase suppressor [35], and the newly identified PKA catalytic subunit gene, *PKA-C1*, which acts as an enhancer. This group also includes the *CCKLR-17D1* drosulfakinin receptor, recently implicated as a cAMP-coupled promoter of synaptic growth [36], which is particularly interesting given the recent identification of a *dNf1* larval NMJ overgrowth phenotype [16]. Finally, our screen also identified multiple genes whose roles in *dNf1* growth control had not been anticipated and whose functional relevance remains to be established. Several genes in this group are predominantly expressed in brain or have known neuronal functions, including genes coding for the aforementioned CCKLR-17D1 receptor, the synaptic scaffold protein Dap160, the neuronal RNA binding protein elav, the neuronal Na,K ATPase interacting protein NKAIN [37], and the larval brain and alimentary channel expressed amino acid transporter NAAT1 [38]. Other genes in this group include *CKIIbeta2*, encoding a casein kinase regulatory subunit, the endosomal trafficking proteins *deep-orange* and *carnation*, the *Notch* modifier heparan sulfate 3-O sulfotransferase *Hs3st-B* [39], and the ubiquitin E3 ligases *HERC2*, *which acts as a suppressor*, and CUL3, which has the opposite effect. Table 2 lists deficiencies that modify *dNf1* but not wild-type pupal size, limited to those for which the responsible gene has been identified. Table S2 identifies all analyzed deficiencies, indicates which modified *dNf1* pupal size (providing female pupal sizes as a gauge of modification strength), which also altered wild-type pupal size, and which deficiencies altered developmental timing.

**Table 2:** Modifying deficiencies and identification of responsible genes.

Deficiency	Cytological Breakpoints	Modif.	Gene Implicated	Modifying allele(s) and/or RNAi
**Tyrosine Kinase/Ras signaling**				
Df(2R)Exel7144 Df(2R)Exel6064	53C8;53D2 53C11;53D11	SUP	*dAlk*	*dAlk^8^* (lof), *dAlk^9^* (lof), *v11446*, *v107083*, *JF02668*
Df(2R)BSC199 Df(2R)BSC699	48C5;48E4 48D7;48E6	SUP	*Jellybelly (jeb)*	*Jeb^weli^* (lof), *v103047*, *v30800*
Df(2R)BSC161	54B2;54B17	SUP	*connector enhancer of ksr (cnk)*	*cnk^XE-385^* (Δ), *cnk^E-2083^* (lof), *v107746*
Df(1)BSC663 Df(1)Exel9049	8D1;8D5 8D2;8D3	SUP	*Dsor1* and *almondex (amx)*	*Dsor1: Dsor1^LH110^* (amorph), *v107276*, *v40026*, *HMS00145*; amx: *amx^f06362^* (hypo), *v3296*
**cAMP/PKA signaling**				
Df(1)BSC710 Df(1)BSC656 Df(1)BSC834	3B2;3C9 3B3;3D2 3C11;3F3	SUP	*dunce (dnc)*	*dnc^M14^* (amorph), *dnc^ML^* (amorph), *dnc^1^*(hypo)
Df(2L)Exel6024	30C1;30C9	ENH	*cAMP-dependent protein kinase 1 (PKA-C1)*	*PKA-C1^BG02142^* (leth), *PKA-C1^06353^* (hypo), *PKA-C1^B3^* (leth)
**Neuronal Function**				
Df(1)ED447 Df(1)Exel9051 Df(1)Exel7464	17C1;17F1 17D1;17D3 17D1;17E1	SUP	*CCK-like receptor at 17D1 (CCKLR-17D1)*	*v100760*
Df(2L)BSC302 Df(2L)Exel6047	39A1;39A6 39A2;39B4	SUP	*Dynamin-associated protein 160 (Dap160)*	*Dap160* ^Δ*1*^ (lof), *Dap160* ^Δ*2*^ (lof), *v106689*, *v16158*, *JF01918*
Df(1)Exel6221 Df(1)ED6396	1B4;1B8 1B5;1B8	SUP	*Embryonic lethal abnormal vision (elav)*	*elav^G0031^, elav^1^*
Df(2L)BSC216 Df(2L)BSC240 Df(2L)Exel7043 Df(2L)Exel6025	30C6;30E1 30C7;30F2 30D1;30F1 30C9;30E1	ENH	*Nicotinic Acetylcholine Receptor alpha-30D (nAcRα-30D)*	*nAcRα-30D^DAS1^* (via) *nAcRα-30D^DAS2^* (via) *nAcRα-30D^KG05852^* (via)
Df(2L)Exel8041	37D7;37F2	SUP	*Rab9*	*v107192*, *v36200, HMS02635*
**Other**				
Df(1)Exel6254	19C4;19D1	SUP	*HERC2*	*v105374*
Df(2L)ED800 Df(2L)ED1050 Df(2L)ED1004	35B2;35D1 35B8;35D4 35B10;35D1	ENH	*Cullin-3 (cul3)*	*cul3^gft2^* (lof)
Df(1)BSC533 Df(1)Exel6290	4F4;4F10 4F7;4F10	SUP	*Neutral amino acid transporter 1 (NAAT1)*	*v106027*, *v37380, v50063*
Df(1)Exel9068	18B4;18B6	SUP	*Heparin sulfate 3-O sulfotransferase-B (Hs3st-B)*	*v110601*
Df(2R)BSC701	56F15;57A9	SUP	*Casein kinase II β2 subunit (CKIIβ2)*	*v102633, v26915*
Df(2R)BSC607	60E4;60E8	SUP	*Na,K-ATPase Interacting (NKAIN)*	*v105893, v102018*
Df(1)BSC275	18C8;18D3	SUP	*Vps33/carnation (car)*	*car^1^* (hypo), *car* ^Δ*146*^ (lof), *v110756*
Df(1)BSC719 Df(1)Exel8196 Df(1)BSC589	2A3;2B13 2B1;2B5 2B3;2B9	SUP	*Vps18/deep orange (dor)*	*dor^8^* (leth), *v107053*, *v105330*

Modifying deficiencies for which the responsible *dNf1* interacting gene has been identified. The cytological location, and the dominant effect on *dNf1* pupal size (SUP - suppressor, ENH – enhancer) of each deficiency is given. The responsible genes for each modifying deficiency are shown with the mutant alleles, VDRC and TRiP RNAi lines used in their identification. Expression of RNAi transgenes was induced with the *Ras2*-*Gal4*, *elav*-*Gal4*, *n-syb*-*Gal4* and/or C23-*Gal4* drivers. Abbreviations: hypo: hypomorphic; leth: lethal; lof: loss-of-function; amorph: amorphic; Δ: deletion; via: viable.

### *dNf1* Pupal Size Modifiers Involved in Jeb/dAlk/Ras/ERK Signaling

**Figure 5:**
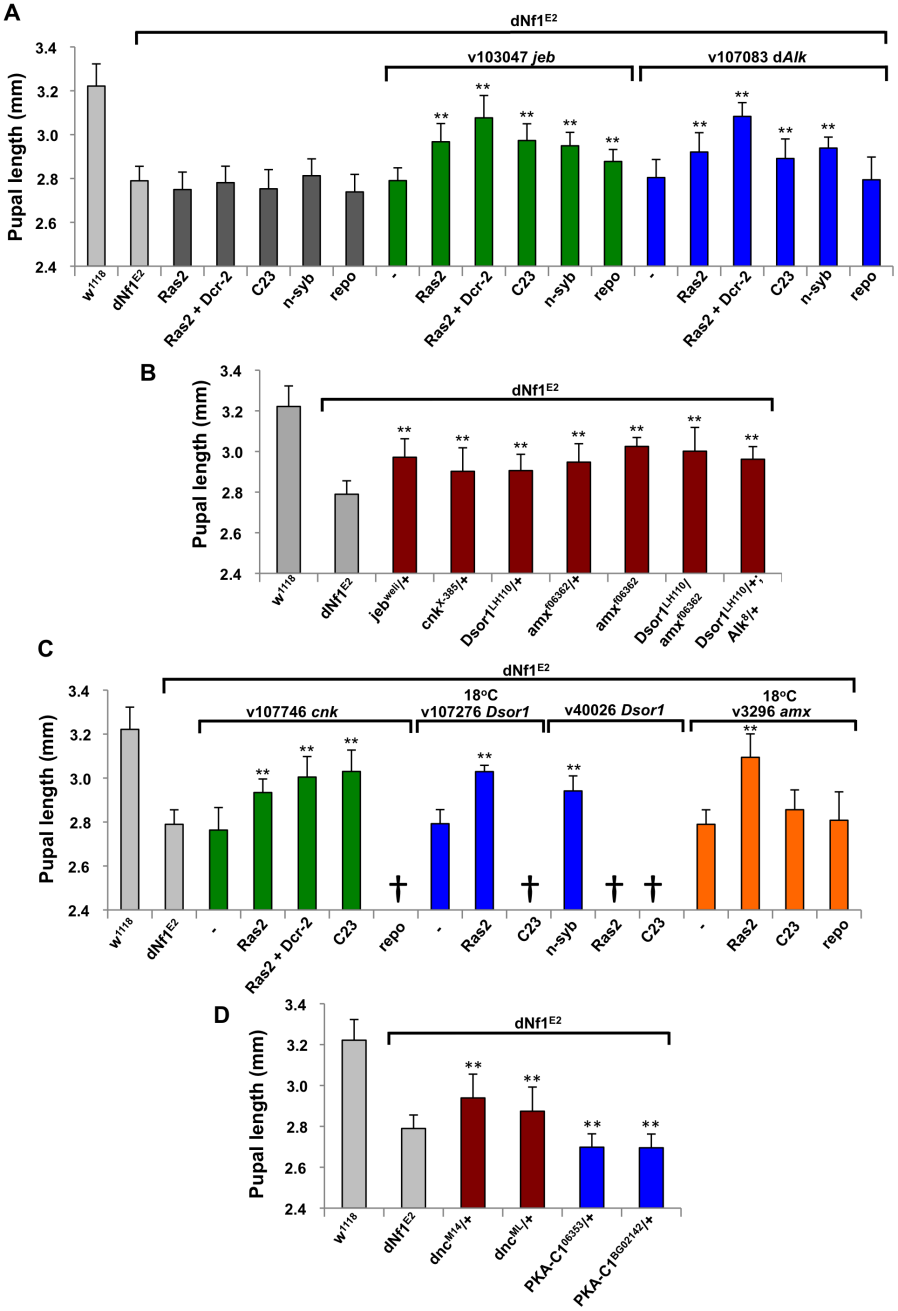
Validation of *dNf1* modifiers involved in Jeb/dAlk/Ras/ERK and cAMP signaling. (A) Neuronal expression of *dAlk* RNAi using *Ras2-Gal4*, *Ras2-Gal4*+*UAS-Dcr-2*, *c23-Gal4* or *n-syb-Gal4* drivers suppresses the *dNf1* size defect. Expression of *jeb* RNAi with the same neuronal drivers also suppresses. Weaker suppression is observed when *jeb* RNAi expression is controlled by the pan-glial *repo*-*Gal4* driver. Dark grey bars are control measurements of Gal4 drivers in the *dNf1* background. Light grey bars are sizes of wild-type (*w^1118^*) and *dNf1^E2^* controls. (B) Suppression of the *dNf1* size defect by the indicated *jeb*, *cnk*, *Dsor1* and *amx* alleles. (C) Neuronal *cnk*, *Dsor1* or *amx* knockdown suppressed the *dNf1* size defect. In the case of *Dsor1 v107276* and *amx*, cultures were maintained at 18°C to prevent lethality observed at 25°C. Some RNAi transgene/driver combinations were lethal (†) even at 18°C. (D) Validation of *dnc* and *Pka-C1* as *dNf1* modifiers was obtained in crosses with *dnc^M14^*, *dnc^ML^*, *Pka-C1^6353^* and *Pka-C1^BG02142^* loss-of-function alleles. In this and subsequent figures, * and ** denote *p*-values<0.05 and <0.01, respectively.

We previously reported that the *dAlk* receptor tyrosine kinase [40] acts as a rate-limiting activator of neuronal Ras/ERK pathways responsible for *dNf1* size and learning defects [15]. Therefore, the fact that the *dAlk* and *jeb* genes are uncovered by one and two suppressing deficiencies, respectively (Table 2), validates our screen. Others recently reported that Jeb/dAlk signaling allows brain growth to be spared at the expense of other tissues in nutrient restricted Drosophila, and identified a glial cell niche around neuroblasts as the source of Jeb under these conditions [41]. To determine whether glial cells also produce Jeb involved in overall growth control under normal conditions, we used glial and neuronal Gal4 drivers to test the effect of tissue-specific *jeb* and *dAlk* knockdown. Arguing that neurons are the main source of Jeb involved in systemic growth control under non-starvation conditions, *jeb* knockdown with the *Ras2-Gal4, C23-Gal4, and n-syb-Gal4* neuronal drivers [5] increased *dNf1^E2^* pupal size (Figure 5A), whereas the *Nrv2-Gal4, Eaat1-Gal4 and Gli-Gal4* glial drivers had no effect (data not shown). The only glial driver that gave rise to partial rescue was the pan-glial *repo*-*Gal4* line, although this effect was not enhanced by co-expressing *UAS-Dcr2*. Control experiments showed that any driver used in these and other experiments had no effect on pupal size in the absence of UAS transgenes or vice-versa, that UAS transgenes had no effect in the absence of Gal4 drivers (Figure 5A and data not shown). Finally, extending previous findings and further confirming a role for *jeb* as a dominant *dNf1* size defect suppressor, the *jeb^weli^* loss-of-function allele [42] dominantly increased *dNf1* pupal size (Figure 5B)

Previously, heterozygous mutations affecting RAF/MEK/ERK kinase cascade components *Draf* (*pole hole*; *phl*), *Dsor1/dMEK, or ERK/rolled (rl)*, did not modify *dNf1* size [5]. In agreement, two *phl*-uncovering deficiencies, *Df(1)ED6574* and *Df(1)ED11354*, did not score as modifiers (Table S2). No *rl* uncovering deficiencies were analyzed, but *Df(1)Exel9049*, which is among the stronger suppressors identified, deletes *Dsor1* and only two other genes, the neurogenic gene *almondex (amx)*, and *CG17754*, predicting a BTB and Kelch domain protein. Arguing that reduced Ras/ERK signaling upon loss of *Dsor1* combined with abnormal neuronal differentiation due to loss of *amx* may synergistically cause the observed strong effect, *Ras2-Gal4* driven UAS-RNAi transgenes targeting either gene, while causing pupal lethality at 25°C, increased *dNf1* pupal size at lower temperatures (Figure 5C). Moreover, suppression of the *dNf1* pupal size defect was also observed upon individual heterozygous loss of either *Dsor1* or *amx*, although at least with the tested alleles, combined loss of both genes did not have a more pronounced effect (Figure 5B). Previously, we did not observe suppression of the *dNf1^E2^* pupal size defect in crosses with the *Dsor1^S-1221^* allele [5]. A potential explanation may be that *Dsor1^LH110^* is a null mutant [43], whereas the molecular nature of *Dsor1^S-1221^* is undetermined. Genetic background differences between these *Dsor1* alleles are another potential explanation for the discrepant results.

Multiple screens aimed at identifying genes involved in Drosophila tyrosine kinase/Ras signaling have been performed [44]–[52]. Among the genes identified, several are uncovered by 1^st^ and 2^nd^ chromosome deficiencies that do not modify *dNf1* size. Suppressing *Df(2R)BSC161* uncovers 27 genes including c*onnector enhancer of KSR* (*cnk*), a scaffold protein that functions as a bimodal (both positive and negative) regulator of RAS/MAPK signaling [53], [54]. Supporting a role for *cnk* as a *dNf1* modifier, the *cnk^XE-385^* and *cnk^E-2083^* alleles acted as dominant suppressors (Figure 5B), and suppression was also observed upon RNAi-mediated Cnk knockdown using *Ras2-Gal4* or *P(GawB)C23-Gal4* neuronal drivers (Figure 5C). However, *Df(2R)BSC154*, which uncovers *cnk* and only nine other genes, did not score as a modifier (Table S2).

### *dNf1* Size Modifiers Involved in cAMP/PKA Signaling

The *dNf1* growth defect is suppressed by heat shock-induced expression of a constitutively active murine PKA catalytic subunit transgene, called PKA* [4], or by loss of the *dunce* (*dnc*) cAMP phosphodiesterase [35]. Further validating our screen, two *dnc* uncovering deficiencies and another that removes the region immediately upstream of the *dnc* coding region, all scored as suppressors (Table 2). Moreover, the *Pka-R2* gene, encoding a cAMP binding regulatory PKA subunit, whose dissociation from the catalytic subunit activates the latter, is uncovered by two additional suppressing deficiencies, whereas a deficiency that uncovers the major *Pka-C1* catalytic subunit gene scored as an enhancer (Table 2). *Df(1)ED7261*, which uncovers the *rutabaga* (*rut*) adenylyl cyclase, did not score as a modifier (not shown). Confirmation of *dnc* and *Pka-C1* as the genes responsible for the observed effects was obtained in crosses with three *dnc* and three *Pka-C1* loss-of-function alleles (Table 2). *Pka-R2* remains an attractive candidate suppressor, but expression *Pka-R2^RNAi^* transgenes in neurons had no effect and its role as a *dNf1* modifier remains unconfirmed (results not shown).

### Novel *dNf1* Modifiers

**Figure 6:**
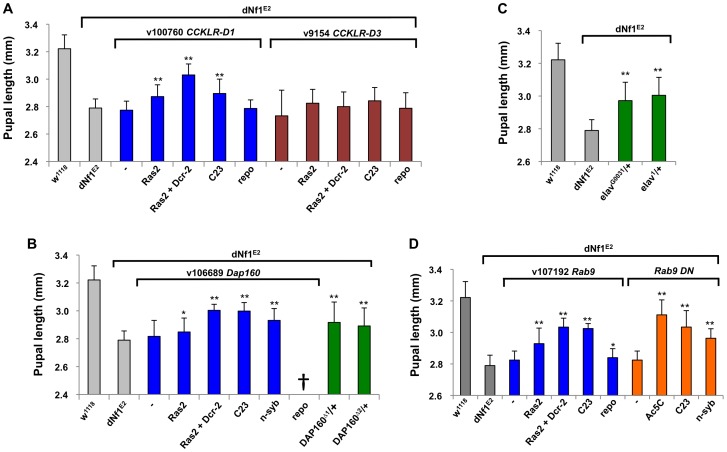
Validation of *dNf1* modifiers with neuronal functions. (A) *Ras2-Gal4* or *C23-Gal4* driven neuronal RNAi knockdown of *CCKLR-17D1* but not *CCKLR-17D3* suppressed the *dNf1* pupal size defect. (B) Identification of dynamin-associated protein 160 (Dap160) as a suppressor of *dNf1* growth. Neuronal RNAi targeting of *Dap160* increased *dNf1* pupal size as did two *Dap160* loss-of-function alleles. (C) Two *elav* alleles dominantly suppress the *dNf1* size defect. (D) Neuronal expression of a Rab9 RNAi transgene or of a dominant negative Rab9 mutant suppresses the *dNf1* size defect.

Recently, the cAMP-coupled CCKLR-17D1 drosulfakinin receptor, but not its closely related CCKLR-17D3 paralog, was identified as a positive regulator of synaptic growth [36]. The *CCKLR-17D1* gene is uncovered by three suppressing deficiencies, including *Df(1)Exel9051*, which uncovers only three other genes. The closely linked *CCKLR-17D3* paralog is not uncovered by *Df(1)Exel9051*, and while *Ras2-Gal4* or *P(GawB)C23-Gal4* driven neuronal *CCKLR-17D1* RNAi expression strongly suppressed the *dNf1* pupal size defect, similar suppression of *CCKLR-17D3* had no effect (Figure 6A).

Beyond *CCKLR-17D1*, several *dNf1* size modifiers are expressed in brain and/or have neuronal functions. Among these, dynamin-associated protein 160 (Dap160) is an intersectin-related scaffold implicated in synaptic vesicle exocytosis and neuroblast proliferation [55]–[58]. *Dap160* is uncovered by suppressing deficiencies *Df(2L)Exel6047* and *Df(2L)BSC302*, whose region of overlap encompasses ten genes. We note that *Df(2L)Exel6047* also uncovers the Drosophila *Ret* tyrosine kinase gene, the human ortholog of which is the receptor for glial-derived neurotrophic factor. *Ret* initially appeared an especially attractive candidate suppressor, because activating *RET* and inactivating *NF1* mutations can both lead to human pheochromocytoma [59], and because Drosophila *Ret* is expressed in larval brain neurons that resemble neuroendocrine cells [60]. However, among multiple lines of evidence that argue against a role for *Ret* in the *dNf1* growth defect, *UAS-dNf1* re-expression directed by a newly generated *Ret-Gal4* driver that recapitulates the endogenous larval brain Ret expression pattern (Figure S4B), or RNAi-mediated Ret inhibition, did not modify *dNf1* pupal size, nor did expression of a *UAS-Ret* K805A kinase dead transgene. Moreover, *Ret-Gal4* driven expression of *UAS*-*Ret* transgenes carrying the activating C695R mutation, which mimics a mutation found in multiple endocrine neoplasia type 2 did not phenocopy the *dNf1* reduced growth phenotype, although the same transgene did produce the previously described rough eye phenotype when driven by *GMR*-*Gal4* [60]; Figure S4C]. Further arguing against a role in *dNf1* growth control, *Ret* is uncovered by non-modifying *Df(2L)BSC312*. By contrast, *Dap160* loss-of-function alleles (*Dap160^Δ1^ and Dap160^Δ^* ^2^; [56]), or *Dap160* RNAi expression driven by three neuronal Gal4 drivers, suppressed the *dNf1* pupal size defect, identifying it as the responsible modifier (Figure 6B).

 The gene for the neuronal RNA binding protein elav is uncovered by suppressing *Df(1)Exel6221* and *Df(1)ED6396* whose region of overlap includes just three other genes. Identifying *elav* as the responsible modifier, *elav^1^* and *elav^G0031^* alleles strongly suppressed (Figure 6C). *Rab9* is a modifier uncovered by suppressing deficiency *Df(2L)Exel8041*. Neuronal but not glial *Rab9^RNAi^* expression increases *dNf1* pupal size, and the same result is seen upon neuronal expression of a Rab9 dominant negative [61] mutant (Figure 6D).

**Figure 7:**
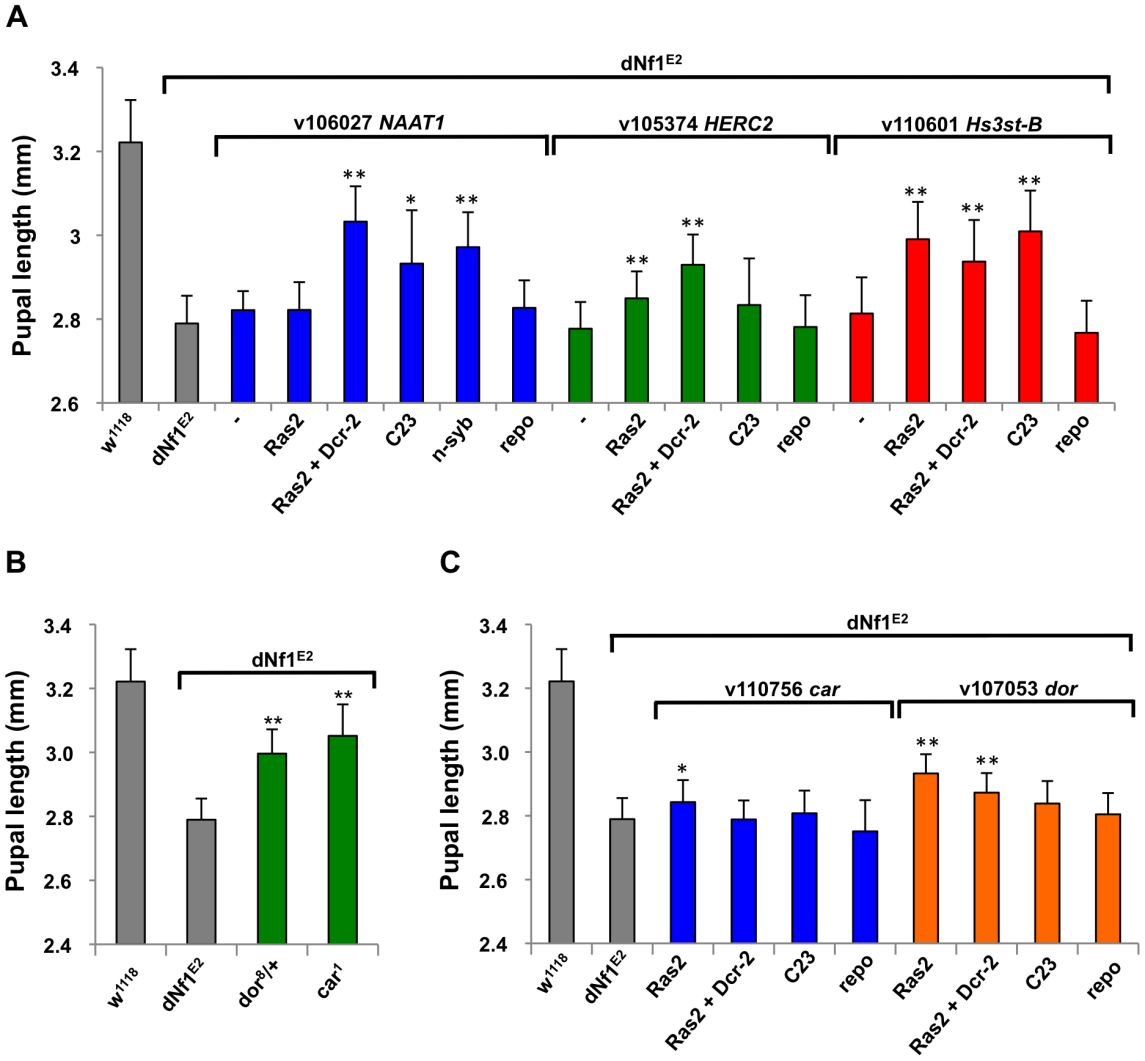
Identification of modifying genes with undetermined roles in *dNf1* suppression. (A) Validation of *NAAT1*, *HERC2* and *Hs3st-B* as *dNf1* modifiers. All three genes were identified by systematic RNAi screening of genes uncovered by suppressing deficiencies. (B) Loss-of-function alleles of Class C Vacuolar Protein Sorting complex subunits *carnation* (*car/Vps33A*) and *deep-orange* (*dor/Vps18*) increase *dNf1* pupal size. C) RNAi-mediated neuronal *car* or *dor* knockdown was not particularly effective, suggesting these genes may function elsewhere to modify *dNf1*-dependent growth.

*NAAT1*, coding for a larval gut and brain expressed amino acid transporter with a unique affinity for D-amino acids [38], is uncovered by suppressing *Df(1)Exel6290* and *Df(1)BSC533* whose region of overlap includes only four other genes. Identifying *NAAT1* as the responsible suppressor, three neuronal Gal4 lines driving the expression of three *NAAT1* targeting RNAi transgenes suppressed the *dNf1* size defect, whereas *Repo-Gal4* driven glial expression had no effect (Figure 7A and Table 2).

Mammalian E3 ubiquitin ligase HERC2 controls the ubiquitin-dependent assembly of DNA repair proteins on damaged chromosomes [62]. Drosophila *HERC2* is uncovered by suppressing deficiency *Df(1)Exel6254*, which also uncovers the *syx16*, coding for syntaxin 16. No *HERC2* alleles exist, but *Ras2*-*Gal4* driven expression of a *UAS-HERC2^RNAi^* transgene (*v105374*) strongly suppressed the *dNf1* pupal size defect (Figure 7A), whereas similar knockdown of *Syx16* had no statistically significant effect (not shown). The gene for another E3 ligase component, *Cul-3*, is uncovered by three enhancing deficiencies, and a *Cul-3* loss-of-function allele or *Ras2-Gal4* driven expression of a *Cul-3* RNAi transgene both enhanced the *dNf1* size defect, identifying it as the responsible gene (Table 2).

Suppressing *Df(1)Exel9068* uncovers only four genes, including one encoding the TORC2 complex subunit Rictor. However, systematic *Ras2-Gal4* driven RNAi knockdown of *Df(1)Exel9068* uncovered genes identified *Hs3st-B*, encoding one of two Drosophila heparan sulfate 3-O sulfotransferases, as a potent *dNf1* size defect suppressor (Figure 7A), whereas knockdown of Rictor had no effect (not shown). Others previously identified *Hs3st-B* as a positive regulator of Notch signaling [39]. However, the heparan sulfate proteoglycan substrates of *Hs3st-B* bind various growth factors and other ligands and have been implicated in a variety of biological processes. Exactly why loss of *Hs3st-B* suppresses the *dNf1* growth defect remains to be determined.

Two functionally related *dNf1* growth defect suppressors *carnation* (*car/Vps33A*) and *deep-orange* (*dor/Vps18*), encode subunits of the Class C Vacuolar Protein Sorting (VPS) complex, required for the delivery of endosomal vesicles to lysosomes [63]; Figure 7B]. The *Vps16A* gene encodes a third member of this complex [64], but whether *Vps16A* located on the 3^rd^ chromosome also acts as a *dNf1* suppressor, or whether pharmacological inhibition of lysosomal degradation affects *dNf1* pupal size are questions that remain to be answered.

B4/Susi is a coiled-coil protein without obvious orthologs outside of insects. It functions as a negative regulator of Drosophila class I phosphatidylinositol-3 kinase Pi3K92E/Dp110 by binding to its Pi3K21B/dP60 regulatory subunit. Homozygous *B4* mutants have an increased body size [65], which may explain why *Ras2-Gal4*-driven RNAi-mediated suppression of *B4*, uncovered by suppressing deficiency *Df(2L)BSC147*, increased *dNf1* pupal size (not shown). However, whether *B4* is the responsible dominant modifier is doubtful, given that it is also uncovered by *Df(2L)BSC692*, a non-modifying deficiency. Moreover, we previously found that heterozygous loss of *Pi3K21B*, or neuronal expression of a dominant negative *Pi3K92E* transgene, did not modify *dNf1* pupal size [5]. Beyond *B4*, *dNf1* size modifying deficiencies uncovered no genes involved in the canonical growth regulating pathways mediated by insulin and ecdysone. Indeed, several such genes were uncovered by non-modifying deficiencies. Among these genes, fat body expressed insulin-like growth factor *Ilp6*, which regulates larval growth in the post-feeding phase [66], [67], is uncovered by two non-modifying deficiencies. A single non-modifying deficiency, *Df(2L)BSC206*, uncovers both the *chico* and *pten* genes, whose products antagonistically control insulin-stimulated Pi3K92E/Dp110 activity, leading to changes in body, organ, and cell size [68], [69]. Among subunits of the cell growth regulating mTORC1 complex, *raptor* is uncovered by three and *Tor* by one non-modifying deficiency. Among genes implicated in ecdysone signaling, the ecdysone co-receptor *ultraspiracle* and the ecdysone-induced growth regulating *DHR4* nuclear receptor [70] are each uncovered by non-modifying deficiencies, and two such deficiencies uncover *Ptth*, coding for prothoracicotropic hormone, which provides developmental timing cues by stimulating the production of ecdysone [71], [72]. These results reinforce our conclusion that the canonical growth regulating pathways involving insulin and ecdysone play no obvious roles in *dNf1* growth control.

### Manipulating cAMP/PKA Signaling in the Ring Gland Affects *dNf1* Systemic Growth Non-Cell-Autonomously

**Figure 8:**
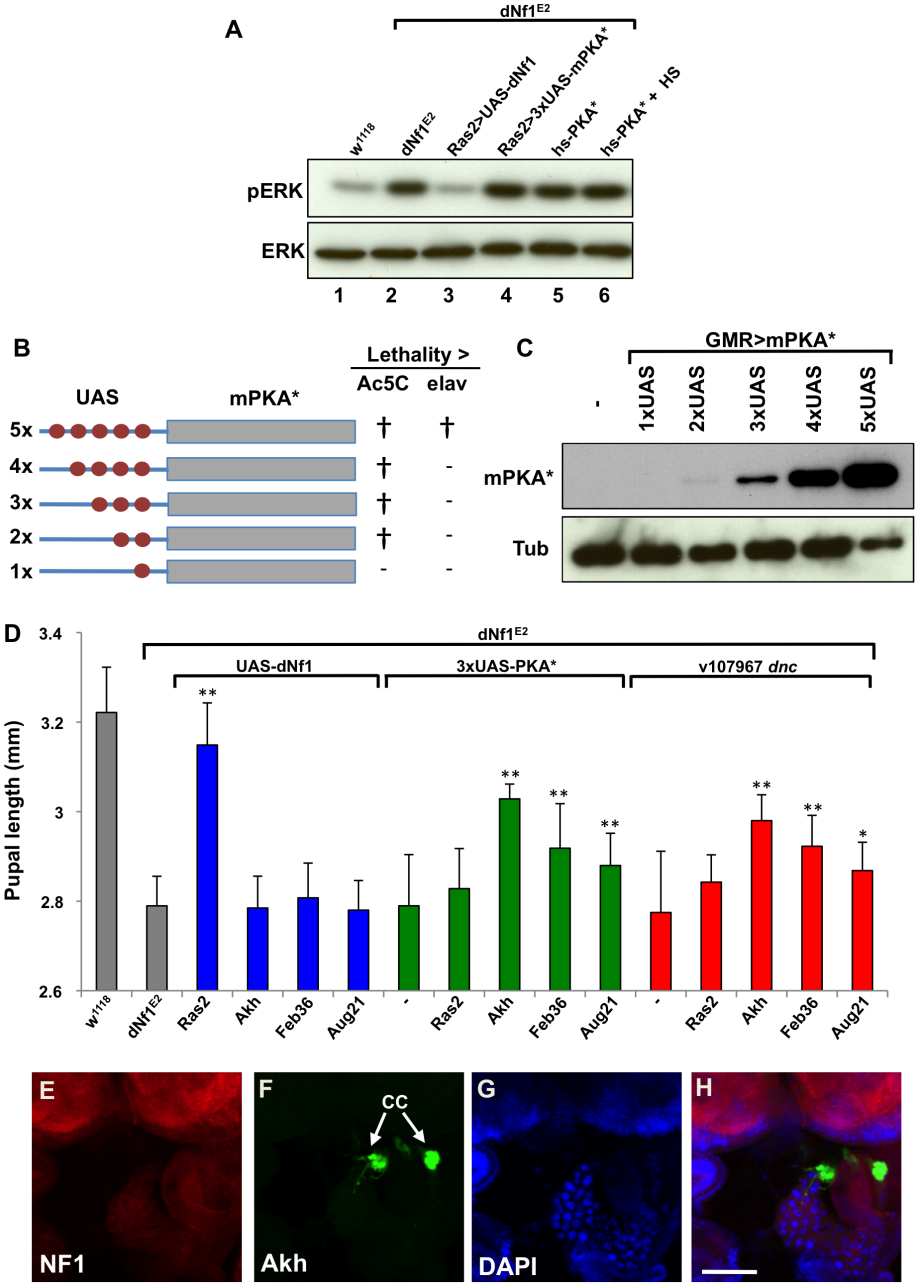
*dNf1* systemic growth related RAS/ERK and cAMP/PKA signals appear functionally and topographically distinct. (A) The elevated larval CNS pERK level of *dNf1* mutants is reduced by neuronal expression of *dNf1*, but not by neuronal or heat-shock induced ubiquitous expression of PKA*. Western blot of pERK levels in larval CNS of the indicated genotypes. In lane 6, larvae received a daily 20 min 37°C heat shock throughout development, a protocol that suppresses the *dNf1* growth defect [4]. (B) Structure of UAS-PKA* transgenes with 1 to 5 UAS elements. The lethality of these transgenes when driven with either *Ac5C-Gal4* or *elav-Gal4* is indicated by † whereas (−) indicates viable offspring. (C) Western blot of adult head lysates showing relative expression of *GMR-Gal4*-driven transgenic PKA*. Tubulin is used as a loading control. (D) Expression of PKA* or knockdown of *dnc* by shRNAi in the ring gland rescues the *dNf1* pupal size defect. In contrast, *UAS-dNf1* expression with the same ring gland drivers fails to restore systemic growth. (E–H) Expression pattern of *Akh-Gal4* driving *UAS-GFP*, co-stained with DAPI and anti-dNF1. GFP expression in the corpora cardiaca (CC) is indicated. Scale bar = 50 µm. As previously noted [74], anti-dNf1 staining is strong in the CNS, whereas staining in the ring gland is close to background.

Several results argue that defects in Ras/ERK and cAMP/PKA signaling responsible for the *dNf1* growth defect involve non-overlapping cell populations. Firstly, heat shock-induced *hsp70*-PKA*, or *Ras2*-*Gal4* induced attenuated UAS-PKA* transgene (see below) expression rescued the *dNf1* pupal size defect, but failed to reduce the elevated larval brain phospho-ERK level (Figure 8A). Moreover, several neuronal RNAi drivers that increase *dNf1* pupal size when driving *UAS-dNf1* [5], failed to modify this phenotype when driving *dnc^RNAi^* transgenes, even in the presence of the *UAS-Dcr-2* RNAi enhancer (Table 3). This prompted us to investigate whether genetic manipulation of cAMP/PKA signaling in cells other than *dNf1* requiring neurons was more effective.

**Table 3:** Restoration of systemic growth by *dNf1* and cAMP/PKA involves different tissues.

Gal4	UAS-*dNf1*	*dnc v107967*	2×UAS-PKA*	3×UAS-PKA*	4×UAS-PKA*	5×UAS-PKA*
*Act5C*	Rescue	Rescue (pupal †)	SV	†	†	†
*elav*	Rescue	NR	NR	NR	NR	†
*elav+Dcr-2*	Rescue	NR	n/a	n/a	n/a	n/a
*Ras2(41)*	Rescue	NR	NR	NR	†	†
*Ras2(41)+Dcr-2*	Rescue	NR	n/a	n/a	n/a	n/a
*C23*	Rescue	NR	NR	NR	NR (pupal †)	†
*Feb36*	NR	Rescue	NR	Rescue	†	†
*Aug21*	NR	Rescue	Rescue	Rescue	Rescue	†
*Akh*	NR	Rescue	Rescue	Rescue	Rescue	Rescue (SV)

*Act5C*-*Gal4* driven ubiquitous *dNf1* re-expression, or *elav*-*Gal4* and *Ras2*-*Gal4* driven neuronal re-expression rescues the *dNf1* pupal size defect, whereas *dnc* RNAi or UAS-PKA* expression controlled by the same drivers is ineffective. By contrast, expressing *dNf1* in specific parts of the neuroendocrine ring gland with the *Akh*-*Gal4*, Feb36-*Gal4* or Aug21-*Gal4* drivers fails to rescue, whereas using the same drivers to express *dnc* RNAi or attenuated UAS-PKA* transgenes does increase *dNf1* pupal size. All crosses produced viable adults unless otherwise indicated.

† denotes lethality, SV sub-viable, n/a not applicable, NR non-rescue.

The data shown summarize results of a larger effort to identify the tissues in which *dNf1* and cAMP/PKA affect systemic growth. Full results are shown in Table S5.

To manipulate cAMP/PKA signaling tissue-specifically we used three *UAS-dnc^RNAi^* transgenes. We also generated a series of attenuated UAS-PKA* transgenes using vectors with modified Gal4-inducible promoters harboring just 2, 3 or 4 Gal4-binding UAS elements (Figure 8B and C). We made the latter transgenes because a UAS-PKA* expression using the five UAS element containing standard UAS-T vector is lethal in combination with most Gal4 drivers [73]. As reported previously [74], driving *UAS-dNf1* ubiquitously with *Act5C*-*Gal4*, or broadly in neurons with *elav*-*Gal4*, *Ras2*-*Gal4*, *c23*-*Gal4*, or *386Y*-*Gal4* restored *dNf1* pupal size, whereas driving the same transgene with more restricted neuronal or non-neuronal drivers had no effect (Figure 8D and Table 3). By contrast, driving the expression of *UAS-dnc^RNAi^* or attenuated UAS-PKA* transgenes with the same set of broadly expressed neuronal drivers was ineffective (Tables 3 and S5). We note that expression of the 2×UAS-PKA* and 3×-UAS-PKA* transgenes was generally well tolerated, whereas the 4×UAS-PKA* and the 5×UAS-PKA* transgenes exhibited increasing levels of lethality (Tables 3 and S5). Arguing that rescue of the *dNf1* growth defect by manipulating cAMP/PKA signaling or *dNf1* expression involves different cells, strong pupal size rescue was observed by increasing cAMP/PKA signaling in adipokinetic hormone-producing cells at the base of the neuroendocrine ring gland using the *Akh*-*Gal4* driver (Figure 8D). Rescue was also observed with the *Feb36-Gal4* and *Aug21-Gal4* ring gland drivers (Figure 8D), which give rise to expression in the corpora allata, the source of juvenile hormone, but not with the P0206-*Gal4* or Mai60-*Gal4* drivers, which express predominantly in the prothoracic gland (Table 3). The tissue specificity of all Gal4 drivers used in this and other experiments was verified by microscopic observation of dissected *UAS-GFP* expressing larvae (Table S4 and Figures 8E–H and S5).

### *dAlk*, *Jeb*, *Cnk* and *CCKLR-17D1* Suppress a *dNf1* NMJ Architectural Defect

During larval development, significant expansion of the NMJ arbor must occur, reflecting the steady muscle growth that takes place during larval life. As the NMJ grows, additional branches and boutons are added to the initial synaptic arbor that forms during late embryonic stages upon motor axon contact with its target muscle. As a result, at the wandering third instar stage, wild-type NMJs contain a highly stereotyped, segment specific number of synaptic boutons [75]. Recently, it was reported that *dNf1* functions presynaptically to constrain NMJ synaptic growth and neurotransmission [16]. In *dNf1* null mutant wandering third instar larvae, while the distribution of major presynaptic proteins is unaffected, increased overall size and synaptic bouton number is apparent at multiple NMJs, supporting a specific role for *dNf1* in restricting NMJ expansion [16]. Several *dNf1* suppressors that emerged in the current screen have also been linked to synapse morphogenesis, including CCKLR-17D1, which functions as a promoter of NMJ growth [36]. As our screen identified *CCKLR-17D1* as a dominant *dNf1* size defect suppressor, we wanted to confirm the *dNf1* NMJ phenotype and test whether *CCKLR-17D1* and other suppressors affected this defect.

**Figure 9:**
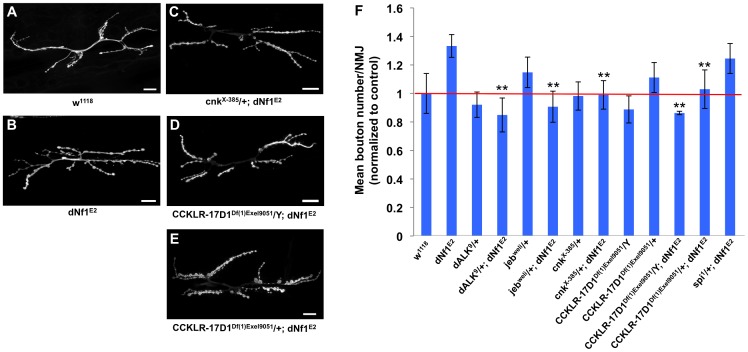
Several *dNf1* pupal size defect suppressors also suppress a NMJ synaptic overgrowth phenotype. (A–E) Representative micrographs of larval muscle 6/7 NMJs of the indicated genotypes. F: Mean bouton number per NMJ normalized to wild-type control. Compared to wild-type (*w^1118^*; A), *dNf1* mutants (*dNf1^E2^*; B) have an increased bouton number. While a *cnk* loss-of-function allele had no obvious NMJ phenotype, it dominantly suppressed the *dNf1* NMJ defect (C). Similarly, the *dNf1* NMJ phenotype was suppressed in *Df(1)Exel9051* males that lack CCKLR-17D1 (D), while females heterozygous for CCKLR-17D1 (E) showed a lower level of suppression. *Spitz (spi)* is uncovered by a modifying deficiency but does not affect *dNf1* size and was used as a negative control. In panels A–E, scale bars represent 5 µm. In panel F, error bars denote standard error of the mean.

By quantifying bouton number at the NMJ on muscles 6 and 7, we confirmed that *dNf1* mutants have a significant increase in mean bouton number (Figure 9A and B). In addition, this analysis confirmed previously published phenotypes for *dAlk*, *jeb* and *CCKLR-17D1* [36], [76]. Importantly, the *dNf1* synaptic overgrowth phenotype is dominantly suppressed by *CCKLR-17D1*, *dAlk*, *jeb*, and *cnk* alleles (Figure 9B), arguing that all four genes are epistatic to *dNf1.* As a control we analyzed an allele of *spitz* (*spi*), which encodes an EGF-like growth factor and is uncovered by suppressing *Df(2L)Exel8041*. However, *spi* shows no genetic interaction with *dNf1*, as loss of *spi* modified neither the pupal size nor the NMJ overgrowth phenotypes (Figure 9B and data not shown).

### Human ALK Is Expressed in Schwann Cells and May Serve as a Therapeutic Target in NF1

The identification of *dAlk* as a suppressor of all hitherto analyzed *dNf1* defects prompted us to explore whether human ALK represents a therapeutic target in NF1. Given our hypothesis that NF1 negatively regulates ALK stimulated Ras/ERK signaling, in order to play such a role, ALK and NF1 must be co-expressed in cells that give rise to symptoms. We previously found that *dNf1* and *dAlk* expression overlaps extensively in Drosophila larval and adult CNS [15], and the expression of orthologs of both genes also overlaps in the murine CNS [77], [78]. While overlapping CNS expression is compatible with a role for ALK in NF1-associated cognitive dysfunction, a causative role in another hallmark NF1 symptom, peripheral nerve-associated tumors, is less obvious. Among the near universal symptoms on NF1, benign neurofibromas consist of Schwann cells, perineurial fibroblasts, infiltrating mast cells, and nerve elements, with the Schwann cells sustaining the second *NF1* hit [79]. To test whether increased ALK signaling in the absence of NF1 might play a role in the development of neurofibromas, we used reverse transcription/PCR to detect the presence or absence of *ALK* mRNA in neurofibroma-derived *NF1^−/−^* Schwann cells and *NF1^+/−^* fibroblasts, using RNAs kindly provided by Drs. Eric Legius and Eline Beert. In these experiments, two different primer sets readily detected ALK mRNA in *NF1^−/−^* Schwann cells, but not in *NF1^+/−^* fibroblasts derived from the same tumors (Figure S6).

**Figure 10:**
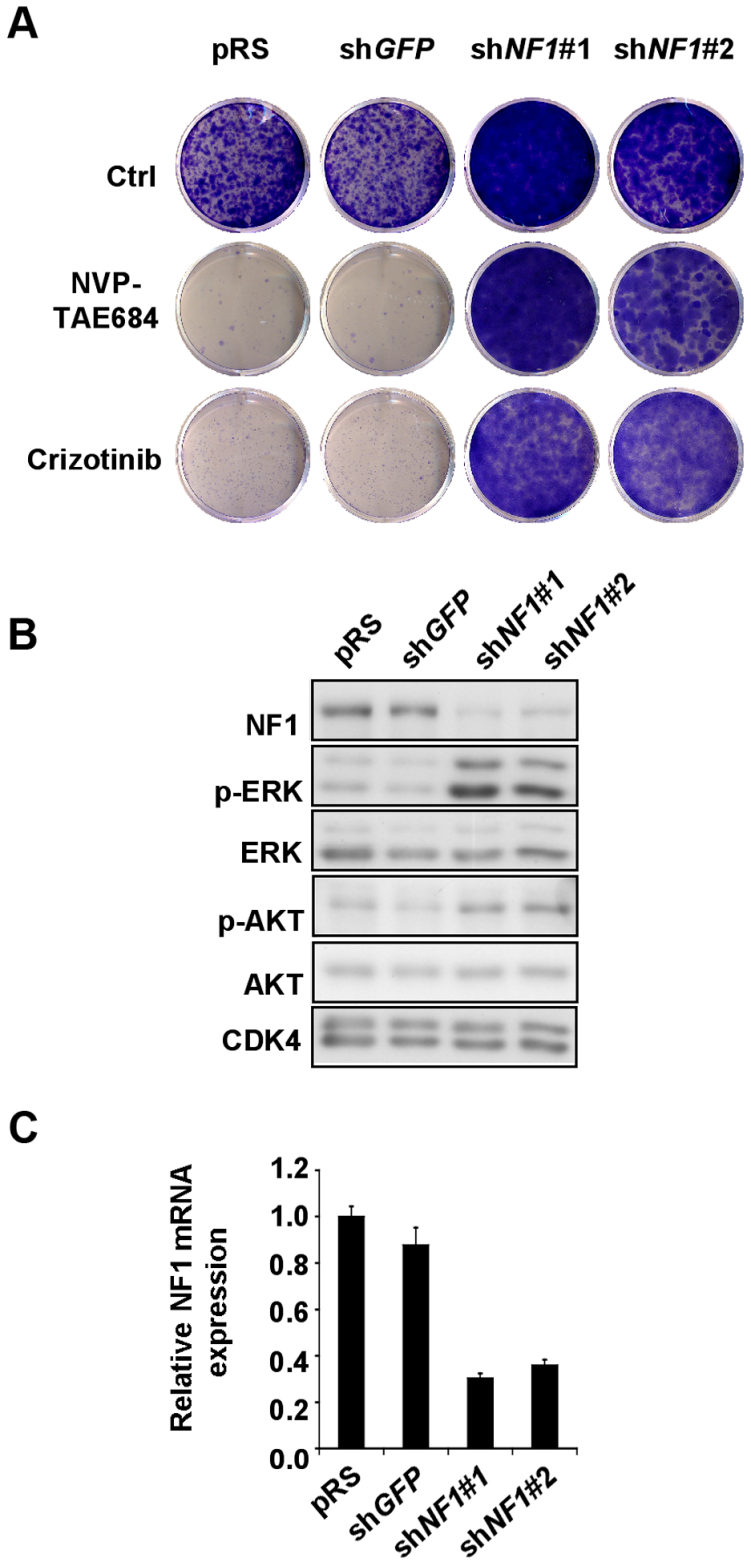
*NF1* suppression leads to ERK activation and confers resistance to ALK inhibitors in human neuroblastoma cells. (A) *NF1* knockdown confers resistance to ALK inhibitors in human neuroblastoma cells. SH-SY5Y cells expressing pRS and sh*GFP* control vectors, or sh*NF1* vectors were grown in the absence or presence 50 nM NVP-TAE684 or 250 nM crizotinib. The cells were fixed, stained and photographed after 14 (untreated and crizotinib treated), or 21 (NVP-TAE684 treated) days. (B) Down-regulation of *NF1* results in elevated level of phosphorylated p-ERK and p-AKT. Western blot analysis of total lysates of SH-SY5Y cells expressing pRS, sh*GFP* or sh*NF1* vectors. (C) The level of *NF1* knockdown by each of the RNAi vectors was measured by examining the *NF1* mRNA levels by qRT-PCR. Error bars denote standard deviation.

 To test whether functional interactions between NF1 and ALK exist in human cells, we used the SK-SY5Y and Kelly neuroblastoma cells, both of which harbor constitutively active F1174L *ALK* alleles, and both of which are highly sensitive to pharmacological ALK inhibition [80]. Compatible with a role for NF1 as a negative regulator of mitogenic ALK/RAS signals, qRT-PCR verified *NF1* knockdown with two shRNA retroviral vectors increased the resistance of both lines to ALK inhibitors NVP-TAE684 and Crizotinib (Figures 10A, 10C and S7). Compatible with a model in which NF1 negatively regulates ALK/RAS signaling, *NF1* knockdown resulted in elevated ERK and AKT activation (Figures 10B). Moreover, expression of activated *KRAS*, *BRAF*, or *MEK* transgenes, but not of other Ras effector transgenes, in SH-SY5Y cells conferred similar resistance to ALK inhibition (Figure S8).

## Discussion

The work reported here was motivated by the fact that human NF1 is a characteristically variable disease, the severity of which is controlled at least in part by symptom-specific modifier genes [81]. Thus, a genetic analysis in Drosophila might not only reveal molecular pathways controlled by the highly conserved (50% identical) dNf1 protein, but also provide clues to the identity of human modifiers, which by virtue of their rate-limiting roles in symptom development might serve as therapeutic targets. The current work was also motivated by the fact that, for reasons that remain poorly understood, most *dNf1* null mutant phenotypes are rescued by increasing, or phenocopied by decreasing, cAMP/PKA signaling. The identification of genetic modifiers of a cAMP/PKA sensitive defect might reveal how loss of dNf1 affects cAMP/PKA signaling, and help to resolve the long-standing controversy as to whether dNf1 affects cAMP/PKA signaling directly, independent of its role as a Ras regulator [10], [27], or indirectly, secondary to a Ras signaling defect [5], [15].

While recognizing that none of the thus far identified *dNf1* phenotypes are ideally suited for use in modifier screens, we selected the pupal size defect as the phenotype to analyze in our screen for three main reasons. First, pupariation occurs at the end of the larval growth period, and pupal size is readily assessed by inspecting pupae attached to the side of culture vials, making this phenotype amenable to a large-scale screen. Second, the growth defect is among several cAMP/PKA sensitive *dNf1* phenotypes. Finally, reduced growth is also a symptom of human NF1 and other RASopathies [1], [82]. However, while compelling reasons support the selection of this phenotype, confounding factors include that Drosophila size is a sexually dimorphic phenotype affected by population density, feeding, environmental conditions such as temperature, and genetic background differences. Moreover, while heterozygous *dNf1* mutants are marginally smaller than wild-type pupae [5], the more robust size phenotype (∼15% reduction in linear dimensions, ∼25% reduction in weight) used in our screen is only observed upon homozygous loss of *dNf1*. Thus, our screen was not designed to find modifiers that act on the dNf1 protein itself, like the recently identified SPRED proteins [83]. Finally, organism size is a function of growth rate and duration, both of which are regulated by hormonal cascades that involve cross-talk between the larval brain, the neuroendocrine ring gland, the fat body and other tissues [19], [84]. Thus, a screen for modifiers of *dNf1*-regulated growth may uncover genes involved in various aspects of systemic growth control.

Early attempts to identify *dNf1* pupal size modifiers were abandoned when >95% of large X-ray induced 2^nd^ chromosome deficiencies were found to be lethal in a *dNf1* background (Glenn Cowley, Iswar Hariharan and A.B., unpublished), or when a pilot chemical mutagenesis screen found the reliable mapping of identified enhancer or suppressor mutations to be impracticable (Suzanne Brill, Iswar Hariharan and A.B., unpublished). Both aborted screens informed the current effort, which used precisely defined small deficiencies, isogenic crossing schemes and experimental protocols that guarded against population density differences. In total we analyzed 486 1^st^ and 2^nd^ chromosome deficiencies that together uncover well over 80% of chromosome 1, 2L and 2R genes (Table 1). Among the screened deficiencies, 132 (27.2%) significantly modified *dNf1* pupal size (*p*<0.01; two-tailed Student's *t*-test). While this is a large number, 20 deficiencies were subsequently eliminated because they also affect wild-type size. Several modifying deficiencies also uncover overlapping genomic segments, further reducing the number of *dNf1* modifying loci to 76. During follow-up studies aimed at identifying responsible genes, we prioritized genes uncovered by suppressing deficiencies over those uncovered by enhancing ones, modifiers uncovered by overlapping deficiencies over those uncovered by single deletions, modifiers uncovered by small deficiencies over those uncovered by larger ones and stronger modifiers over weaker ones. We also limited ourselves to genes that function in the nervous system, based on the consideration that *dNf1* re-expression in larval neurons is sufficient to suppress the growth defect [5].

We previously reported that *dNf1* growth and learning defects are phenocopied by increasing neuronal Jeb/dAlk/ERK signaling, and suppressed by genetic or pharmacological attenuation of this pathway [15]. Validating our screen, deficiencies that uncover *jeb* and *dAlk* were identified as dominant *dNf1* size defect suppressors. Others recently reported that Jeb/dAlk signaling allows brain growth to be spared at the expense of other tissues in nutrient restricted Drosophila and identified a glial cell niche around neuroblasts as the source of Jeb under these conditions [41]. However, Jeb involved in systemic growth appears of mainly neuronal origin, as RNAi-mediated *jeb* knockdown in neurons increased *dNf1* pupal size, whereas only one of four tested glial drivers produced partial rescue (Figure 5A).

The identification of cAMP/PKA pathway modifiers *dnc*, *PKA-C1* and tentatively *PKA-R2* further validates our screen. Arguing that increased PKA activity doesn't suppress *dNf1* defects by attenuating Ras/Raf/MEK/ERK signaling, *hsp70*-PKA* transgene expression, using a daily heat shock regimen that suppresses the *dNf1* size defect [4], does not reduce the elevated *dNf1* larval brain phospho-ERK level, and neither does *Ras2*-*Gal4* driven neuronal UAS-PKA* expression (Figure 8D). Providing further mechanistic clues, our results demonstrate that *dNf1* and cAMP/PKA both affect systemic growth non-cell-autonomously, but not necessarily in the same cells. Thus, we previously showed that only relatively broadly expressed neuronal Gal4 drivers restored mutant growth when driving *UAS-dNf1*, whereas multiple drivers expressed in specific subsets of neurons, including several expressed in the ring gland, lacked the ability to restore *dNf1* growth [5]. By contrast, using *UAS*-*dnc^RNAi^* or a series of newly generated attenuated UAS-PKA* transgenes that avoid the toxicity associated with high level PKA expression [73], we now show that manipulating cAMP/PKA signaling with broadly expressed neuronal Gal drivers does not affect the *dNf1* size phenotype, whereas the same transgenes induced with three ring gland drivers did suppress. Intriguingly, the most potent rescue was observed when *UAS-*d*nc^RNAi^* or attenuated UAS-PKA* transgenes were driven in AKH-producing cells at the base of the ring gland, whereas weaker rescue was also observed with two ring gland drivers that show overlapping expression in the juvenile hormone producing corpora allata. This suggests that the *dNf1* growth deficiency involves a defect in processes controlled by one or both of these neuroendocrine hormones.

As might be expected of a screen that used systemic growth as a read-out, our work identified a diverse set of potential modifiers. Notably, however, among a non-exhaustive set of 18 1^st^ or 2^nd^ chromosome genes implicated in various aspects of Drosophila body, organ, and/or cell size control (*dAlk, B4, chico, hpo, Hr4, Ilp6, jeb, Mer, mir-8, Pi3K21B, Pten, Ptth, SNF1A, sNPF, step, Tor*, *ush and yki*; see Table S3 for details), only *dAlk* and *jeb* scored as dominant *dNf1* pupal size modifiers, whereas the remaining 16 genes were uncovered by non-modifying deficiencies, or in the case of *Ptth*, by two deficiencies that altered developmental timing (Table S2). Further explaining this lack of overlap, the previously implicated PI3 kinase regulator *B4* act in a recessive manner and several of the above listed genes function outside of the CNS. Our screen excluded such genes, because *dNf1* controls growth non-cell-autonomously by regulating neuronal Ras [5]. As previously noted, a special case is provided by insulin pathway components *chico* and *Pten*, which affect growth antagonistically. Both genes map within 5 kb of each other on the 2^nd^ chromosome and are uncovered by the same non-modifying deficiency.

Two newly identified *dNf1* growth defect suppressors, *Dap160* and *CCKLR-17D1*, affect synaptic architecture or functioning [36], [56], [57]. Because *dNf1* was recently reported to function downstream of focal adhesion kinase to restrain NMJ synaptic growth and neurotransmission [16], and because the cholecystokinin receptor related *CCKLR-17D1* drosulfakinin receptor stimulates NMJ growth [36], we analyzed whether this and three Ras signaling related *dNf1* size defect suppressors also affected NMJ architecture. Our results confirm that *dNf1* mutants exhibit synaptic overgrowth, and show that loss of *CCKLR-17D1* suppresses this defect. Importantly, loss of *jeb*, *dAlk*, or *cnk* similarly suppresses both size and synaptic overgrowth defects, suggesting that both phenotypes may be related.

The results presented here further support our previous conclusion that excess neuronal Jeb/dAlk/Ras/MEK/ERK signaling is the root cause of the cAMP/PKA sensitive *dNf1* systemic growth defect. What happens downstream of this primary defect remains less clear, although our demonstration that increasing cAMP/PKA signaling in AKH-producing cells and other parts of the neuroendocrine ring gland suppresses the size defect provides an important new clue, not only about pathways involved in the *dNf1* growth defect, but also about the likely non-cell-autonomous cause of similar growth defects of *PKA-C1* or *dCreb2* mutants [85], [86]. Other questions that remain to be fully answered concern the role of the NMJ architectural defect in the *dNf1* growth deficiency and the role of Jeb/dAlk signaling in the NMJ defect. We note in this respect that that *C. elegans* ALK ortholog, *T10H9.2*, has been implicated in synapse formation [87], and that recent work suggests a role for trans-synaptic Jeb/dAlk signaling in the control of neurotransmission and synaptic morphology [88]. However, while the *dNf1* growth defect is due to excess dAlk signaling in neurons, NMJ synapse formation has been suggested to involve the release of presynaptic Jeb activating postsynaptic dAlk [88]. Further work will have to establish whether the suppression of the *dNf1* NMJ overgrowth phenotype by *jeb*, *dAlk* and *cnk* involves cell autonomous roles for these genes at synapses, or non-cell-autonomous functions elsewhere in the CNS. Further work is also required to reveal the functional significance and the sites of action of other novel modifiers identified in our screen.

From a clinical perspective, perhaps the most relevant questions raised by our work are whether NF1 regulated ALK/RAS/ERK signaling is evolutionarily conserved and whether excessive ALK/RAS/ERK signaling contributes to human NF1 symptoms. Much indirect evidence hints at a positive answer to both questions. First, the expression of ALK and NF1 largely overlaps in the murine nervous system [77], [78], same as it does in Drosophila [15]. Second, ALK functions as an oncogene and NF1 as a tumor suppressor in neuroblastoma [89]–[94]. Third, midkine, a ligand that activates mammalian ALK [95], is produced by *NF1^−/−^* Schwann cells, present at elevated levels in NF1 patient skin and serum, and acts as a mitogen for NF1 tumor cell lines [96]–[98]. We add to this evidence by showing that shRNA-mediated *NF1* knockdown renders two oncogenic ALK-driven human neuroblastoma cell lines resistant to pharmacological ALK inhibition, and by confirming that *ALK* mRNA is expressed in neurofibroma-derived *NF1^−/−^* human Schwann cells. These findings make a strong case that ALK should be explored as a therapeutic target in NF1, and that loss of *NF1* expression should be considered as a potential mechanism in cases of acquired resistance to ALK inhibition [99].

## Materials and Methods

### Fly Stocks and Experiments

The *dNf1^E1^* and *dNf1^E2^* alleles have been described [5]. Exelixis, DrosDel and BSC deficiencies were obtained from the Bloomington Stock Center. Transgenic RNAi lines were obtained from the Vienna *Drosophila* Research Center (VDRC) and the TRiP Collection at Harvard Medical School. *Eaat1^SM1^* and *Eaat1^SM2^* were provided by D. van Meyel, *dALK^8^* and *jeb^weli^* by R. Palmer, *cnk^XE-385^* and *cnk^E-2083^* by M. Therrien, and *car^Δ146^* by H. Kramer, *ppl^06913^* by M. Pankratz, *hs-Ilp2* transgenic line by E. Rulifson and *UAS-Rab9 DN* by R. Hiesinger. Flies were maintained on agar-oatmeal-molasses medium at 25°C, unless otherwise indicated.

To assess feeding, larvae at various stages of development were placed on blue food dye-stained yeast paste, removed after 20 min, washed and photographed. To analyze wandering behavior, 100 larvae (age 40–44 hr after egg deposition (AED)) were placed on an agar plate with a central blob of yeast paste, and their position after 24 hr was documented. To assess the expression of starvation-sensitive genes, larvae at 72 h AED were placed in vials with water for 16 hr, after which RNA was prepared and subjected to blot analysis. To determine developmental timing, L1 larvae were collected 24 hr AED using a 2 hr egg collection and reared at 140 animals per vial. The number of larvae that pupariated was scored at hourly intervals. To determine the larval weight, L1 larvae were collected 24 hr AED using a 2 hr egg collection. Larvae were reared at 140 larvae per vial and groups of 10 larvae were weighed at 8 hr intervals. Longevity was assessed by maintaining adult flies under standard conditions and counting the number of dead flies at regular intervals. In each of these assays, genotypes were tested in duplicate. To induce *hs-Ilp2* transgene expression, culture vials were placed in a circulating water bath at 37°C for 10 min once or twice a day with an 8 hr interval.

### Insulin-Like Protein mRNA Quantification

The 7500 Fast Real-Time PCR System from Applied Biosystems was used to determine *Ilp* mRNA levels in RNA prepared from dissected larval brains or from whole wandering stage 3^rd^ instar larvae. Results were normalized to *RpL32*. The following primers were used: *IIp2*-Forward, GGCCAGCTCCACAGTGAAGT,*Ilp2*-Reverse, TCGCTGTCGGCACCGGGCAT, *Ilp3*-Forward, CCAGGCCACCATGAAGTTGT. *Ilp3*-Reverse, TTGAAGTTCACGGGGTCCAA, *Ilp5*-Forward, TCCGCCCAGGCCGCAAACTC, *Ilp5*-Reverse, TAATCGAATAGGCCCAAGGT, *Ilp6*-Forward, CGATGTATTTCCCAACAGTTTCG, *Ilp6*-Reverse, AAATCGGTTACGTTCTGCAAGTC, *Ilp7*-Forward, CAAAAAGAGGACGGGCAATG, *Ilp7*-Reverse, GCCATCAGGTTCCGTGGTT. Expression of the distantly related *Ilp8* and the midgut-expressed *Ilp4* genes [21] was not analyzed.

### Genetic Screening, Validation, and Statistical Analysis

The crossing schemes in Figure 2 were used to generate *dNf1^E2^* mutants carrying 1^st^ and 2^nd^ chromosome deficiencies. To avoid crowding, cultures were maintained at 100–200 pupae per culture vial. Initial scoring used calipers set at the length of *dNf1* female pupae, ignoring *dNf1* heterozygotes recognizable by the presence of the *TM6B* balancer. Next, the length of individual pupae carrying candidate modifying deficiencies was measured by determining their head-to-tail length using a microscope fitted with NIS-Elements AR 3.0 imaging software. Measured pupae were then placed in 96-well plates (Falcon) to determine their gender and, if necessary, the genotype of eclosed flies. At least 40 pupae were measured for each genotype, and only measurements of female pupae were used to calculate mean values and standard deviations. Statistical significance was assessed with a two-tailed Student's *t*-test. Throughout this report, single or double asterisks denote *p*-values<0.05 or <0.01 respectively.

To identify responsible modifiers we used specific alleles or *UAS-RNAi* knockdown. Alleles and *UAS-RNAi* lines on the 1^st^ and 2^nd^ chromosomes were crossed into the *dNf1^E2^* background. *UAS-RNAi* lines on the 3^rd^ chromosome were recombined with *dNf1^E2^*. *UAS-RNAi* lines in the *dNf1^E2^* background were crossed to Gal4 drivers in the same background. The few deficiencies that gave rise to synthetic lethal interactions were backcrossed with *dNf1^E1^* flies to produce Df/+; *dNf1^E2^/dNf1^E1^* progeny.

To test whether genetic suppression reflected the inadvertent introduction of a wild-type *dNf1* allele, we used fly DNA prepared using DNAzol (Molecular Research Inc.) in a PCR assay with AGTCACATTAATTGATCCTG and GAGATCGTTGATAAAGAAGT primers. The second primer introduces a penultimate single nucleotide change, which together with the E2 mutation results in the introduction of an RsaI restriction site. RsaI digestion of the PCR product gives rise to 370 and 61 bp fragments for the wild-type allele, and 348, 61 and 22 bp fragments for *the dNf1^E2^* allele. Digests were run on 8% acrylamide gels using both wild-type (*w^1118^*) and *dNf1^E2^* controls.

### Construction of *Akh-Gal4* and Attenuated UAS-PKA* Transgenes

The *Akh* promoter region was amplified with *Akh*-FORWARD (AGATCTAATCTCCTGAATGCCGCAGCG) and *Akh*-REVERSE (AGATCTATGCTGGTCCACTTCGATTC) primers. The resulting PCR fragment was subcloned into the BamHI site of a GAL4 coding region containing pCaSpeR derivative. The final construct was sequenced to ensure correct orientation of the Akh promoter before being used generate transgenic flies by standard protocols.

To reduce the toxicity associated with high-level PKA expression, we generated modified pUAS-T vectors containing 1, 2, 3 or 4, rather than 5 Gal4-binding sites. The primers used to generate these vectors were: 1×UAS-FOR: AACTGCAGAGCGGAGTACTGTCCTCCGAGCGGAGACTCTAG; 2×UAS-FOR: AACTGCAGCGGAGTACTGTCCTCCGAGCGGAGTACTGTCCTCCG; 3×UAS-FOR: AACTGCAGCGGAGTACTGTCCTCCGAGCGGAGTACTGTCCTCCGAGCGGAGTACTGTCCTCCG, and UAS-REV: CTAGAGGTACCCTCGAGCGCGGCCGCAAGAT. An initial PCR was performed using the 1×UAS-FOR and UAS-REV primers with the standard pUAS-T vector as a template. The resulting amplified fragment was TA subcloned into pCR2.1 to make pCR2.1-1×UAS. The 2×UAS-FOR and UAS-REV primers were then used with pCR2.1-UAS(1×) as a template to generate a UAS(2×) clone, which was subcloned to produce pCR2.1-UAS(2×). Similarly, 3×UAS-FOR and UAS-REV primers in a PCR reaction with pCR2.1-UAS(2×) as template generated pCR2.1-UAS(3×) and pCR2.1-UAS(4×)). The pCR2.1-UAS clones were sequenced, their inserts excised with PstI and subcloned into PstI-digested p-UAST. Correct insert orientation was verified by sequence analysis, after which the mutationally activated murine PKA* coding region [100] was subcloned into the modified vectors using XbaI and NotI.

### Immunofluorescence and Analysis of NMJ Morphology

Wandering third instar larvae were dissected in Ca^2+^-free saline and fixed in 4% paraformaldehyde for 25 min at room temperature. Following fixation, larval pelts were washed three times in phosphate-buffered saline (PBS) and then blocked for one hour in PBT (PBS+0.1% Triton-X 100)+5% normal goat serum. Larvae were incubated in primary antibody solution for three hours at room temperature. Anti-HRP 568 (1∶1000, Invitrogen) was used to visualize neurons and Alexa Fluor 488 phalloidin (1∶500, Invitrogen) was used to visualize F-actin in the musculature. Images were collected using a Yokogawa CSU-X1 spinning-disk confocal microscope with the Spectral Applied Research (Richmond Hill, ON, Canada) Borealis modification on a Nikon (Melville, NY) Ti-E inverted microscope using a 60× Plan Apo (1.4 NA) objective. The microscope was equipped with a Prior (Rockland, MA) Proscan II motorized stage. Larval samples were excited with 488-nm (for phalloidin) and 561-nm (for HRP) 100-mW solid-state lasers from a Spectral Applied Research LMM-5 laser merge module and was selected and controlled with an acousto-optical tunable filter. Emission was collected with a Semrock (Rochester, NY) quad pass (405/491/561/642 nm) dichroic mirror and 525/50 nm (for phalloidin) and 620/60 nm (for HRP) Chroma (Bellows Falls, VT) emission filters. Images were acquired using a Hamamatsu ORCA-ER-cooled CCD camera. Hardware was controlled with MetaMorph (version 7.7.9) software (Molecular Devices, Sunnyvale, CA.). Five individual animals were imaged for subsequent morphological analysis. Motor nerve terminals of muscles 6 and 7 were imaged in abdominal segments A2 and A3 and Z-stacks (0.25 µM between images) and were captured from the top to bottom of each NMJ. Morphological analysis of the NMJ was performed using NIH Image J and was assessed by quantifying the number of synaptic boutons per square micron. The number of synaptic boutons was counted as previously described [16], [101] and muscle area covered by the NMJ was quantified by tracing a polygon connecting each terminal branch point [102].

### Human NF1 Experiments

The retroviral RNAi vectors targeting human *NF1* and expression constructs of active alleles of RAS effectors were as described previously [94]. Crizotinib (S1068) and NVP-TAE648 (S1108) were purchased from Selleck Chemicals. Antibody against NF1 was from Bethyl Laboratories (A300-140A); antibodies against pAKT(S473) and ATK1/2 were from Cell Signalling; antibodies against p-ERK (E-4), ERK1 (C-16), ERK2 (C-14) and CDK4 (C-22) were from Santa Cruz Biotechnology; A mixture of ERK1 and ERK2 antibodies was used for detection of total ERK from human cell lines. Antibody against mouse PKAα-cat (A-2) SC-28315 was from Santa Cruz Biotechnology, β-Tubulin E7 from Developmental Studies Hybridoma Bank.

SH-SY5Y, Kelly and Phoenix cells were cultured in DMEM with 8% heat-inactivated fetal bovine serum, penicillin and streptomycin at 5% CO_2_. Subclones of each cell line expressing the murine ecotropic receptor were generated and used for all experiments shown. Phoenix cells were used to produce retroviral supernatants as described at http://www.stanford.edu/group/nolan/retroviral_systems/phx.html.

To measure cell proliferation, single cell suspensions were seeded into 6-well plates (1–2×10^4^ cells/well) and cultured both in the absence and presence of ALK inhibitors. At the indicated endpoints, cells were fixed, stained with crystal violet and photographed. All knockdown and overexpression experiments were done by retroviral infection as described previously [103].

The 7500 Fast Real-Time PCR System from Applied Biosystems was used to determine mRNA levels. *NF1* mRNA expression levels were normalized to expression of *GAPDH*. The following primers sequences were used in the SYBR Green master mix (Roche): *GAPDH*-Forward, AAGGTGAAGGTCGGAGTCAA; *GAPDH*-Reverse, AATGAAGGGGTCATTGATGG; *NF1*-Forward, TGTCAGTGCATAACCTCTTGC; *NF1*-Reverse, AGTGCCATCACTCTTTTCTGAAG. *ALK* mRNA levels in neurofibroma-derived *NF1^−/−^* Schwann cells and *NF1^+/−^* fibroblasts were analyzed using the following two primer sets: *ALK*-N-Forward, GGAGTGCAGCTTTGACTTCC; *ALK*-N-Reverse, TGGAGTCAGCTGAGGTGTTG; *ALK*-C-Forward, GCAACATCAGCCTGAAGACA; *ALK*-C-Reverse, GCCTGTTGAGAGACCAGGAG.

## Supporting Information

Figure S1 Loss of *dNf1* does not alter developmental timing but reduces larval growth rate. (A) Wild-type, *dNf1^E1^*, and *dNF1^E1/E2^* mutants show no altered developmental timing, as judged by their rate of pupariation (also shown in Figure 1D). By contrast, larvae with *phm-Gal4* driving *UAS-Ras1^V12^* undergo accelerated development resulting in miniature pupae [104], whereas *phm-Gal4* driving a dominant negative *UAS-PI3K^D954A^* transgene delayed development and produced giant pupae [71]. (B) Mouth hook length measurements (in µm) show that *dNf1* larvae grow at a reduced rate. The marker represents the mean length; the upper box represents the median to Q3 value, the lower box median to Q1 value and the error bars identify the outliers.
(PDF)

Figure S2 PCR/RFLP assay for *dNf1^E2^* mutation. (A) To make sure that stocks with putative suppressing deficiencies preserved the *dNf1^E2^* C->T nonsense transition, we used a PCR/Restriction Fragment Length Polymorphism assay. The E2 mutation does not create or destroy a restriction site. Rather, we used a reverse primer with a penultimate A->C transversion to amplify a 431 genomic fragment as indicated. The mutant primer creates a GTAC RsaI restriction site when E2 genomic DNA is used as a template. (B) RsaI digestion of PCR products gives rise to 370 and 61 bp fragments for the wild-type allele, and 348, 61 and 22 bp fragments for *dNf1^E2^*. An example of the assay is shown with both wild-type (*w^1118^*) and d*Nf1^E2^* controls (lanes 2, 3 and 4) and various deficiencies (Df) either in wild-type (*Df/CyO; +*; lanes 5 and 15), *dNf1* homozygous (*Df/CyO; dNf1^E2^*; lanes 6–13) or heterozygous (*Df/CyO; dNf1^E2^*/+; lanes 14 and 16) backgrounds.
(PDF)

Figure S3 Systematic identification for *dNf1* modifiers. For deficiencies that did not uncover obvious candidate modifier genes, a systematic RNAi approach was used. UAS-RNAi lines targeting genes uncovered by a modifying deficiency were driven by *Ras2-Gal4* in the *dNf1^E2^* background and the effect on pupal size determined. (A) Identification of *carnation* as a *dNf1* modifier uncovered by suppressing *Df(1)BSC275*. (B) Identification of *NAAT1* as the responsible gene uncovered by suppressing deficiencies *Df(1)BSC533* and *Df(1)Exel6290*. RNAi-induced lethality is denoted by †. Error bars show standard deviations and * indicates a *p*-value of <0.05. As part of the systematic identification of modifiers 385 RNAi lines were tested.
(PDF)

Figure S4 The *Ret* tyrosine kinase is not involved in *dNf1* growth control. (A) Reagents generated to analyze the involvement of Ret include *Ret-Gal4* transgenic lines made by inserting a 957-bp genomic segment representing the *Ret* promoter region into the pChs-*Gal4* vector. Other reagents include *UAS-Ret* transgenes harboring kinase-dead (K805A) and constitutively active (C695R) mutations made by site-directed mutagenesis. (B) *Ret-Gal4* driven *UAS-GFP* expression recapitulates the endogenous larval brain *Ret* expression pattern [60]. (C) *GMR-Gal4* driven *UAS*-*Ret* with a constitutively active C695R mutation produces a rough eye phenotype as previously reported [60]. (D) *Ret-Gal4* driven *UAS-dNf1* re-expression, RNAi-mediated *Ret* inhibition or expression of a *UAS-Ret* kinase dead transgene, all failed to modify *dNf1* pupal size. Moreover, *Ret-Gal4* driven expression of *UAS*-*Ret* with constitutively active C695R mutation failed to phenocopy the *dNf1* size defect. By contrast, a small pupal size phenocopy was observed when Ret C695R was driven ectopically with *Ras2-* and *elav-Gal4*, likely reflecting Ret-mediated activation of Ras/ERK signaling.
(PDF)

Figure S5 Expression pattern of ring gland drivers. Ring gland drivers *P0206-Gal4*, *Feb36-Gal4*, *Aug21-Gal4* and *Akh-Gal4* were crossed to *UAS-GFP*. The CNS and ring glands were dissected from third instar larvae, stained with DAPI and imaged using confocal microscopy. The prothoracic gland (PG), corpora allatum (CA) and corpora cardiaca (CC) are indicated. Specimens are orientated such that the base of the brain hemispheres is at the top, indicated by a dotted line. Scale bar = 50 µm.
(PDF)

Figure S6 ALK mRNA expression in neurofibroma-derived Schwann cells. Reverse transcription/PCR was used to analyze ALK expression in neurofibroma-derived *NF1^−/−^* Schwann cells and *NF1^+/−^* fibroblasts. Two primer sets, (A) ALK-N and (B) ALK-C, designed to amplify N-terminal and C-terminal *ALK* mRNA segments, detected *ALK* expression in *NF1^−/−^* Schwann cells, but not in *NF1^+/−^* fibroblasts. *GAPDH* primers were used as a control. To guard against positive signals due to contaminating genomic DNA, each PCR reaction was set up either with (+RT) or without (−RT) reverse transcriptase.
(PDF)

Figure S7 *NF1* suppression confers resistance to ALK inhibitors in human neuroblastoma cells. (A) Kelly cells expressing pRS and sh*GFP* controls or sh*NF1* vectors were grown in the absence or presence 200 nM NVP-TAE684 or 500 nM crizotinib. Cells were fixed, stained and photographed after 14 (untreated) or 17 (NVP-TAE684 or crizotinib-treated) days. (B) Level of *NF1* knockdown assayed by qRT-PCR. Error bars denote standard deviation.
(PDF)

Figure S8 Activation of RAS-RAF-MEK cascade confers resistance to ALK inhibitors in neuroblastoma cells. (A) Constitutively active *KRAS^V12^*, *BRAF^V600E^* or *MEK1^S218D,S222D^* mutants confer resistance to ALK inhibitors. SH-SY5Y neuroblastoma cells expressing pBabe vector control or the indicated active RAS effector mutants were grown in the absence or presence 50 nM NVP-TAE684 or 350 nM crizotinib. The cells were fixed, stained and photographed after 12 (untreated) or 19 (NVP-TAE684 and crizotinib-treated) days. (B) Level of phosphorylated ERK and AKT in the SH-SY5Y cells described above.
(PDF)

Table S1 Excluded deficiencies. Listed deficiencies were excluded for the reasons indicated. Deficiencies that failed to produce screening stocks are labeled ‘Impossible’. Unhealthy (sick) deficiencies or those that uncovered *Minute* mutations were also excluded.
(PDF)

Table S2 *dNf1* modifier deficiency screen results. All deficiencies analyzed are listed according to their relative chromosomal position. The cytological location, molecular coordinates and the dominant effect on *dNf1* pupal size (NO – no interaction, SUP - suppressor, ENH - enhancer) of each deficiency is given. Female pupal length measurements for deficiencies in the *dNf1* mutant background are provided, together with standard deviations and *p*-values. Modifying deficiencies that were subsequently found to have an effect on wild-type pupal size are indicated (Yes – indicates that a deficiency has a non-specific effect; No – no observed effect on wild-type size; No* - has an effect on wild-type size, but in the opposite direction from the effect on *dNf1* mutants). Where determined, the responsible gene identified under each modifying deficiency is shown. The final column contains notes such as deficiencies that result in altered developmental timing.
(PDF)

Table S3 Growth related genes uncovered by screened deficiencies. 18 cell, tissue, or systemic growth implicated genes uncovered by analyzed 1^st^ and 2^nd^ chromosome deficiencies. Among the deficiencies listed, only those that uncovered *dAlk* or *jeb* modified *dNf1* pupal size.
(PDF)

Table S4 Larval tissue expression patterns of Gal4 drivers. List of Gal4 driver lines used in this study and their expression patterns in third instar larvae as determined by crossing Gal4 drivers to *UAS-GFP*, or from published data. Abbreviations: Ring gland (RG), central nervous system (CNS), mushroom body (MB), prothoracic gland (PG), corpora allata (CA), corpora cardiaca (CC), neurosecretory neurons (NSNs), pars intercerebralis neurons (PI), corpora cardiaca innervating neurosecretory neuron of the medial subesophageal ganglion 2 (CC-MS 2), proventriculus (PV), fat body (FB), salivary glands (SG), imaginal discs (IDs), first instar (L1).
(PDF)

Table S5 Identification of tissues that require *dNf1* or cAMP/PKA signaling for growth regulation. Various Gal4 drivers in the *dNf1* background were crossed to *dNf1* mutants bearing attenuated UAS-PKA* transgenes or *dnc* RNAi lines. Rescue was assessed by measuring pupae, followed by genotyping adult flies upon eclosion. All crosses produced viable adults unless otherwise stated. † denotes lethality; NR non-rescue; NR* denotes non-rescue with adult eclosers with unfurled wings; n/a not applicable; n/d not determined.
(PDF)
