# Supplementary figures and images for: Genetic and Functional Studies Implicate Synaptic Overgrowth and Ring Gland cAMP/PKA Signaling Defects in the Drosophila melanogaster Neurofibromatosis-1 Growth Deficiency
Source: PLoS Genet. 2013 Nov 21;9(11):e1003958. doi: 10.1371/journal.pgen.1003958 (PMC3836801; doi:10.1371/journal.pgen.1003958)

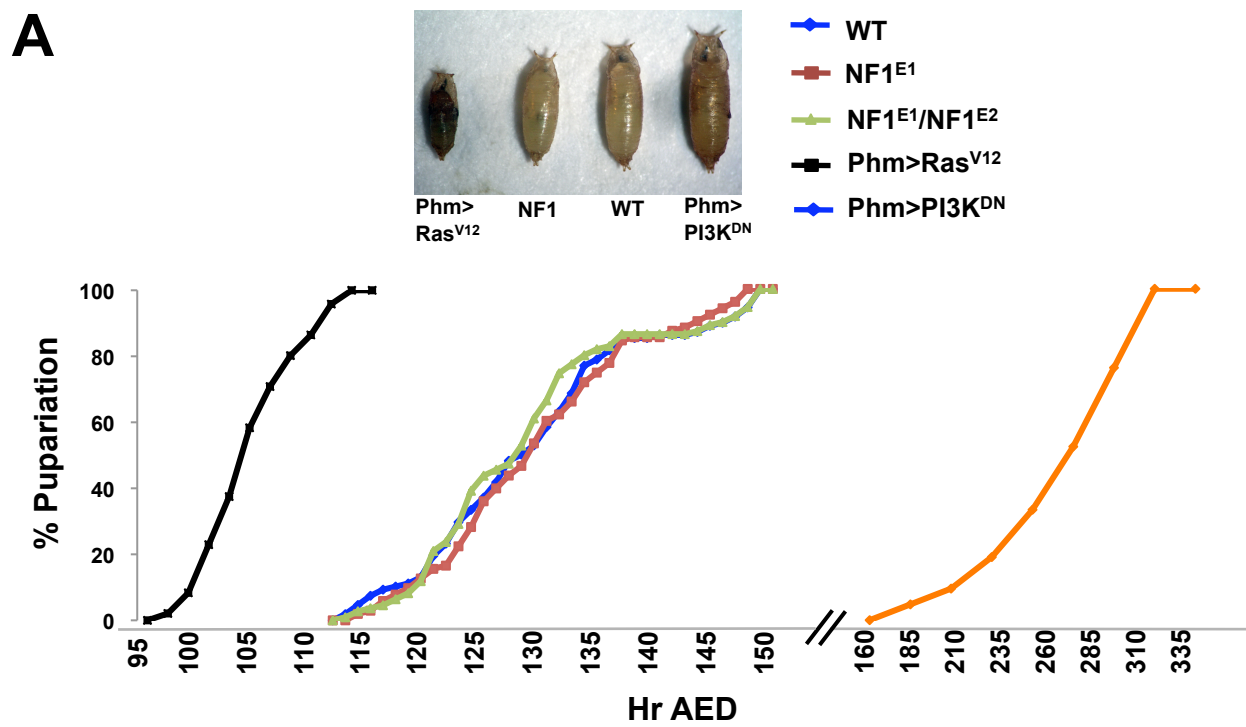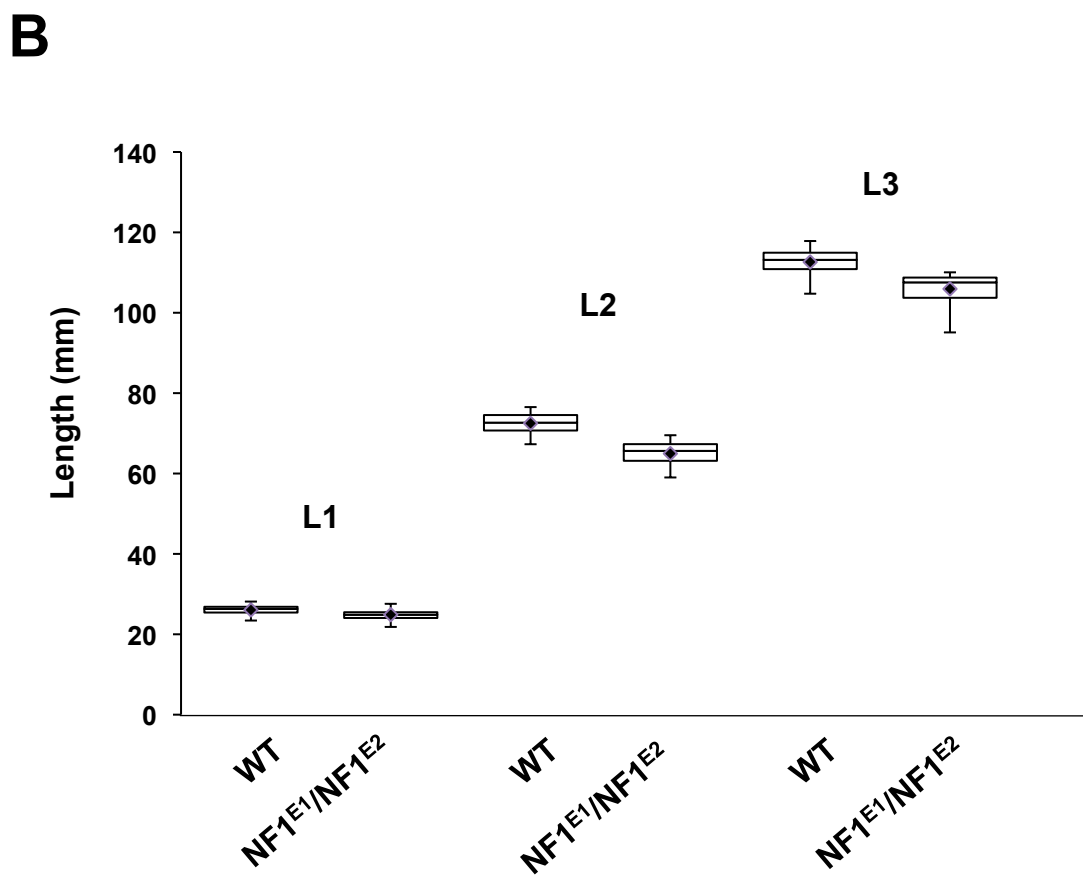

**Figure S1**

Supplement: Figure S1 — Loss of dNf1 does not alter developmental timing but reduces larval growth rate. (A) Wild-type, dNf1E1, and dNF1E1/E2 mutants show no altered developmental timing, as judged by their rate of pupariation (also shown in Figure 1D). By contrast, larvae with phm-Gal4 driving UAS-Ras1V12 undergo accelerated development resulting in miniature pupae [104], whereas phm-Gal4 driving a dominant negative UAS-PI3KD954A transgene delayed development and produced giant pupae [71]. (B) Mouth hook length measurements (in µm) show that dNf1 larvae grow at a reduced rate. The marker represents the mean length; the upper box represents the median to Q3 value, the lower box median to Q1 value and the error bars identify the outliers. (PDF) [file pgen.1003958.s001.pdf]

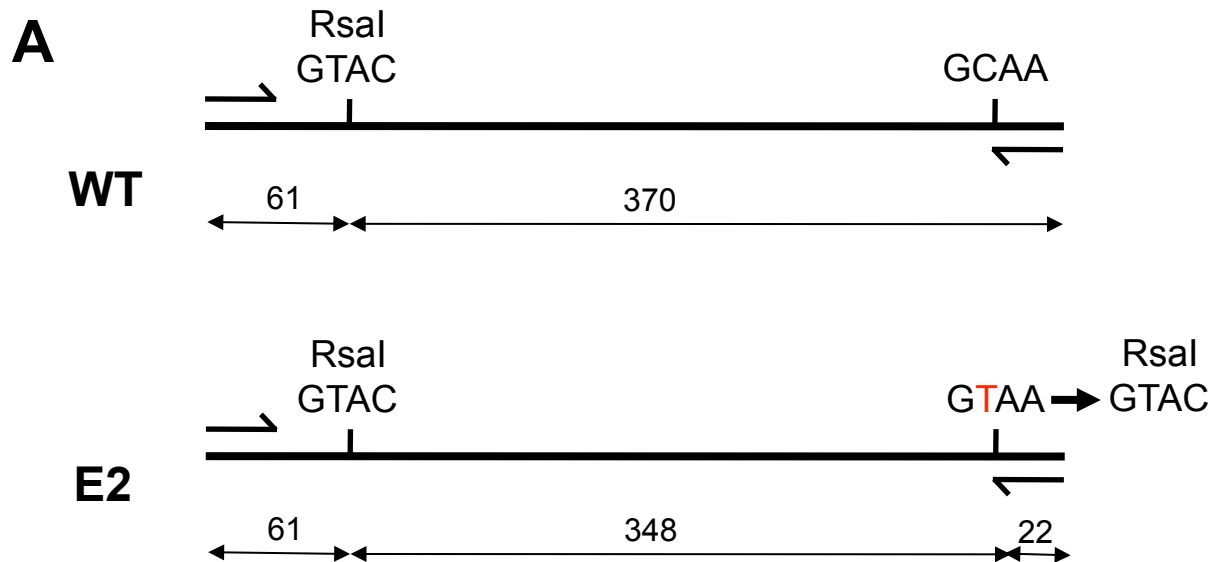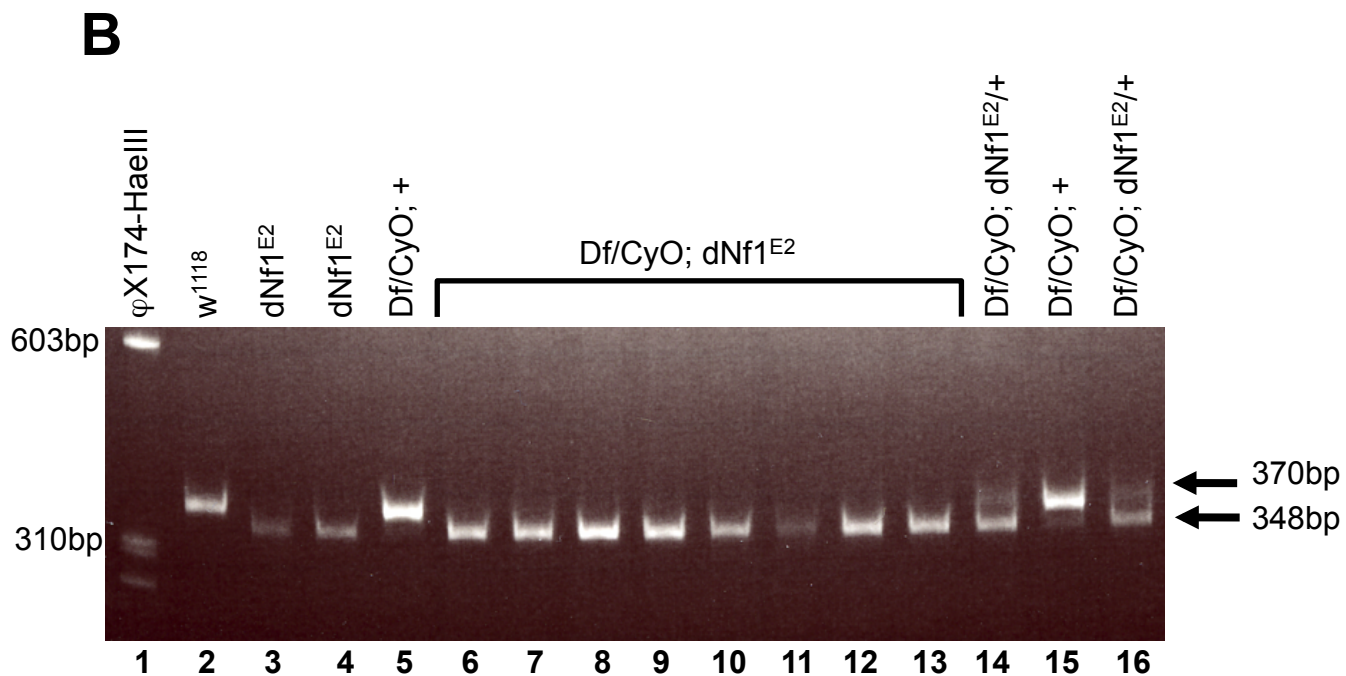

**Figure S2**

Supplement: Figure S2 — PCR/RFLP assay for dNf1E2 mutation. (A) To make sure that stocks with putative suppressing deficiencies preserved the dNf1E2 C->T nonsense transition, we used a PCR/Restriction Fragment Length Polymorphism assay. The E2 mutation does not create or destroy a restriction site. Rather, we used a reverse primer with a penultimate A->C transversion to amplify a 431 genomic fragment as indicated. The mutant primer creates a GTAC RsaI restriction site when E2 genomic DNA is used as a template. (B) RsaI digestion of PCR products gives rise to 370 and 61 bp fragments for the wild-type allele, and 348, 61 and 22 bp fragments for dNf1E2. An example of the assay is shown with both wild-type (w1118) and dNf1E2 controls (lanes 2, 3 and 4) and various deficiencies (Df) either in wild-type (Df/CyO; +; lanes 5 and 15), dNf1 homozygous (Df/CyO; dNf1E2; lanes 6–13) or heterozygous (Df/CyO; dNf1E2/+; lanes 14 and 16) backgrounds. (PDF) [file pgen.1003958.s002.pdf]

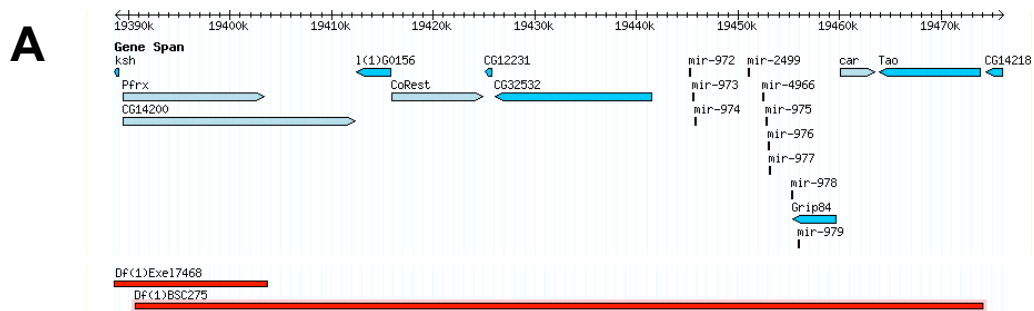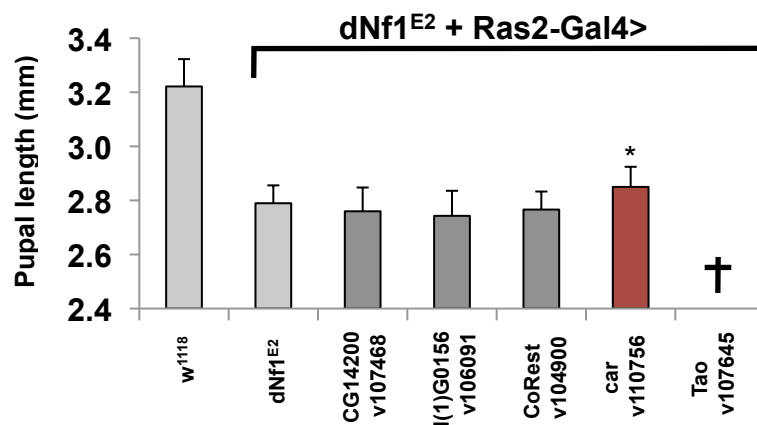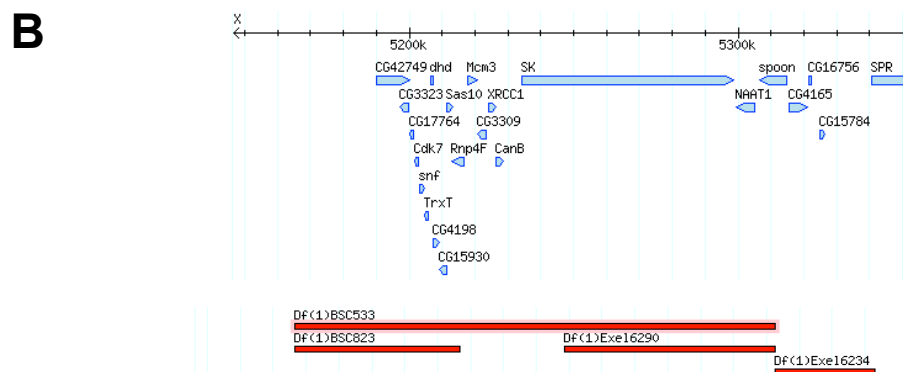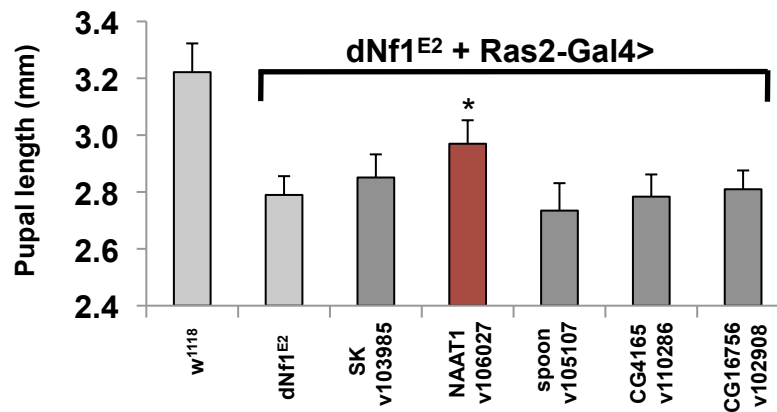

**Figure S3**

Supplement: Figure S3 — Systematic identification for dNf1 modifiers. For deficiencies that did not uncover obvious candidate modifier genes, a systematic RNAi approach was used. UAS-RNAi lines targeting genes uncovered by a modifying deficiency were driven by Ras2-Gal4 in the dNf1E2 background and the effect on pupal size determined. (A) Identification of carnation as a dNf1 modifier uncovered by suppressing Df(1)BSC275. (B) Identification of NAAT1 as the responsible gene uncovered by suppressing deficiencies Df(1)BSC533 and Df(1)Exel6290. RNAi-induced lethality is denoted by †. Error bars show standard deviations and * indicates a p-value of <0.05. As part of the systematic identification of modifiers 385 RNAi lines were tested. (PDF) [file pgen.1003958.s003.pdf]

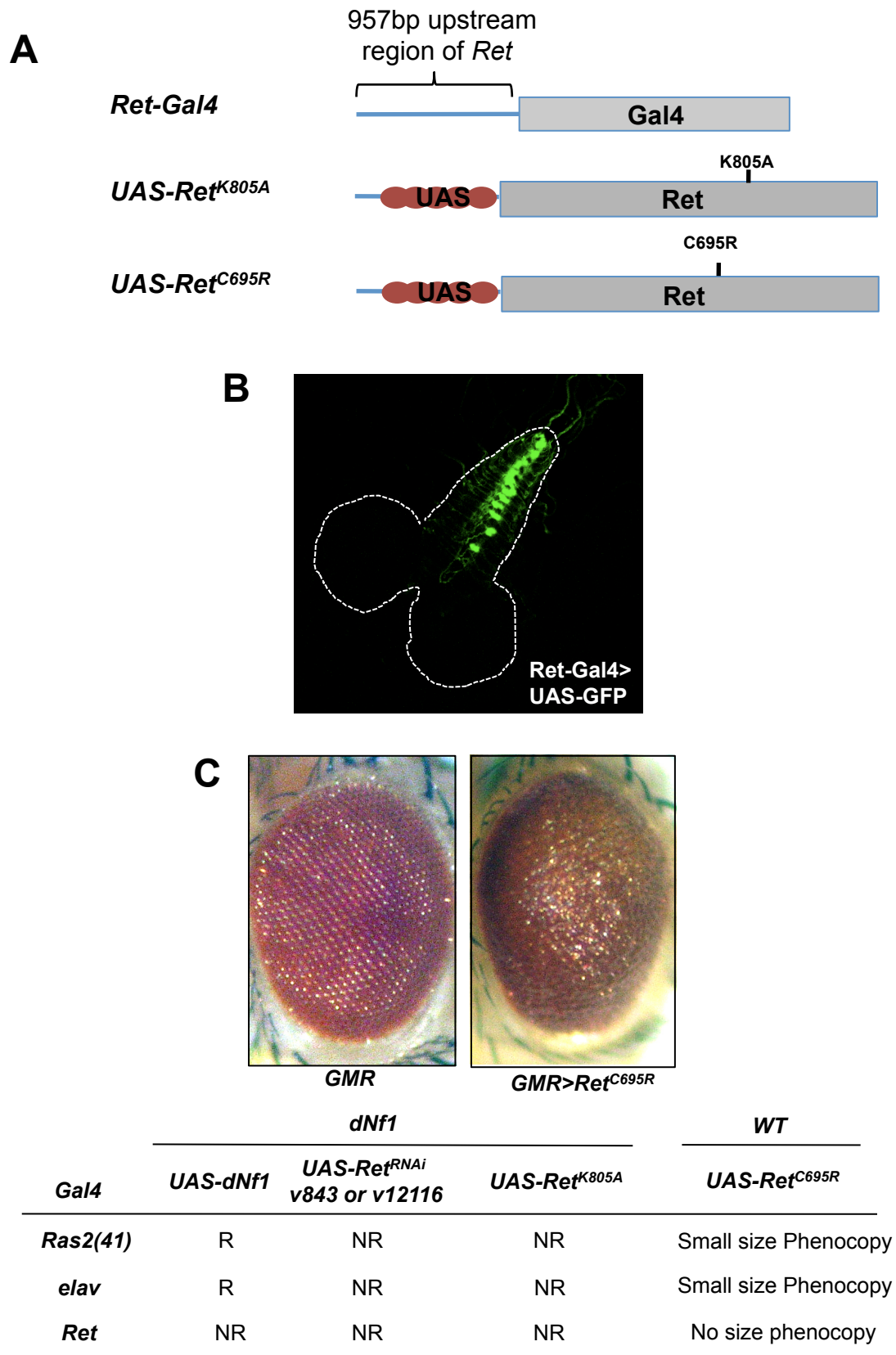

**Figure S4**

Supplement: Figure S4 — The Ret tyrosine kinase is not involved in dNf1 growth control. (A) Reagents generated to analyze the involvement of Ret include Ret-Gal4 transgenic lines made by inserting a 957-bp genomic segment representing the Ret promoter region into the pChs-Gal4 vector. Other reagents include UAS-Ret transgenes harboring kinase-dead (K805A) and constitutively active (C695R) mutations made by site-directed mutagenesis. (B) Ret-Gal4 driven UAS-GFP expression recapitulates the endogenous larval brain Ret expression pattern [60]. (C) GMR-Gal4 driven UAS-Ret with a constitutively active C695R mutation produces a rough eye phenotype as previously reported [60]. (D) Ret-Gal4 driven UAS-dNf1 re-expression, RNAi-mediated Ret inhibition or expression of a UAS-Ret kinase dead transgene, all failed to modify dNf1 pupal size. Moreover, Ret-Gal4 driven expression of UAS-Ret with constitutively active C695R mutation failed to phenocopy the dNf1 size defect. By contrast, a small pupal size phenocopy was observed when Ret C695R was driven ectopically with Ras2- and elav-Gal4, likely reflecting Ret-mediated activation of Ras/ERK signaling. (PDF) [file pgen.1003958.s004.pdf]

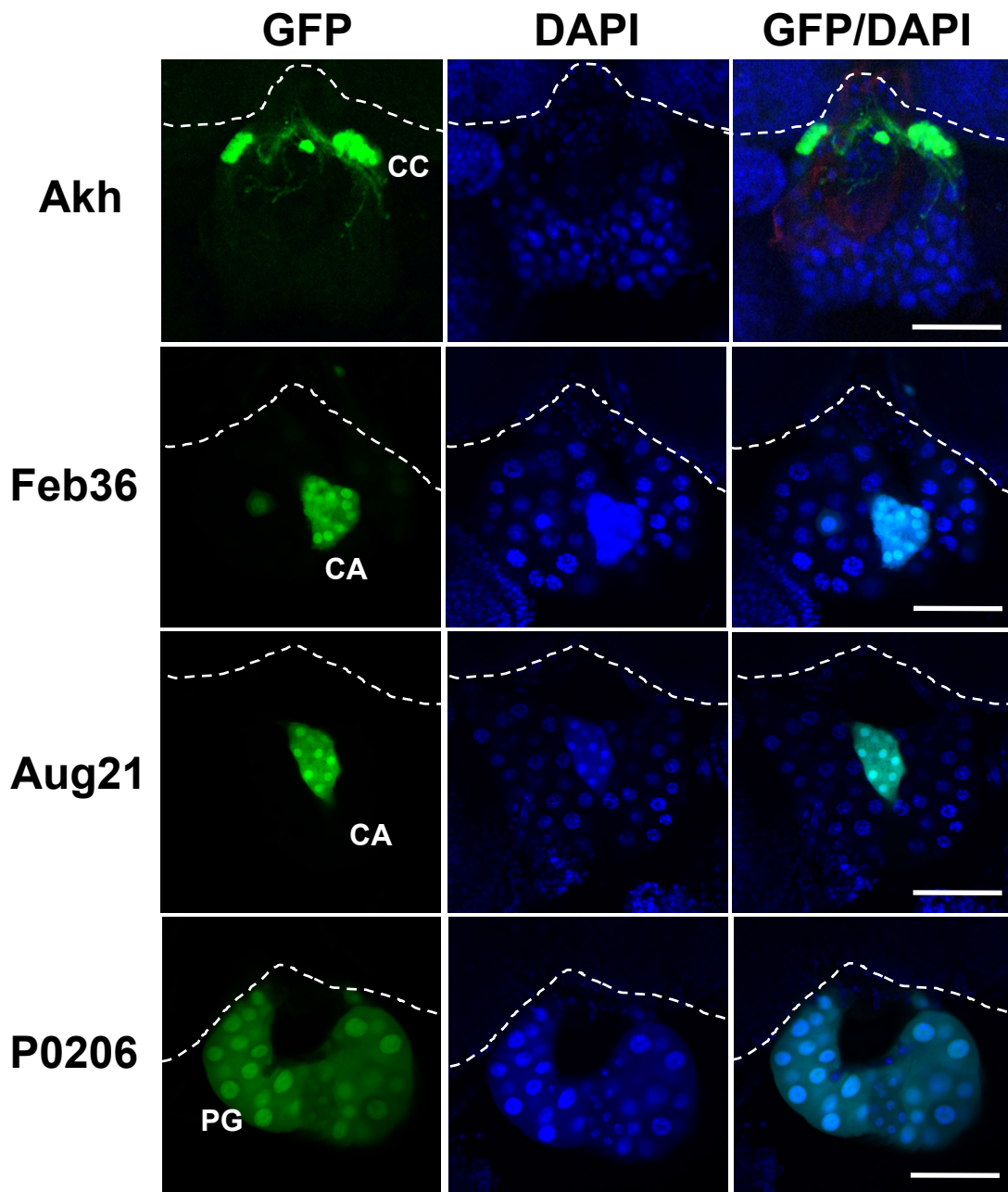

**Figure S5**

Supplement: Figure S5 — Expression pattern of ring gland drivers. Ring gland drivers P0206-Gal4, Feb36-Gal4, Aug21-Gal4 and Akh-Gal4 were crossed to UAS-GFP. The CNS and ring glands were dissected from third instar larvae, stained with DAPI and imaged using confocal microscopy. The prothoracic gland (PG), corpora allatum (CA) and corpora cardiaca (CC) are indicated. Specimens are orientated such that the base of the brain hemispheres is at the top, indicated by a dotted line. Scale bar = 50 µm. (PDF) [file pgen.1003958.s005.pdf]

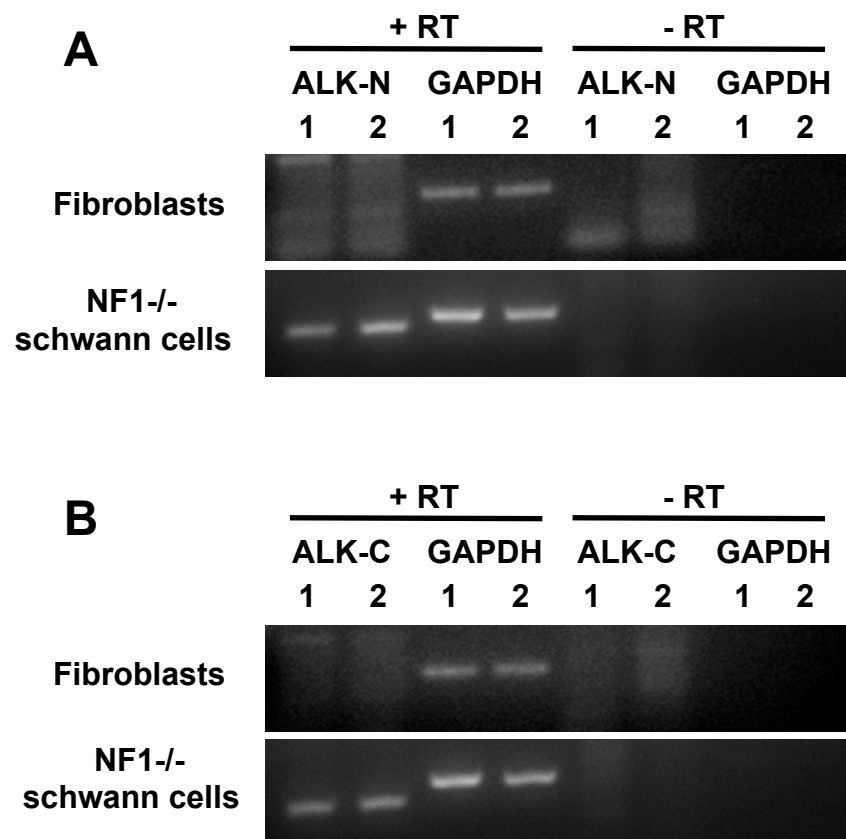

**Figure S6**

Supplement: Figure S6 — ALK mRNA expression in neurofibroma-derived Schwann cells. Reverse transcription/PCR was used to analyze ALK expression in neurofibroma-derived NF1−/− Schwann cells and NF1+/− fibroblasts. Two primer sets, (A) ALK-N and (B) ALK-C, designed to amplify N-terminal and C-terminal ALK mRNA segments, detected ALK expression in NF1−/− Schwann cells, but not in NF1+/− fibroblasts. GAPDH primers were used as a control. To guard against positive signals due to contaminating genomic DNA, each PCR reaction was set up either with (+RT) or without (−RT) reverse transcriptase. (PDF) [file pgen.1003958.s006.pdf]

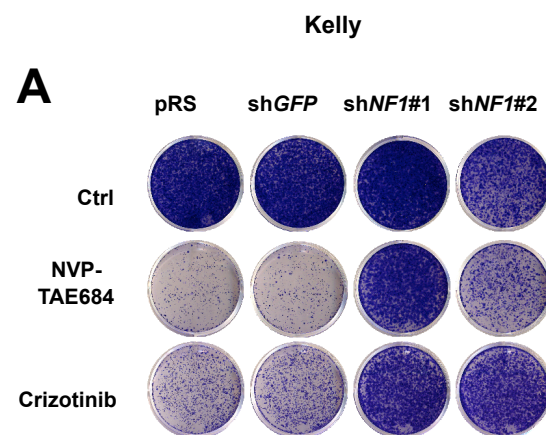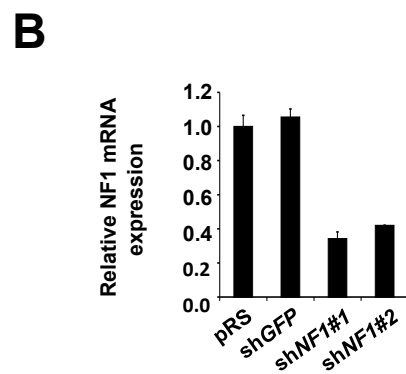

**Figure S7**

Supplement: Figure S7 — NF1 suppression confers resistance to ALK inhibitors in human neuroblastoma cells. (A) Kelly cells expressing pRS and shGFP controls or shNF1 vectors were grown in the absence or presence 200 nM NVP-TAE684 or 500 nM crizotinib. Cells were fixed, stained and photographed after 14 (untreated) or 17 (NVP-TAE684 or crizotinib-treated) days. (B) Level of NF1 knockdown assayed by qRT-PCR. Error bars denote standard deviation. (PDF) [file pgen.1003958.s007.pdf]

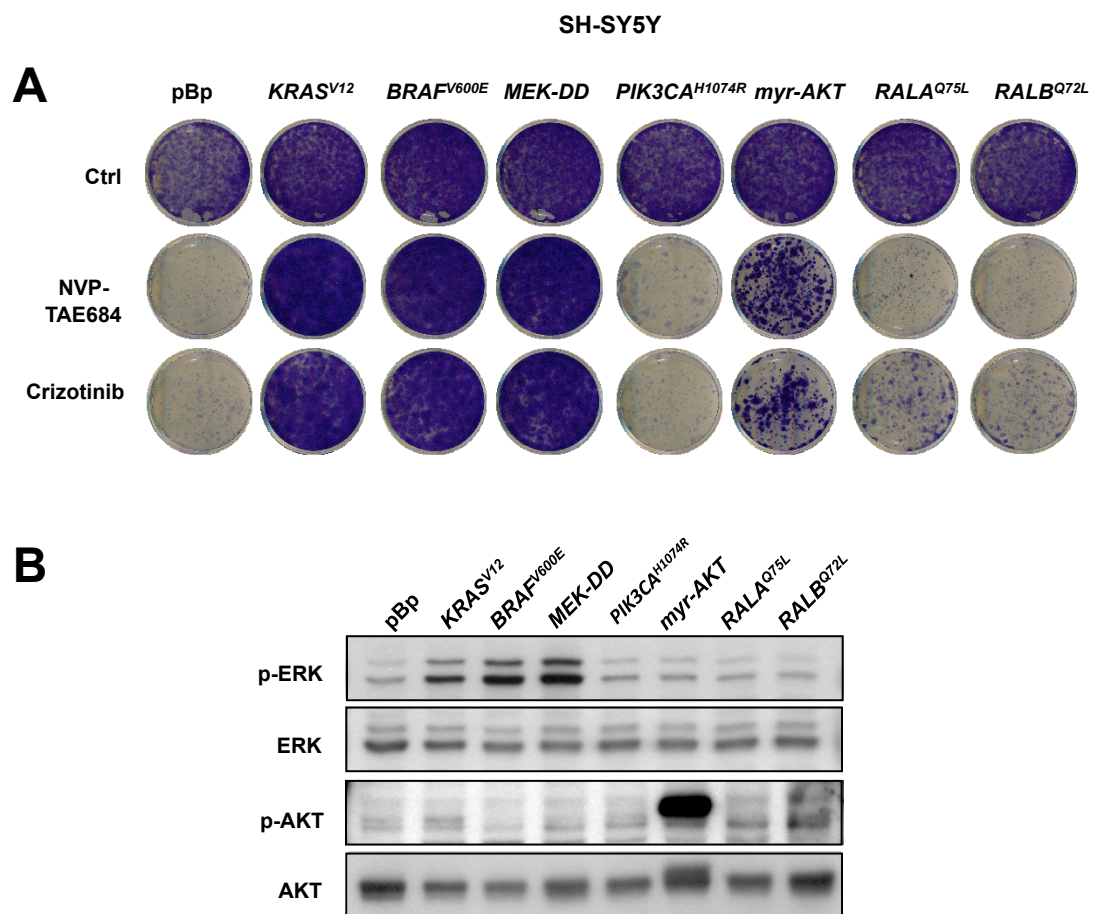

**Figure S8**

Supplement: Figure S8 — Activation of RAS-RAF-MEK cascade confers resistance to ALK inhibitors in neuroblastoma cells. (A) Constitutively active KRASV12, BRAFV600E or MEK1S218D,S222D mutants confer resistance to ALK inhibitors. SH-SY5Y neuroblastoma cells expressing pBabe vector control or the indicated active RAS effector mutants were grown in the absence or presence 50 nM NVP-TAE684 or 350 nM crizotinib. The cells were fixed, stained and photographed after 12 (untreated) or 19 (NVP-TAE684 and crizotinib-treated) days. (B) Level of phosphorylated ERK and AKT in the SH-SY5Y cells described above. (PDF) [file pgen.1003958.s008.pdf]
