# Supplementary material for: Genetic and Functional Studies Implicate Synaptic Overgrowth and Ring Gland cAMP/PKA Signaling Defects in the Drosophila melanogaster Neurofibromatosis-1 Growth Deficiency
Source: PLoS Genet. 2013 Nov 21;9(11):e1003958. doi: 10.1371/journal.pgen.1003958 (PMC3836801; doi:10.1371/journal.pgen.1003958)
Supplement: Table S1 — Excluded deficiencies. Listed deficiencies were excluded for the reasons indicated. Deficiencies that failed to produce screening stocks are labeled ‘Impossible’. Unhealthy (sick) deficiencies or those that uncovered Minute mutations were also excluded. (PDF) [file pgen.1003958.s009.pdf]

Table S1. Excluded deficiencies

Deficiency	Bloomington Stock	Breakpoints	Coordinate start	Coordinate end	Reason Discarded
**Chromosome 1**					
Df(1)BSC662	26514	7D6;7F1	8010281	8383646	Impossible
Df(1)BSC766	26863	11E9;12A7	13195231	13485587	Minute
Df(1)Exel6245	7718	11E11;11F4	13213906	13305678	Minute
Df(1)BSC714	26566	13E14;14A8	15652384	15980061	Impossible
Df(1)FDD-0230186	27408	14C1;14E1	16268326	16420365	Minute
Df(1)FDD-0230908	23296	14C6;14E1	16336383	16420365	Minute
Df(1)Exel7463	7761	17C2;17D3	18428598	18635466	Minute
Df(1)ED7413	8037	17D1;17F1	18551286	18757770	Minute
Df(1)ED7424	9350	17D1;18C1	18551286	19192806	Minute
Df(1)Exel6253	7721	18D13;18F2	19557049	19682746	Minute
**Chromosome 2L**					
Df(2L)ED62	8937	21D1;21E2	480873	826788	Sick stock
Df(2L)Exel7011	7783	22E1;22F3	2362917	2492447	Impossible
Df(2L)BSC692	26544	23B3;23B7	2830265	2868633	Minute
Df(2L)BSC692	26544	23B3;23B7	2830265	2868633	Minute
Df(2L)BSC292	23677	23F6;24A2	3515462	3632008	Impossible
Df(2L)BSC217	9694	24D8;24F1; 23D1;26C1-2	4197799	4403405	Minute
Df(2L)ED250	9270	24F4;25A7	4477085	4821294	Impossible
Df(2L)BSC52	8471	25A1--3;25B6--8	4577825	4971244	Impossible
Df(2L)ED270	8039	25F2;25F5	5658629	5800196	Long pupal phenotype in wild type
Df(2L)ED320	8902	25F2;26B2	5658629	5999643	Long pupal phenotype in wild type
Df(2L)ED279	9271	25F2;26A1	5658629	5907456	Long pupal phenotype in wild type
Df(2L)ED334	9343	25F2;26B2	5658629	5999667	Long pupal phenotype in wild type
Df(2L)ED623	8930	29C1;29E4	8403564	8700124	Impossible
Df(2L)ED776	7418	33E4;34A3	12434538	12975028	Sick stock
Df(2L)ED777	7419	33E7;34A3	12484452	12975028	Sick stock in $NF1^{E2}$
Df(2L)ED784	7421	34A4;34B6	13004448	13332060	Sick stock in $NF1^{E2}$
Df(2L)BSC159	9594	34B4;34C4; 32F;35B1-2	13290761	13536086	Minute
Df(2L)BSC812	27383	34B11;34E1	13421556	13878659	Impossible
Df(2L)ED1102	24113	35F12;36A10	16350236	16684883	Sick stock in $NF1^{E2}$
Df(2L)Exel8038	7840	36E5;36F5	18123514	18455586	Minute

Df(2L)ED1196	24115	36E6;37B1	18151698	18823590	Minute
Df(2L)ED1317	9175	38D1;38F5	20638580	20917519	Sick stock in NF1^{E2}
**Chromosome 2R**					
Df(2R)ED1484	9683	42A2;42A14	1781142	2132933	Impossible
Df(2R)ED1612	8045	42A13;42E6	2108037	2937177	Impossible
Df(2R)ED1770	9157	44D5;45B4	4543134	5095046	Sick stock
Df(2R)BSC298	23682	46B2;46C7	5621779	5845625	Sick stock
Df(2R)ED2098	9277	47A7;47C6	6304366	6786711	Sick stock
Df(2R)ED2076	8909	47A10;47C1	6364289	6707491	Impossible
Df(2R)BSC153	9540	48C1;48D7	7566603	7907386	Impossible
Df(2R)BSC361	24385	50C3;50F1	9648768	10179666	Minute
Df(2R)BSC134	9496	50E1;50E6;49A;51EF	10063602	10153306	Minute
Df(2R)BSC357	24381	50F6;51C1	10251391	10601192	Impossible
Df(2R)Exel7144	7888	53C8;53D2	12459438	12578579	Minute
Df(2R)ED3181	9213	53C9;53F10	12471454	12996322	Minute
Df(2R)ED1	6916	53E4;53F8	12914232	12984827	Sick stock
Df(2R)Exel7162	7896	56F11;56F16	16132691	16201140	Minute
Df(2R)BSC462	24966	57B5;57C8	16862884	17068495	Sick stock
Df(2R)BSC598	25431	58F3;59A1	18529284	18566186	Minute
Df(2R)BSC602	25435	60C8;60E5	20320443	20815875	Sick stock in NF1^{E2}

Listed deficiencies were excluded for the reasons indicated. Deficiencies that failed to produce screening stocks are labeled 'Impossible'. Unhealthy (sick) deficiencies or those that uncovered *Minute* mutations were also excluded.
