# Supplementary material for: Genetic and Functional Studies Implicate Synaptic Overgrowth and Ring Gland cAMP/PKA Signaling Defects in the Drosophila melanogaster Neurofibromatosis-1 Growth Deficiency
Source: PLoS Genet. 2013 Nov 21;9(11):e1003958. doi: 10.1371/journal.pgen.1003958 (PMC3836801; doi:10.1371/journal.pgen.1003958)
Supplement: Table S2 — dNf1 modifier deficiency screen results. All deficiencies analyzed are listed according to their relative chromosomal position. The cytological location, molecular coordinates and the dominant effect on dNf1 pupal size (NO – no interaction, SUP - suppressor, ENH - enhancer) of each deficiency is given. Female pupal length measurements for deficiencies in the dNf1 mutant background are provided, together with standard deviations and p-values. Modifying deficiencies that were subsequently found to have an effect on wild-type pupal size are indicated (Yes – indicates that a deficiency has a non-specific effect; No – no observed effect on wild-type size; No* - has an effect on wild-type size, but in the opposite direction from the effect on dNf1 mutants). Where determined, the responsible gene identified under each modifying deficiency is shown. The final column contains notes such as deficiencies that result in altered developmental timing. (PDF) [file pgen.1003958.s010.pdf]

Table S2. *dNf1* modifier deficiency screen results

Deficiency	Stock #	Breakpoints	Coord start	Coord end	Modification	Non-Specific Dominant Effect in WT	Pupal length (mm)	st dev	P value	n=	Gene(s) Implicated	Notes
w^{1118}							3.22	0.10		39		
$NF1^{E2}$							2.81	0.09		50		
**Chromosome 1**												
Df(1)BSC843	27887	1A1;1A3	149001	228718	NO							
Df(1)BSC530	25058	1A5;1B12	258383	517610	NO							discrepancy with Exel6221
Df(1)Exel6221	7699	1B4;1B8	364776	417700	SUPPRESSOR	No*	2.96	0.10	8.35E-06	21	elav	
Df(1)ED6396	9052	1B5;1B8	387562	417663	SUPPRESSOR	No*	3.05	0.10	1.00E-08	29	elav	
Df(1)ED6443	9053	1B14;1E1	550056	920740	NO							discrepancy with Exel6223
Df(1)Exel6223	7700	1C4;1D2	664015	803283	SUPPRESSOR	No	2.90	0.10	0.000402	20		
Df(1)ED404	8030	1D2;1E3	828975	1029478	NO							
Df(1)Exel6225	7702	1D4;1E3	880096	1029014	NO							viable deficiency
Df(1)Exel6226	7703	1E3;1F3	1028260	1216410	NO							
Df(1)ED6521	9281	1E3;1F4	1029306	1248009	NO							
Df(1)Exel6227	7704	1F3;2B1	1216410	1372852	NO							
Df(1)BSC719	26571	2A3;2B13	1347763	1759742	SUPPRESSOR	No	3.01	0.06	8.08E-07	10	dor	
Df(1)Exel8196	7769	2B1;2B5	1372852	1563287	SUPPRESSOR	No*	3.09	0.13	7.26E-07	15	dor	
Df(1)BSC589	25423	2B3;2B9	1507482	1666569	SUPPRESSOR	No	2.98	0.11	9.36E-07	11	dor	
Df(1)BSC590	25424	2B16;2E1	1834029	2088496	NO							
Df(1)ED409	8950	2C7;2F5	1936260	2211664	NO							
Df(1)BSC570	25113	2C10;2E1	1967289	2088496	NO							
Df(1)ED6574	9054	2E1;3A2	2078663	2281799	NO							
Df(1)ED11354	9345	2F6;3A4	2219975	2411834	NO							
Df(1)Exel6230	7705	3A2;3A4	2281605	2439696	SUP/non-specific	Yes	3.00	0.11	3.64E-06	14		
Df(1)Exel6231	7706	3A2;3A3	2281787	2363334	SUPPRESSOR	No	2.89	0.07	3.82E-05	25		
Df(1)ED411	8031	3A3;3A8	2363892	2536719	NO							
Df(1)ED6579	9518	3A6;3A8	2483243	2536719	NO							
Df(1)ED6584	9348	3A8;3B1	2530246	2579468	NO							
Df(1)BSC710	26562	3B2;3C9	2597988	3070426	SUPPRESSOR	No	2.90	0.11	2.84E-06	19	dunce	
Df(1)BSC656	26508	3B3;3D2	2620587	3266975	SUPPRESSOR	No	n/d				dunce	
Df(1)BSC834	27886	3C11;3F3	3182989	3739760	SUPPRESSOR	No	2.99	0.14	6.05E-05	15	dunce	
Df(1)Exel6233	7707	3D2;3D4	3266994	3379720	NO							
Df(1)ED6712	9169	3D3;3F1	3326568	3683648	NO							

Df(1)ED6716	24145	3F3;4B4	3693229	4098617	NO							
Df(1)BSC723	26575	4B6;4C7	4212091	4427231	SUPPRESSOR	n/d	2.90	0.10	7.84E-05	26		
Df(1)BSC657	26509	4B6;4C10	4218814	4530440	SUPPRESSOR	No	2.93	0.10	5.03E-06	23		
Df(1)BSC532	25060	4C1;4D2	4255593	4703149	SUP/non-specific	Yes	2.91	0.07	7.31E-08	27		
Df(1)BSC823	27584	4F4;4F5	5176614	5226804	SUPPRESSOR	No	2.99	0.10	5.78E-09	25		
Df(1)BSC533	25061	4F4;4F10	5176614	5322576	SUPPRESSOR	n/d	2.89	0.09	0.000603	20	NAAT1	
Df(1)Exel6290	7753	4F7;4F10	5258565	5322576	SUPPRESSOR	No	2.87	0.10	0.004605	24	NAAT1	
Df(1)Exel6234	7708	4F10;5A2	5322576	5410644	SUPPRESSOR	n/d	2.99	0.08	1.34E-08	18		viable deficiency
Df(1)Exel6235	7709	5A2;5A6	5410644	5487999	NO							
Df(1)BSC571	25114	5A4;5A10	5439592	5556820	NO							
Df(1)ED6802	8949	5A12;5D1	5574013	5859913	SUPPRESSOR	No	2.98	0.09	2.33E-06	16		
Df(1)Exel6236	7710	5A12;5C2	5574020	5664594	NO							
Df(1)Exel6237	7711	5C2;5C6	5664594	5786823	SUPPRESSOR	No	2.92	0.09	1.66E-08	45		
Df(1)ED418	8032	5C7;5E4	5795722	6173434	NO							
Df(1)ED6829	8947	5C7;5F3	5796009	6247128	NO							
Df(1)Exel6238	7712	5D3;5D3	5963027	6162626	NO							
Df(1)BSC640	25730	5E1;5E7	6108371	6197982	NO							
Df(1)Exel6239	7713	5F2;6B1-2	6238366	6432046	NO							
Df(1)Exel6240	7714	6B2;6C4	6437996	6563891	NO							
Df(1)BSC351	24375	6C11;6D7	6642420	6754786	NO							
Df(1)ED6878	9625	6C12;6D8	6653326	6756981	NO							
Df(1)BSC867	29990	6E4;6F1	6875892	6935548	NO							
Df(1)ED6906	8955	7A3;7B2	7089117	7299839	NO							
Df(1)BSC536	25064	7B2;7C1	7232686	7785646	SUPPRESSOR	No	2.96	0.08	9.67E-09	23		
Df(1)BSC763	26860	7B2;7B6	7329413	7586928	SUPPRESSOR	No	2.99	0.10	2.77E-06	14		
Df(1)BSC866	29989	7D5;7D16	7981026	8051355	SUPPRESSOR	No	2.91	0.10	0.000414	18		
Df(1)BSC627	25702	7F7;8B4	8486318	8764152	NO							discrepancy with Exel6241
Df(1)Exel6241	7715	8A2;8B2	8603854	8700609	SUPPRESSOR	No*	2.93	0.07	3.36E-09	28		
Df(1)ED6957	8033	8B6;8C13	8785828	9029070	NO							
Df(1)BSC663	26515	8D1;8D5	9111380	9178608	SUP/non-specific	Yes	3.09	0.08	1.56E-16	27	Dsor1/amx	
Df(1)Exel9049	7770	8D2;8D3	9141146	9146199	SUPPRESSOR	No*	2.92	0.07	1.42E-08	26	Dsor1/amx	
Df(1)BSC712	26564	8F1;9B1	9500628	9980459	NO							
Df(1)ED6989	9056	8F9;9B1	9580686	9964506	NO							
Df(1)ED6991	9216	8F9;9B4	9580686	10105557	NO							
Df(1)ED7005	9153	9B1;9D3	9965955	10479464	NO							
Df(1)BSC755	26853	9C4;9F5	10349012	10742506	SUPPRESSOR	No	2.95	0.07	1.2E-06	16		
Df(1)ED429	8034	9D3;9D3	10440897	10479464	NO							viable deficiency
Df(1)ED7010	9057	9D3;9D4	10440903	10523340	NO							
Df(1)BSC704	26556	9E4;9F13	10651741	10858005	NO							

Df(1)BSC540	25068	9E8;10A3	10666578	10959043	SUP/non-specific	Yes	2.88	0.08	4.3E-05	29		
Df(1)BSC572	25391	9F8;10A3	10784973	10986286	SUPPRESSOR	No	2.99	0.09	1.04E-07	18		
Df(1)BSC658	26510	10B3;10C10	11220142	11495012	SUPPRESSOR	No	2.98	0.14	0.002014	10		
Df(1)ED7067	9154	10B8;10C10	11284071	11495030	NO							
Df(1)BSC876	30581	10C7;10F7	11466244	11815319	NO							
Df(1)Exel6242	7716	10D1;10D7	11516418	11609816	NO							
Df(1)Exel9050	7759	10D5;10D5	11589014	11600755	NO							viable deficiency
Df(1)ED7147	9171	10D6;11A1	11608416	11898833	NO							
Df(1)ED7161	9217	11A1;11B14	11901120	12644899	NO							
Df(1)ED7153	8953	11A1;11B1	11901120	12461493	NO							
Df(1)Exel6244	7717	11A11;11B1	12357618	12441984	NO							
Df(1)BSC624	25699	11B1;11D10	12440392	12990292	NO							
Df(1)ED7170	8898	11B15;11E8	12646635	13171359	NO							
Df(1)ED7165	9058	11B15;11E1	12646635	13032981	NO							
Df(1)BSC545	25073	11C2;11E5	12679143	13117045	NO							
Df(1)ED7217	8952	12A9;12C6	13536116	13716354	NO							discrepancy with BSC546
Df(1)BSC546	25074	12B4;12B4	13612398	13641431	SUPPRESSOR	No	2.93	0.09	8.38E-07	24		
Df(1)BSC705	26557	12D1;12E9	13880813	14334444	SUPPRESSOR	No	2.96	0.08	2.41E-08	20		
Df(1)BSC628	25703	12E3;12E7	14087266	14166904	SUPPRESSOR	No	2.93	0.08	4.34E-07	25		
Df(1)ED7229	9352	12E5;12F2	14116267	14547977	ENHANCER	No	2.68	0.09	1.27E-06	20		
Df(1)ED7261	9218	12F2;12F5	14547842	14733445	NO							
Df(1)Exel6248	7719	12F4;13A1	14720026	14817700	NO							
Df(1)ED7265	9414	12F4;13A5	14720102	14901940	NO							
Df(1)BSC310	24336	12F5;13A10	14736446	14983589	SUPPRESSOR	No	2.90	0.09	4.26E-05	26		
Df(1)ED7289	29732	13A5;13A12	14918810	15019783	ENH/non-specific	Yes	2.67	0.09	4.67E-06	18		
Df(1)ED7294	8035	13B1;13C3	15069448	15344331	SUPPRESSOR	No*	2.87	0.11	0.00463	23		
Df(1)BSC706	26558	13B6;13C5	15222417	15378825	SUPPRESSOR	No	2.93	0.12	0.000233	22		
Df(1)ED7331	9219	13C3;13F1	15344288	15707556	NO							
Df(1)ED7344	9220	13E1;13F17	15520480	15762174	NO							
Df(1)BSC756	27344	13E14;13F1	15652384	15708694	NO							
Df(1)Exel6251	7720	13F1;13F17	15698387	15761875	SUPPRESSOR	n/d	3.07	0.12	2.95E-09	18		
Df(1)ED7355	8899	14A8;14B7	15985520	16172450	NO							
Df(1)ED7364	9905	14A8;14C6	15985520	16340978	NO							
Df(1)BSC761	26858	14D1;14F1	16357189	16487738	NO							
Df(1)BSC760	26857	14E1;14F2	16420365	16526135	NO							
Df(1)FDD-0172933	27413	15A1;15A11	16574754	16730272	NO							
Df(1)BSC582	25416	15A1;15E2	16574754	16985866	SUPPRESSOR	No	2.93	0.11	3.73E-08	15		
Df(1)BSC581	25415	15A3;15B1	16599184	16739816	SUPPRESSOR	No	2.97	0.09	1.27E-06	14		
Df(1)FDD-0210114	27414	15A3;15A11	16618543	16730272	NO							

Df(1)BSC725	26577	15A3;15A11	16638453	16731508	SUPPRESSOR	No	2.98	0.10	1.34E-06	18		
Df(1)BSC583	25417	15F1;16B10	17056914	17525354	SUPPRESSOR	n/d	3.04	0.10	2.1E-10	23		
Df(1)BSC853	27924	15F4;16B4	17126100	17455865	SUPPRESSOR	No	2.95	0.09	1.27E-08	25		
Df(1)BSC584	25418	15F4;16A1	17126100	17185722	NO							
Df(1)BSC641	25731	15F9;16E1	17185780	17738503	NO							
Df(1)BSC643	25733	15F9;16F1	17185780	17801269	NO							
Df(1)BSC647	25737	16C1;16F6	17605502	17957208	NO							
Df(1)BSC405	24429	16D5;16F6	17724792	17986865	NO							
Df(1)ED13478	29733	16F6;16F7	17979439	17996044	NO							
Df(1)BSC352	24376	16F7;17A8	18011500	18268918	SUP/non-specific	Yes	2.98	0.07	2.42E-10	21		
Df(1)ED447	8036	17C1;17F1	18400974	18757770	SUPPRESSOR	No*	2.97	0.10	1.66E-05	15	CCKLR-17D1	
Df(1)Exel9051	7762	17D1;17D3	18586964	18635466	SUPPRESSOR	No	2.96	0.10	1.29E-06	20	CCKLR-17D1	viable deficiency
Df(1)Exel7464	7764	17D1;17E1	18586964	18725228	SUPPRESSOR	No	2.94	0.06	1.91E-08	18	CCKLR-17D1	viable deficiency
Df(1)Exel7465	7766	17D6;17F2	18694300	18793210	NO							
Df(1)Exel6291	7754	18A2;18A2	18922751	19047666	SUPPRESSOR	No*	2.96	0.09	8.67E-08	22		viable deficiency
Df(1)ED7441	8951	18A3;18C2	19047666	19216140	NO							
Df(1)Exel9068	7767	18B4;18B6	19136043	19154050	SUPPRESSOR	No	2.96	0.09	2.41E-08	30	Hs3st-B	viable deficiency
Df(1)Exel7468	7768	18B7;18C8	19158545	19403670	NO							
Df(1)BSC275	23171	18C8;18D3	19390722	19474112	SUPPRESSOR	No	2.92	0.11	5.06E-05	25	car	
Df(1)BSC871	29994	18D7;18F2	19511634	19682746	NO							
Df(1)BSC870	29993	18E1;18F3	19584121	19742393	NO							
Df(1)ED7635	9351	19A2;19C1	19781188	20059902	NO							
Df(1)BSC645	25735	19C4;19E2	20201432	20617464	NO							
Df(1)BSC646	25736	19C4;19E4	20201432	20764608	ENHANCER	No	2.72	0.11	0.001475	18		
Df(1)Exel6254	7722	19C4;19D1	20201559	20339410	SUPPRESSOR	No*	2.99	0.08	4.24E-13	28	HERC2	
Df(1)BSC626	25701	19E1;19F4	20528438	21200049	SUPPRESSOR	No	2.92	0.08	1.02E-06	22		
Df(1)BSC708	26560	19E7;20A4	20899323	21494893	SUPPRESSOR	No	2.94	0.08	2.1E-10	30		
Df(1)ED7664	9172	19F1;19F6	20994656	21245032	NO							
Df(1)BSC588	25422	19F3;20A4	21152369	21465331	NO							
Df(1)Exel6255	7723	20A1;20C1	21390230	21919501	SUPPRESSOR	No	2.85	0.09	9.00E-12	23		
Df(1)ED12432	9156	20C3;20D2	21961362	22059220	ENH/non-specific	Yes	2.65	0.10	0.001475	17		viable deficiency and acc dev
Df(1)ED14021	9346	20C3;20F1	21961362	22282277	NO							
**Chromosome 2L**												
Df(2L)ED50001	24626	21A1;21B1	-204333	72671	ENH/non-specific	Yes	2.67	0.08	1.77E-05	17		acc dev
Df(2L)Exel6001	7488	21B1;21B2	67166	129261	NO							
Df(2L)ED5878	9353	21B1;21B3	67365	161120	NO							
Df(2L)ED929	9193	21B3;21B3	142636	161120	NO							
Df(2L)BSC454	24958	21B7;21B8	271351	307085	NO							

Df(2L)BSC107	8673	21C2;21E2	431096	574741	SUPPRESSOR	No	2.95	0.08	1.02E-08	20		
Df(2L)BSC456	24960	21D1;21E2	479689	816225	NO							
Df(2L)ED40	9188	21D1;21D2	480873	490853	NO							viable deficiency
Df(2L)Exel8003	7774	21E2;21E2	559139	715085	NO							
Df(2L)Exel6002	7489	21E2;21E2	715084	826285	NO							
Df(2L)Exel7005	7775	21E2;21E2	777148	868373	NO							
Df(2L)Exel6003	7490	21E2;21E4	826173	1074079	NO							
Df(2L)Exel6004	7491	21E4;21F1	1074079	1158137	NO							
Df(2L)ED108	24629	21F1;22A1	1119134	1420528	ENHANCER	No	2.67	0.06	4.93E-05	10		
Df(2L)Exel7006	7776	21F1;21F4	1158197	1311516	NO							
Df(2L)Exel6005	7492	22A3;22B1	1555098	1737249	NO							
Df(2L)Exel7007	7778	22B1;22B5	1716977	1909976	NO							
Df(2L)Exel8005	7779	22B2;22B8	1737960	2010136	NO							
Df(2L)Exel6006	8000	22B5;22D1	1911627	2175599	NO							
Df(2L)Exel7008	7780	22B8;22D1	1989057	2152458	NO							
Df(2L)Exel6007	7493	22D1;22E1	2175607	2362917	NO							discrepancy with Exel7010
Df(2L)Exel7010	7782	22D4;22E1	2221020	2362808	SUPPRESSOR	No	2.92	0.10	0.0002	16		
Df(2L)BSC455	24959	22D5;22E1	2242285	2374023	NO							
Df(2L)Exel6008	7494	22F4;23A3	2494660	2755377	NO							
Df(2L)Exel6277	7744	23A2;23B1	2677694	2808100	NO							
Df(2L)ED206	8038	23B8;23C5	2873954	3055717	NO							
Df(2L)Exel7014	7784	23C4;23C5	2979654	3056809	NO							
Df(2L)Exel7015	7785	23C5;23E3	3046635	3310250	NO							
Df(2L)Exel8008	7786	23E3;23E5	3302636	3354858	NO							
Df(2L)Exel7016	7787	23E5;23F3	3354818	3473493	NO							
Df(2L)Exel7018	7789	24A1;24C2	3602642	3730180	NO							
Df(2L)BSC171	9604	24C1;24C6	3713827	3825535	NO							
Df(2L)Exel6009	7495	24C3;24C8	3771368	3888977	NO							
Df(2L)BSC166	9601	24D4;24D7	4031318	4162968	NO							
Df(2L)BSC165	9600	24D4;24D8	4031318	4195308	NO							
Df(2L)BSC225	9702	25A3;25A7	4721280	4821108	NO							
Df(2L)Exel6010	7496	25A7;25B1	4820718	4887766	NO							
Df(2L)Exel9062	7792	25B1;25B1	4846961	4887766	NO							
Df(2L)Exel7021	7795	25B3;25B5	4915628	4979299	NO							
Df(2L)Exel8013	7796	25B5;25B10	4975605	5000943	NO							
Df(2L)Exel7022	7794	25B10;25C3	5000837	5058522	NO							
Df(2L)BSC109	8674	25C4;25C8	5073453	5145500	NO							
Df(2L)Exel6011	7497	25C8;25D5	5147258	5305646	NO							
Df(2L)Exel6012	7498	25D5;25E6	5305646	5555049	NO							

Df(2L)Exel7023	7797	25E5;25F1	5524375	5594234	NO						
Df(2L)Exel6256	7724	25E6;25F2	5555049	5658629	NO						
Df(2L)Exel8016	7798	25E6;25F2	5555049	5659285	NO						
Df(2L)Exel6013	7499	25F2;25F5	5658629	5805324	SUP/non-specific	Yes	n/d				
Df(2L)ED280	9179	25F5;26A1	5801930	5907456	ENHANCER	No	2.63	0.08	8.54E-07	13		viable deficiency
Df(2L)Exel6014	7500	25F5;26A3	5805324	5944680	NO						
Df(2L)Exel7024	7799	26A1;26A9	5898291	5980153	NO						
Df(2L)BSC184	9612	26B1;26B3	5980184	6051064	NO						
Df(2L)ED353	9186	26B2;26B5	6000124	6083233	ENHANCER	No	2.65	0.09	3.1E-05	16		
Df(2L)BSC239	9714	26B4;26B11	6079298	6197305	NO						
Df(2L)Exel6015	7501	26B5;26B11-C1	6088361	6262082	NO						
Df(2L)Exel6016	7502	26C1;26D1	6253010	6411492	NO						
Df(2L)Exel9038	7800	26C2;26C3	6292895	6338855	NO						
Df(2L)ED369	8903	26C3;26D1	6339087	6411333	NO						
Df(2L)BSC354	24378	26D7;26E3	6465706	6557463	ENH/non-specific	Yes	n/d					acc dev
Df(2L)BSC188	9615	26F1;27A2	6612189	6742726	ENHANCER	No	2.70	0.05	6.77E-06	24		
Df(2L)BSC187	9672	26F3;27A1	6644119	6709113	NO						
Df(2L)Exel7027	7801	26F6;27B1	6664818	6786906	NO						
Df(2L)ED6569	8940	27A1;27C4	6709099	6921292	ENHANCER	No	2.64	0.07	6.61E-07	14		
Df(2L)BSC108	8847	27C1;27C6	6874407	6945223	NO						
Df(2L)Exel7029	7802	27C4;27D4	6922143	7022707	NO						
Df(2L)BSC291	23676	27D6;27F2	7042642	7366119	NO						
Df(2L)Exel6017	7503	27E4;27F3	7202317	7418128	NO						
Df(2L)Exel7031	7804	27F2;28A3	7364976	7495492	NO						
Df(2L)ED475	9273	27F4;28B1	7423915	7576637	NO						
Df(2L)Exel6018	7504	28B1;28C1	7576630	7702880	NO						
Df(2L)Exel9031	7805	28B4;28C1	7637689	7660390	NO						
Df(2L)BSC192	9619	28C1;28D3	7702880	8003511	NO						
Df(2L)Exel7034	7807	28E1;28F1	8071311	8205166	NO						
Df(2L)BSC229	9706	28F1;29B1	8189147	8346414	NO						
Df(2L)BSC200	9627	28F5;29B1	8253607	8362842	NO						
Df(2L)ED611	9298	29B4;29C3	8382851	8419818	NO						
Df(2L)BSC201	9628	29C3;29D1	8416558	8489241	NO						
Df(2L)Exel7038	7809	29C4;29D5	8438123	8528528	SUPPRESSOR	No	2.90	0.06	1.31E-06	22		
Df(2L)BSC215	9643	29D3;29E1	8511278	8544018	SUPPRESSOR	No	2.98	0.09	3.27E-10	25		
Df(2L)Exel7039	7810	29D5;29F1	8529124	8801960	NO						
Df(2L)Exel7040	7811	29F1;29F6	8797995	8984993	NO						
Df(2L)Exel6021	7505	29F7;30A2	8989308	9176164	NO						
Df(2L)ED680	9342	30A4;30B12	9205076	9581740	ENH/non-specific	Yes	2.67	0.10	0.00026	6	Eaat1	

Df(2L)Exel8022	7813	30B1;30B4	9388129	9448833	NO							discrepancy with Exel9064
Df(2L)Exel9064	7814	30B2;30B3	9415663	9431473	SUPPRESSOR	No*	2.97	0.09	3.79E-09	20		viable deficiency
Df(2L)Exel6022	7506	30B4;30B12	9447643	9560489	NO							
Df(2L)Exel7042	7812	30B10;30C1	9522946	9622987	NO							
Df(2L)Exel6024	7507	30C1;30C9	9613611	9782218	ENHANCER	No	2.58	0.08	1.1E-09	16	PKA-C1	
Df(2L)Exel9040	7815	30C1;30C1	9613665	9622528	NO							
Df(2L)BSC216	9644	30C6;30E1	9741207	9908459	ENHANCER	No	2.66	0.07	8.88E-07	19	nAcRα-30D	
Df(2L)BSC240	9715	30C7;30F2	9744077	9960586	ENHANCER	Yes	2.62	0.06	1.56E-10	19	nAcRα-30D	
Df(2L)Exel6025	7508	30C9;30E1	9782218	9897536	ENHANCER	No	2.71	0.06	0.00017	18	nAcRα-30D	
Df(2L)Exel7043	7816	30D1;30F1	9860016	9940209	ENHANCER	n/d	2.70	0.05	2.36E-05	18	nAcRα-30D	
Df(2L)BSC205	9632	30F5;31A2	9984624	10071488	NO							
Df(2L)Exel9032	7818	31A3;31B1	10134181	10198992	NO							
Df(2L)BSC206	9633	31B1;31D9	10240295	10333704	NO							
Df(2L)Exel7046	7819	31B1;31D9	10276871	10333704	NO							
Df(2L)BSC208	9635	31D7;31D11	10321809	10381214	NO							
Df(2L)BSC342	24366	31D9;31E5	10349604	10457595	NO							
Df(2L)Exel7048	7999	31E3;31F5	10443323	10544859	NO							
Df(2L)ED746	8043	31F4;32A5	10506773	10732704	NO							
Df(2L)Exel8026	7820	31F5;32B3	10516675	10861982	NO							
Df(2L)BSC230	9707	32A5;32C1	10767145	10975285	NO							
Df(2L)Exel7049	7821	32B1;32C1	10853446	10975285	NO							
Df(2L)BSC241	9716	32C1;32F2	11006679	11445740	NO							
Df(2L)Exel6027	7510	32D2;32D5	11067029	11155825	NO							
Df(2L)BSC242	24905	32D4;32F4	11113086	11661282	NO							
Df(2L)Exel6028	7511	32D5;32E4	11155825	11358603	NO							
Df(2L)Exel6029	7512	32E4;32F2	11358603	11445762	NO							
Df(2L)BSC244	9718	32F2;33B6	11445733	12002748	SUPPRESSOR	No	2.96	0.12	5.76E-06	21		
Df(2L)BSC237	9712	32F2;33B5	11517359	11990983	NO							
Df(2L)BSC243	9717	32F3;33C1	11587693	12055450	NO							
Df(2L)Exel6030	7513	33A2;33B3	11807409	11971081	NO							
Df(2L)Exel6031	7514	33B3;33C2	11971081	12066847	NO							
Df(2L)ED775	8907	33B8;34A3	12010010	12975028	NO							
Df(2L)BSC407	24911	33D2;33F2	12175437	12657948	NO							
Df(2L)Exel6033	7516	33E4;33F2	12423459	12655793	NO							
Df(2L)Exel6034	7517	33F2;34A1	12655793	12854729	NO							
Df(2L)Exel8028	7822	34A1;34A2	12832803	12896409	NO							viable deficiency
Df(2L)Exel7055	7823	34A2;34A7	12872617	13165936	NO							
Df(2L)BSC340	24364	34B4;34B8	13290513	13369329	NO							
Df(2L)BSC147	9506	34C1;34C6	13445419	13665417	SUPPRESSOR	No	2.88	0.10	1.83E-05	55		

Df(2L)BSC691	26543	34C3;34D1	13512897	13721648	NO							
Df(2L)FDD-0428643	25166	34C3;34D6	13513468	13834944	NO							
Df(2L)Exel7059	7826	34D1;34E1	13800829	13878188	NO							
Df(2L)BSC345	24369	34E1;34E5	13878659	13952948	NO							
Df(2L)ED8186	25164	34E4;35A4	13934848	14334190	ENH/non-specific	Yes	2.66	0.06	1.92E-07	16		
Df(2L)Exel6035	7518	35A3;35B2	14300969	14470247	NO							
Df(2L)Exel6036	7519	35B1;35B2	14409711	14490657	NO							
Df(2L)Exel8033	7828	35B1;35B8	14455715	14997588	NO							
Df(2L)BSC254	23154	35B6;35C1	14813882	15074656	NO							
Df(2L)ED800	9192	35B2;35D1	14490575	15332688	ENHANCER	No	2.63	0.07	1.4E-08	18	Cul-3	
Df(2L)ED1004	25163	35B10;35D1	15061074	15333766	ENHANCER	No	2.53	0.08	3.55E-13	18	Cul-3	
Df(2L)Exel8034	7830	35C5;35D2	15264714	15439965	ENHANCER	n/d	n/d				Cul-3	
Df(2L)Exel7063	7831	35D2;35D4	15426051	15744445	NO							
Df(2L)BSC690	26542	35D4;35D4	15745455	15821840	NO							
Df(2L)Exel6038	7521	35D6;35E2	15912343	16042754	NO							
Df(2L)BSC278	23663	35E1;35F1	16025369	16289284	NO							
Df(2L)Exel7066	7833	36A1;36A12	16457328	16727482	ENH/non-specific	Yes	2.64	0.07	5.83E-07	14		
Df(2L)Exel6039	7522	36A10;36B3	16685211	16886557	ENH/non-specific	Yes	2.71	0.09	0.006038	19		
Df(2L)Exel7067	7834	36A12;36B1	16728375	16824908	ENH/non-specific	Yes	2.61	0.08	1.04E-10	25		
Df(2L)Exel8036	7835	36B1;36C9	16791487	17450255	NO							
Df(2L)Exel7068	7838	36C7;36C10	17382988	17495992	NO							
Df(2L)BSC148	9507	36C8;36E3	17428248	17965422	ENHANCER	No	2.67	0.08	3.17E-06	24		
Df(2L)Exel7069	7837	36C10;36D3	17482011	17773525	NO							
Df(2L)Exel9044	7836	36C10;36D1	17502487	17604760	NO							
Df(2L)Exel7070	7839	36E2;36E6	17903087	18161791	NO							
Df(2L)BSC256	23156	36E3;36F2	18061946	18320008	SUPPRESSOR	No	2.92	0.09	2.98E-09	54		
Df(2L)Exel9033	7841	36F2;36F2	18294845	18299279	NO							viable deficiency
Df(2L)BSC149	9508	36F5;36F10	18444727	18673286	NO							
Df(2L)Exel6041	7523	36F6;37A2	18571864	18732675	NO							
Df(2L)Exel7071	7843	37A1;37A4	18689053	18795820	NO							
Df(2L)Exel7072	7844	37A2;37B6	18753432	18943942	NO							
Df(2L)Exel7073	7845	37B1;37B9	18859186	19022139	NO							
Df(2L)Exel6042	7524	37B8;37C5	18973942	19161727	NO							
Df(2L)Exel8039	7846	37B8;37B11	18995784	19044446	NO							
Df(2L)ED1200	9173	37B9;37C5	19003398	19158447	NO							
Df(2L)BSC341	24365	37B11;37D3	19041923	19381720	NO							
Df(2L)Exel8040	7847	37C1;37C5	19110141	19161708	NO							
Df(2L)Exel6043	7525	37C5;37D7	19161727	19423709	NO							
Df(2L)BSC301	23684	37D1;37E5	19270784	19528383	NO							

Df(2L)Exel8041	7849	37D7;37F2	19426459	19586375	SUPPRESSOR	No*	2.95	0.08	1.74E-07	18	Rab9	
Df(2L)Exel9043	7913	37E1;37E1	19438065	19452918	NO							
Df(2L)Exel6044	7526	37F2;38A3	19576108	19764726	NO							
Df(2L)Exel6045	7527	38A3;38A7	19764726	19935139	SUPPRESSOR	No	3.01	0.08	6.19E-11	17		
Df(2L)Exel7077	7850	38A7;38B2	19918015	20072236	NO							
Df(2L)BSC860	29030	38B1;38C5	20035763	20310769	NO							
Df(2L)ED1305	9222	38B4;38C6	20085397	20382385	ENHANCER	No	2.65	0.09	6.12E-07	18		
Df(2L)Exel6046	7528	38C2;38C7	20205107	20458307	NO							
Df(2L)Exel7078	7851	38C7;38D5	20449190	20680624	NO							
Df(2L)Exel7079	7852	38E9;38F3	20770538	20874804	NO							
Df(2L)BSC333	24357	38F1;39A6	20831386	21155355	NO							discrepancy with BSC105 & Exel7080
Df(2L)BSC105	8671	38F2;38F4	20851900	20890383	SUPPRESSOR	No*	2.95	0.08	3.41E-06	17		
Df(2L)Exel7080	7853	38F3;39A2	20861544	21102742	SUPPRESSOR	No*	2.92	0.08	2.54E-08	37		
Df(2L)BSC302	23685	39A1;39A6	21070044	21158652	SUPPRESSOR	No	3.03	0.11	2.45E-13	31	Dap160	
Df(2L)Exel6047	7529	39A2;39B4	21102742	21244119	SUPPRESSOR	No	2.90	0.10	0.00015	19	Dap160	
Df(2L)BSC312	24338	39A4;39B2	21147512	21206341	NO							
Df(2L)Exel6048	7530	39B4;39C2	21237271	21309519	NO							
Df(2L)Exel7081	7855	39C2;39E6	21309519	21662938	NO							
Df(2L)ED1466	9340	39E3;40A5	21629316	21828548	NO							
Df(2L)Exel6049	7531	40A5;40D3	21828252	22019296	NO							
Df(2L)BSC151	9510	40A5;40E5	21828581	22139023	NO							
**Chromosome 2R**												
Df(2R)BSC630	25705	41D3;41F11	1015322	1669918	NO							
Df(2R)BSC696	26548	41F1;41F11	1304605	1669934	NO							
Df(2R)BSC697	26549	41F6;41F11	1562003	1669934	NO							
Df(2R)ED1482	23228	42A8;4A11	1923128	2019615	NO							
Df(2R)BSC326	24351	42A14;42C7	2123567	2633535	NO							
Df(2R)Exel6050	7532	42C7;42D6	2628314	2760146	NO							
Df(2R)BSC261	23161	42D1;42E5	2670129	2912551	NO							
Df(2R)Exel6051	7533	42D6;42E4	2760146	2880531	NO							
Df(2R)BSC262	23297	42D6;42F1	2789579	2994138	SUPPRESSOR	No	2.87	0.10	0.000159	63		
Df(2R)Exel6283	7748	42E5;42F2	2927526	3034554	NO							
Df(2R)BSC263	23162	42F2;43C1	3034369	3334915	NO							
Df(2R)BSC264	23163	43B2;43C5	3283390	3377339	SUPPRESSOR	No	2.97	0.07	4.34E-13	35		
Df(2R)Exel6052	7534	43C5;43E5	3380702	3510588	NO							
Df(2R)Exel6053	7535	43D3;43E9	3421058	3553300	NO							
Df(2R)Exel7092	7858	43E5;43E12	3509940	3596212	NO							
Df(2R)Exel6054	7536	43E9;43E18	3553300	3699977	NO							

Df(2R)BSC265	23164	43E16;43F4	3670332	3826552	NO							
Df(2R)Exel6055	7537	43F1;44A4	3773849	3948670	NO							
Df(2R)Exel6056	7538	44A4;44C2	3948670	4119961	NO							
Df(2R)Exel7094	7859	44A4;44B3	3948670	4019248	NO							
Df(2R)Exel7095	7860	44B3;44C2	4012164	4119968	SUP/non-specific	Yes	2.93	0.09	1.83E-08	29		
Df(2R)Exel6057	7539	44B8;44C4	4062156	4214936	NO							
Df(2R)Exel6058	7540	44C4;44D1	4215033	4332249	NO							
Df(2R)Exel7096	7862	44C6;44D3	4321177	4460278	NO							
Df(2R)Exel8047	7863	44D4;44D5	4487805	4536994	NO							
Df(2R)Exel7098	7864	44D5;44E3	4536987	4621135	SUPPRESSOR	No	3.03	0.09	2.16E-11	20		
Df(2R)BSC268	26501	44E2;45A1	4594483	4918550	SUPPRESSOR	No	2.93	0.06	4.79E-07	26		
Df(2R)BSC269	23165	44E3;44F3	4609944	4786258	NO							
Df(2R)BSC270	23166	44F9;45A2	4831372	4982207	NO							
Df(2R)BSC271	23167	44F12;45A12	4863904	5030926	SUPPRESSOR	No	2.92	0.07	4.13E-08	26		
Df(2R)BSC279	23664	45A9;45E3	5009694	5403346	SUPPRESSOR	No	2.94	0.09	2.12E-09	31		
Df(2R)BSC280	23665	45C4;45F4	5180164	5466121	NO							
Df(2R)BSC408	24912	45D4;45F4	5296452	5466121	NO							
Df(2R)BSC131	9296	46A1;46B4	5545883	5727104	SUPPRESSOR	No	2.89	0.06	5.16E-06	20		
Df(2R)Exel9016	7867	46B1;46B2	5600306	5607756	NO							
Df(2R)BSC133	9411	46B4;46C1	5724101	5763500	NO							
Df(2R)BSC152	9539	46C1;46D6	5762817	5918044	SUPPRESSOR	No	2.92	0.11	1.04E-05	28		
Df(2R)BSC158	9545	46C1;46D4	5762817	5912815	SUPPRESSOR	No	2.95	0.06	0.000642	6		
Df(2R)BSC303	23686	46E1;46F3	5965797	6035409	NO							
Df(2R)BSC281	23666	46F1;47A9	6012734	6350379	NO							
Df(2R)BSC639	25729	47B7;47F1	6605898	7176083	ENHANCER	No	2.64	0.09	1.73E-06	17		
Df(2R)BSC328	24353	47C1;47F5	6701340	7246094	NO							discrepancy with Exel 6059
Df(2R)Exel6059	7541	47C5;47D6	6761890	7073552	ENHANCER	No	2.66	0.08	4.5E-06	16		
Df(2R)BSC314	24340	47C5;47D4	6761891	6994990	NO							
Df(2R)Exel6060	7542	47D6;47F8	7073443	7285333	NO							
Df(2R)BSC231	25719	47F8;48B6	7276012	7554213	SUPPRESSOR	No	3.08	0.07	1.9E-15	17		
Df(2R)BSC160	9595	47F12;48A5	7323718	7518183	NO							
Df(2R)BSC259	23159	48A3;48C4	7480516	7711276	NO							
Df(2R)BSC329	24354	48A3;48D3	7480517	7823463	NO							
Df(2R)BSC199	9626	48C5;48E4	7779605	8059989	SUPPRESSOR	No	3.10	0.10	2.38E-11	18	jeb	
Df(2R)BSC699	26551	48D7;48E6	7907386	8070095	SUPPRESSOR	No	3.04	0.08	8.53E-10	14	jeb	
Df(2R)Exel6061	7543	48F1;49A6	8149005	8324950	NO							
Df(2R)BSC829	27911	49A1;49A9	8238453	8384335	NO							
Df(2R)BSC305	23688	49A4;49A10	8313104	8392638	NO							
Df(2R)BSC880	30585	49A9;49E1	8384335	8781621	NO							

Df(2R)Exel7121	7869	49B5;49B12	8458192	8567258	NO							
Df(2R)Exel8056	7916	49C2;49E1	8630644	8781621	NO							
Df(2R)Exel7123	7870	49D5;49E6	8749715	8868687	NO							
Df(2R)Exel6062	7544	49E6;49F1	8868689	8922684	NO							
Df(2R)Exel8057	7871	49F1;49F10	8922352	9106854	NO							
Df(2R)BSC273	23169	49F4;50A13	9047084	9389655	SUP/non-specific	Yes	2.95	0.1	0.00838	18		
Df(2R)Exel7124	7872	49F10;50A1	9106635	9168758	SUPPRESSOR	No	3.07	0.10	1.93E-08	13		
Df(2R)BSC272	23168	49F10;49F13	9106635	9136746	NO							
Df(2R)BSC306	23689	50A3;50B1	9251638	9427394	NO							
Df(2R)BSC274	23170	50A7;50B4	9317969	9480777	NO							
Df(2R)BSC307	23690	50B6;50C18	9510513	9849106	SUPPRESSOR	No	2.86	0.08	0.002652	28		
Df(2R)Exel7128	7873	50C5;50C9	9689461	9785332	SUPPRESSOR	No	3.00	0.07	2.21E-12	21		
Df(2R)BSC383	24407	50C6;50D2	9726984	9912384	SUPPRESSOR	No	2.86	0.07	0.000881	28		
Df(2R)Exel7130	7875	50D4;50E4	9960585	10100288	NO							
Df(2R)Exel7131	7876	50E4;50F6	10118170	10247931	NO							
Df(2R)BSC700	26552	50E6;51A2	10153306	10299890	NO							
Df(2R)Exel8059	7877	51A4-5;51B1	10345712	10463088	NO							
Df(2R)Exel6284	7749	51B1;51C2	10462255	10653275	NO							
Df(2R)BSC429	24933	51C2;51D1	10657714	10761429	SUPPRESSOR	No	2.93	0.06	8.03E-10	31		
Df(2R)Exel7135	7879	51E2;51E11	11017461	11150447	NO							
Df(2R)BSC346	24370	51E7;52C2	11105513	11622949	NO							
Df(2R)Exel9015	7880	51F11;51F12	11262681	11273829	NO							
Df(2R)Exel6285	7750	52A4;52B5	11371023	11563707	NO							
Df(2R)Exel9026	7881	52A13;52A13	11456133	11463338	NO							
Df(2R)Exel7137	7882	52A13-14;52C8	11463390	11746753	NO							
Df(2R)BSC308	23691	52B5;52D15	11567721	11918784	NO							
Df(2R)BSC482	24986	52C8;52D5	11748787	11838157	NO							
Df(2R)Exel7138	7883	52D1;52D12	11805928	11895238	NO							
Df(2R)ED2457	8915	52D11;52E7	11887814	12017662	NO							
Df(2R)ED2487	29661	52E6;53C4	12012439	12273917	ENH/non-specific	Yes	2.67	0.05	4.44E-12	22		
Df(2R)Exel9060	7885	52E11;52F1	12030362	12046356	NO							
Df(2R)BSC609	25442	52F6;53A5	12075259	12169783	NO							
Df(2R)Exel6063	7545	52F6;53C4	12075393	12274020	NO							
Df(2R)BSC309	23692	52F11;53B1	12117729	12176803	NO							
Df(2R)Exel7142	7886	53B1;53C4	12176759	12312794	NO							
Df(2R)BSC550	25078	53C1;53C6	12224286	12382237	NO							
Df(2R)Exel6064	7546	53C11;53D11	12499138	12716579	SUPPRESSOR	No	2.86	0.10	0.001433	20	dAlk	
Df(2R)Exel7145	7887	53D4;53D12	12618999	12734126	NO							
Df(2R)BSC382	24406	53D8;53E1	12663669	12789130	SUP/non-specific	Yes	2.97	0.07	1.46E-12	29		

Df(2R)ED2747	9278	53D11;53F8	12716578	12984808	NO							
Df(2R)ED2751	8916	53D14;53F8	12744676	12984808	NO							
Df(2R)Exel6065	7547	53D14;53D14	12744683	12985001	SUPPRESSOR	No	2.98	0.08	9.71E-13	37		
Df(2R)BSC331	24356	53D14;54A1	12756835	13027428	NO							
Df(2R)BSC359	24383	53E4;53F8	12872143	12984821	NO							
Df(2R)BSC433	24937	53F4;53F8	12950420	12984820	NO							
Df(2R)Exel6066	7548	53F8;54B6	12985074	13282147	NO							
Df(2R)BSC154	9541	54B2;54B7	13192288	13308316	NO							
Df(2R)BSC161	9596	54B2;54B17	13192288	13372333	SUPPRESSOR	No	2.95	0.07	2.24E-09	21	cnk	
Df(2R)BSC355	24379	54B16;54C3	13349852	13424178	NO							discrepancy with BSC406
Df(2R)BSC406	24430	54B16;54C1	13349852	13405632	SUPPRESSOR	No	2.96	0.08	1.3E-08	20		
Df(2R)Exel7149	7890	54C10;54D5	13469001	13579292	NO							
Df(2R)BSC347	24371	54D2;54E9	13522004	13680154	NO							
Df(2R)Exel7150	7891	54E1;54E9	13603767	13669537	NO							
Df(2R)BSC338	24362	54F1;55B1	13742225	13928168	SUPPRESSOR	No	2.92	0.06	1.93E-06	14		
Df(2R)BSC483	24987	55A1;55B7	13778659	14015980	NO							
Df(2R)BSC334	24358	55B2;55C4	13938702	14244542	SUPPRESSOR	No	2.87	0.06	0.000251	15		
Df(2R)Exel7153	7893	55B9;55C1	14038949	14118059	NO							
Df(2R)BSC337	24361	55B11;55C9	14061075	14328452	NO							
Df(2R)BSC399	24423	55D1;55E10	14391375	14618277	NO							
Df(2R)BSC339	24363	55E2;55F6	14502155	14717234	SUPPRESSOR	No*	3.13	0.09	4.32E-14	17		
Df(2R)Exel7157	7894	55E2;55E10	14509027	14618276	NO							
Df(2R)Exel7158	7895	55E9;55F6	14593031	14717233	SUPPRESSOR	No*	2.86	0.07	0.004653	19		
Df(2R)Exel6067	7549	55F8;55F8	14745547	14834784	NO							
Df(2R)Exel6068	7550	56A1;56B5	14834791	15029454	NO							
Df(2R)Exel6069	7551	56B5;56C11	15029454	15213128	NO							
Df(2R)BSC135	9423	56C11;56D5	15215243	15311082	NO							
Df(2R)BSC782	27354	56D8;56D14	15338532	15389309	NO							
Df(2R)BSC828	27910	56D10;56E1	15351032	15552519	NO							
Df(2R)BSC820	27581	56D14;56E1	15389309	15519525	NO							
Df(2R)BSC594	25678	56E1;56F9	15519529	16086559	NO							
Df(2R)BSC701	26553	56F15;57A9	16166339	16554778	SUPPRESSOR	No*	3.08	0.10	2.25E-11	18	CKIIβ2	
Df(2R)BSC702	26554	57A2;57B3	16311622	16758360	NO							
Df(2R)Exel7164	7898	57A6;57A9	16469676	16554355	NO							
Df(2R)Exel6070	7552	57A6;57B3	16470285	16723538	NO							
Df(2R)BSC403	24427	57A8;57B1	16518066	16585801	NO							
Df(2R)BSC404	24428	57A9;57B4	16554779	16770204	SUPPRESSOR	No	2.94	0.08	8.93E-07	18		
Df(2R)Exel6071	7553	57B3;57B16	16723538	16944303	NO							
Df(2R)Exel7166	7998	57B3;57B5	16758362	16887668	SUP/non-specific	Yes	3.10	0.09	1.64E-14	21	Act57B	

Df(2R)BSC814	27385	57B5;57B19	16862884	16975752	NO							
Df(2R)Exel6072	7554	57B16;57D4	16944303	17138350	NO							
Df(2R)BSC885	30590	57D2;57D10	17102728	17189303	NO							
Df(2R)BSC821	27582	57D10;57E6	17189303	17384714	SUPPRESSOR	No	2.95	0.07	4.53E-08	15		
Df(2R)Exel6076	7556	57E1;57F3	17267032	17494586	NO							
Df(2R)BSC360	24384	57E6;58A4	17384715	17857744	NO							
Df(2R)ED3923	8942	57F6;57F10	17534119	17601689	NO							
Df(2R)ED3943	9158	57F10;58D4	17600861	18289584	NO							
Df(2R)Exel6077	7557	57F10;58A3	17601473	17759533	NO							
Df(2R)Exel7169	7900	58A3;58B1	17749738	17927135	NO							
Df(2R)Exel6078	7558	58B1;58D1	17927045	18085148	NO							
Df(2R)Exel7170	7901	58B1;58C1	17927146	18023434	NO							
Df(2R)Exel7171	7902	58C1;58D2	18023507	18130755	NO							
Df(2R)Exel7173	7903	58D4;58E5	18267956	18401643	SUPPRESSOR	No	2.97	0.09	2.25E-08	19		
Df(2R)BSC787	27359	58F4;59B1	18549332	18685826	NO							
Df(2R)Exel6079	7559	59A3;59B1	18616872	18685827	NO							
Df(2R)BSC698	26550	59C1;59D11	18889174	19307192	NO							
Df(2R)Exel7177	7906	59C3;59D2	18956207	19091521	NO							
Df(2R)BSC777	27349	59C4;59E1	18997609	19331635	SUPPRESSOR	No	2.94	0.09	4.53E-08	25		
Df(2R)Exel7178	7908	59D5;59D10	19179008	19283437	NO							
Df(2R)Exel7180	7909	59E3;59F6	19431599	19573869	NO							
Df(2R)BSC659	26511	59F1;60A2	19468319	19698923	NO							
Df(2R)BSC600	25433	59F6;60A5	19601334	19752850	NO							
Df(2R)BSC770	26867	60A13;60B5	19836967	19934333	NO							
Df(2R)BSC356	24380	60B8;60C4	19955762	20145427	NO							
Df(2R)Exel6082	7561	60C4;60C7	20145420	20257300	NO							
Df(2R)BSC603	25436	60C7;60D1	20257300	20372187	NO							viable deficiency
Df(2R)BSC604	25437	60D4;60E11	20419696	20855955	NO							discrepancy with BSC607
Df(2R)BSC606	25439	60D10;60E1	20518555	20681140	NO							
Df(2R)BSC607	25440	60E4;60E8	20762378	20836711	SUPPRESSOR	No	3.01	0.12	5.57E-08	19	NKAIN	
Df(2R)BSC608	25441	60E11;60F2	20864281	20939897	NO							

All deficiencies analyzed are listed according to their relative chromosomal position. The cytological location, molecular coordinates and the dominant effect on *dNf1* pupal size (NO – no interaction, SUPPRESSOR, ENHANCER) of each deficiency is given. Female pupal length measurements for deficiencies in the *dNf1* mutant background are provided, together with standard deviations and *p*-values. Modifying deficiencies that were subsequently found to have an effect on wild-type pupal size are indicated (Yes – indicates that a deficiency has a non-specific effect; No – no observed effect on wild-type size; No* - has an effect on wild-type size, but in the opposite direction from the effect on *dNf1* mutants). Where determined, the responsible gene identified under each modifying deficiency is shown. The final column contains notes describing deficiencies that are viable and those that result in accelerated developmental timing (acc dev). The term discrepancy refers to a non-modifying deficiency that completely overlaps with a modifying deficiency.
