# Supplementary material for: Genetic and Functional Studies Implicate Synaptic Overgrowth and Ring Gland cAMP/PKA Signaling Defects in the Drosophila melanogaster Neurofibromatosis-1 Growth Deficiency
Source: PLoS Genet. 2013 Nov 21;9(11):e1003958. doi: 10.1371/journal.pgen.1003958 (PMC3836801; doi:10.1371/journal.pgen.1003958)
Supplement: Table S3 — Growth related genes uncovered by screened deficiencies. 18 cell, tissue, or systemic growth implicated genes uncovered by analyzed 1st and 2nd chromosome deficiencies. Among the deficiencies listed, only those that uncovered dAlk or jeb modified dNf1 pupal size. (PDF) [file pgen.1003958.s011.pdf]

Table S3. Growth-related genes uncovered by screened deficiencies.

Gene	Uncovered by	Reference
dAlk	*Df(2R)Exel6064*	[1]
B4	*Df(2L)BSC147; Df(2L)BSC691*	[2]
chico	*Df(2L)BSC206*	[3]
hpo	*Df(2R)BSC782; Df(2R)BSC828*	[4]
Hr4	*Df(1)BSC590*	[5]
Ilp6	*Df(1)ED6574; Df(1)ED11354*	[6,7]
jeb	*Df(2R)BSC199; Df(2R)BSC699*	[1]
Mer	*Df(1)BSC870; Df(1)BSC871*	[8]
mir-8	*Df(2R)Exel7145; Df2R)ED2747*	[9]
Pi3K21B	*Df(2L)BSC454*	[10]
Pten	*Df(2L)BSC206*	[11]
Ptth	*Df(2L)Exel8003; Df(2L)BSC456*	[12]
SNF1A	*Df(1)Exel6227*	[13]
sNPF	*Df(2L)Exel7077*	[14]
step	*Df(2L)ED1446*	[15]
Tor	*Df(2L)Exel7055*	[16]
ush	*Df(2L)ED40; Df(2L)BSC456*	[9]
yki	*Df(2R)BSC356*	[17]

18 cell, tissue, or systemic growth implicated genes uncovered by analyzed 1st and 2nd chromosome deficiencies. Among the deficiencies listed, only those that uncovered *dAlk* or *Jeb* modified *dNf1* pupal size.

1. Gouzi JY, Moressis A, Walker JA, Apostolopoulou AA, Palmer RH, et al. (2011) The receptor tyrosine kinase Alk controls neurofibromin functions in Drosophila growth and learning. PLoS genetics 7: e1002281.
2. Wittwer F, Jaquenoud M, Brogiolo W, Zarske M, Wustemann P, et al. (2005) Susi, a negative regulator of Drosophila PI3-kinase. Developmental cell 8: 817-827.
3. Bohni R, Riesgo-Escovar J, Oldham S, Brogiolo W, Stocker H, et al. (1999) Autonomous control of cell and organ size by CHICO, a Drosophila homolog of vertebrate IRS1-4. Cell 97: 865-875.

4. Harvey KF, Pfleger CM, Hariharan IK (2003) The Drosophila Mst ortholog, hippo, restricts growth and cell proliferation and promotes apoptosis. Cell 114: 457-467.
5. King-Jones K, Charles JP, Lam G, Thummel CS (2005) The ecdysone-induced DHR4 orphan nuclear receptor coordinates growth and maturation in Drosophila. Cell 121: 773-784.
6. Okamoto N, Yamanaka N, Yagi Y, Nishida Y, Kataoka H, et al. (2009) A fat body-derived IGF-like peptide regulates postfeeding growth in Drosophila. Developmental cell 17: 885-891.
7. Slaidina M, Delanoue R, Gronke S, Partridge L, Leopold P (2009) A Drosophila insulin-like peptide promotes growth during nonfeeding states. Developmental cell 17: 874-884.
8. LaJeunesse DR, McCartney BM, Fehon RG (1998) Structural analysis of Drosophila merlin reveals functional domains important for growth control and subcellular localization. The Journal of cell biology 141: 1589-1599.
9. Jin H, Kim VN, Hyun S (2012) Conserved microRNA miR-8 controls body size in response to steroid signaling in Drosophila. Genes & development 26: 1427-1432.
10. Weinkove D, Neufeld TP, Twardzik T, Waterfield MD, Leevers SJ (1999) Regulation of imaginal disc cell size, cell number and organ size by Drosophila class I(A) phosphoinositide 3-kinase and its adaptor. Current biology : CB 9: 1019-1029.
11. Goberdhan DC, Paricio N, Goodman EC, Mlodzik M, Wilson C (1999) Drosophila tumor suppressor PTEN controls cell size and number by antagonizing the Chico/PI3-kinase signaling pathway. Genes & development 13: 3244-3258.
12. McBrayer Z, Ono H, Shimell M, Parvy JP, Beckstead RB, et al. (2007) Prothoracicotropic hormone regulates developmental timing and body size in Drosophila. Developmental cell 13: 857-871.
13. Bland ML, Lee RJ, Magallanes JM, Foskett JK, Birnbaum MJ (2010) AMPK supports growth in Drosophila by regulating muscle activity and nutrient uptake in the gut. Developmental biology 344: 293-303.
14. Lee KS, You KH, Choo JK, Han YM, Yu K (2004) Drosophila short neuropeptide F regulates food intake and body size. The Journal of biological chemistry 279: 50781-50789.
15. Fuss B, Becker T, Zinke I, Hoch M (2006) The cytohesin Steppke is essential for insulin signalling in Drosophila. Nature 444: 945-948.
16. Zhang H, Stallock JP, Ng JC, Reinhard C, Neufeld TP (2000) Regulation of cellular growth by the Drosophila target of rapamycin dTOR. Genes & development 14: 2712-2724.
17. Huang J, Wu S, Barrera J, Matthews K, Pan D (2005) The Hippo signaling pathway coordinately regulates cell proliferation and apoptosis by inactivating Yorkie, the Drosophila Homolog of YAP. Cell 122: 421-434.
