# Supplementary material for: Genetic and Functional Studies Implicate Synaptic Overgrowth and Ring Gland cAMP/PKA Signaling Defects in the Drosophila melanogaster Neurofibromatosis-1 Growth Deficiency
Source: PLoS Genet. 2013 Nov 21;9(11):e1003958. doi: 10.1371/journal.pgen.1003958 (PMC3836801; doi:10.1371/journal.pgen.1003958)
Supplement: Table S4 — Larval tissue expression patterns of Gal4 drivers. List of Gal4 driver lines used in this study and their expression patterns in third instar larvae as determined by crossing Gal4 drivers to UAS-GFP, or from published data. Abbreviations: Ring gland (RG), central nervous system (CNS), mushroom body (MB), prothoracic gland (PG), corpora allata (CA), corpora cardiaca (CC), neurosecretory neurons (NSNs), pars intercerebralis neurons (PI), corpora cardiaca innervating neurosecretory neuron of the medial subesophageal ganglion 2 (CC-MS 2), proventriculus (PV), fat body (FB), salivary glands (SG), imaginal discs (IDs), first instar (L1). (PDF) [file pgen.1003958.s012.pdf]

Table S4. Larval tissue expression patterns of Gal4 drivers

Gal4	Major Larval Expression	Salivary gland	Fat body	Gut	Ring gland	Imaginal discs	Malpighian tubule	Trachea	Larval expression also in:
Ac5C	Ubiquitous	X	X	X	X	X	X	X	
elav	Pan-neuronal (not in RG)								none
Ras2(41)	CNS	X	X (weak)	X (weak)					
Ras2(19)	CNS	X	X (weak)	X (weak)	X (weak)	X (weak)			
n-syb	Pan-neuronal								none
C23	CNS: subset of neurons, MB	X	X (weak)						
386Y	Peptidergic neurons	X							
MB241	MB	X		X (midgut)					
TH	Dopaminergic neurons	X					X		PV and cuticle
Repo	Glia	X							
Ilp2	Insulin producing cells in CNS	X							
P0206	PG, CA (weak)	X		X (gut histo-blasts)					
Mai60	PG (weak)	X							central brain (weak)
Feb36	PG, CA	X	X (weak)	X (weak)			X (weak)	X (weak)	
Aug21	CA, Tv neurons in the thoracic ganglion	X							
Akh	CC								none
Feb211	PG innervating NSNs	X							
Kurs21	CA-innervating neurons	X				X (eye)			
Kurs58	PI-2 neurons	X							
Mai301	CNS: 6 NSNs in PI								cuticle
Mai369	CNS: 7 lateral NSNs innervating the CC	X	X (weak)						

Okt30	CC-MS 2	X							
Cg	Fat body		X						hemocyte,<br>anterior lymph gland pair
Lsp2	Fat body		X<br>(weak)						none
BO	L1 oenocytes								none
esg	Midgut: intestinal stem cells and enteroblasts	X		X		X (wing)			
myo1A	Gut: mature enterocytes	X		X		X			
Byn	Posterior gut	X		X					

List of Gal4 driver lines used in this study and their expression patterns in third instar larvae as determined by crossing Gal4 drivers to *UAS-GFP*, or from published data. Abbreviations: Ring gland (RG), central nervous system (CNS), mushroom body (MB), prothoracic gland (PG), corpora allata (CA), corpora cardiaca (CC), neurosecretory neurons (NSNs), pars intercerebralis neurons (PI), corpora cardiaca innervating neurosecretory neuron of the medial subesophageal ganglion 2 (CC-MS 2), proventriculus (PV), fat body (FB), salivary glands (SG), imaginal discs (IDs), first instar (L1).
