# Supplementary material for: Genetic and Functional Studies Implicate Synaptic Overgrowth and Ring Gland cAMP/PKA Signaling Defects in the Drosophila melanogaster Neurofibromatosis-1 Growth Deficiency
Source: PLoS Genet. 2013 Nov 21;9(11):e1003958. doi: 10.1371/journal.pgen.1003958 (PMC3836801; doi:10.1371/journal.pgen.1003958)
Supplement: Table S5 — Identification of tissues that require dNf1 or cAMP/PKA signaling for growth regulation. Various Gal4 drivers in the dNf1 background were crossed to dNf1 mutants bearing attenuated UAS-PKA* transgenes or dnc RNAi lines. Rescue was assessed by measuring pupae, followed by genotyping adult flies upon eclosion. All crosses produced viable adults unless otherwise stated. † denotes lethality; NR non-rescue; NR* denotes non-rescue with adult eclosers with unfurled wings; n/a not applicable; n/d not determined. (PDF) [file pgen.1003958.s013.pdf]

Table S5 Identification of tissues that require *dNf1* or cAMP/PKA signaling for growth regulation.

Gal4	UAS-*dNf1*	*dnc v18140*	*dnc v14534*	*dnc v107967*	2xUAS-*PKA**	3xUAS-*PKA**	4xUAS-*PKA**	5xUAS-*PKA**
Act5C	***Rescue***	†	***Rescue*** (pupal †)	***Rescue*** (pupal †)	Sub-viable	†	†	†
elav	***Rescue***	NR	NR	NR	NR	NR	NR	†
elav+Dcr-2	***Rescue***	NR	NR	NR	n/a	n/a	n/a	n/a
Ras2(41)	***Rescue***	NR	NR	NR	NR*	NR*	Early †	†
Ras2(19)	***Rescue***	NR	NR	NR	NR*	NR*	NR (pupal †)	†
Ras2(41)+Dcr-2	***Rescue***	NR	NR	NR	n/a	n/a	n/a	n/a
C23	***Rescue***	NR	NR	NR	NR*	NR	NR (pupal †)	†
386Y	***Rescue (weak)***	NR	NR	NR	NR	NR	NR	†
MB241	NR	NR	NR	NR	NR	NR	NR	NR
TH	NR	NR	NR	NR	NR	NR	NR	†
repo	NR	NR	NR	NR	NR	NR	NR	†
Ilp2	NR	NR	NR	NR	NR	NR (sick)	†	†
P0206	NR	NR	NR	NR	NR	NR	NR (sick)	sick
Mai60	NR	NR	NR	NR	NR	NR	NR	†
Feb36	NR	***Rescue*** (pupal †)	Sick/†	***Rescue***	NR	***Rescue (weak)***	†	†
Aug21	NR	***Rescue (weak)***	NR	***Rescue (weak)***	***Rescue (weak)***	***Rescue (weak)***	***Rescue (weak)***	†
Akh	NR	***Rescue***	***Rescue***	***Rescue***	***Rescue***	***Rescue***	***Rescue***	***Rescue (sub-viable)***
Feb211	NR	NR	NR	NR	NR	NR	NR	NR
Kurs21	NR	NR	NR	NR	NR	NR	NR	NR (very sick)
Kurs58	NR	NR	NR	NR	NR	NR	NR	NR
Mai301	NR	NR	NR	NR	NR	NR	†	†
Mai369	NR	NR	NR	NR	NR	NR	NR	†
Okt30	NR	NR	NR	NR	NR	NR	NR	n/d
Cg	NR	NR	NR	NR	NR	NR	NR	†
Lsp2	NR	NR	NR	NR	NR	NR	NR	†
BO	NR	NR	NR	NR	NR	NR	NR	NR
esg	NR	NR	NR	NR	NR	NR	NR	NR (subviable)

myoA1	NR	NR	NR	NR	NR	NR	NR	†
Byn	NR	NR	NR	NR	NR	NR (sick)	NR (pupal †)	NR (pupal †)

Various Gal4 drivers in the *dNf1* background were crossed to *dNf1* mutants bearing attenuated *UAS-PKA** transgenes or *dnc* RNAi lines. Rescue was assessed by measuring pupae, followed by genotyping adult flies upon eclosion. All crosses produced viable adults unless otherwise stated. † denotes lethality; NR non-rescue; NR* denotes non-rescue with adult eclosers with unfurled wings; n/a not applicable; n/d not determined.
